# Estimating total spending by source of funding on routine and supplementary immunisation activities in low-income and middle-income countries, 2000–17: a financial modelling study

**DOI:** 10.1016/S0140-6736(21)01591-9

**Published:** 2021-11-20

**Authors:** Gloria Ikilezi, Angela E Micah, Steven D Bachmeier, Ian E Cogswell, Emilie R Maddison, Hayley N Stutzman, Golsum Tsakalos, Logan Brenzel, Joseph L Dieleman

**Affiliations:** a Gates Ventures, Kirkland, WA, USA; b Institute for Health Metrics and Evaluation, University of Washington, Seattle, WA, USA; c Bill & Melinda Gates Foundation, Seattle, WA, USA

## Abstract

**Background:**

Childhood immunisation is one of the most cost-effective health interventions. However, despite its known value, global access to vaccines remains far from complete. Although supply-side constraints lead to inadequate vaccine coverage in many health systems, there is no comprehensive analysis of the funding for immunisation. We aimed to fill this gap by generating estimates of funding for immunisation disaggregated by the source of funding and the type of activities in order to highlight the funding landscape for immunisation and inform policy making.

**Methods:**

For this financial modelling study, we estimated annual spending on immunisations for 135 low-income and middle-income countries (as determined by the World Bank) from 2000 to 2017, with a focus on government, donor, and out-of-pocket spending, and disaggregated spending for vaccines and delivery costs, and routine schedules and supplementary campaigns. To generate these estimates, we extracted data from National Health Accounts, the WHO–UNICEF Joint Reporting Forms, comprehensive multi-year plans, databases from Gavi, the Vaccine Alliance, and the Institute for Health Metrics and Evaluation's 2019 development assistance for health database. We estimated total spending on immunisation by aggregating the government, donor, prepaid private, and household spending estimates.

**Findings:**

Between 2000 and 2017, funding for immunisation totalled US$112·4 billion (95% uncertainty interval 108·5–118·5). Aggregated across all low-income and middle-income countries, government spending consistently remained the largest source of funding, providing between 60·0% (57·7–61·9) and 79·3% (73·8–81·4) of total immunisation spending each year (corresponding to between $2·5 billion [2·3–2·8] and $6·4 billion [6·0–7·0] each year). Across income groups, immunisation spending per surviving infant was similar in low-income and lower-middle-income countries and territories, with average spending of $40 (38–42) in low-income countries and $42 (39–46) in lower-middle-income countries, in 2017. In low-income countries and territories, development assistance made up the largest share of total immunisation spending (69·4% [64·6–72·0]; $630·2 million) in 2017. Across the 135 countries, we observed higher vaccine coverage and increased government spending on immunisation over time, although in some countries, predominantly in Latin America and the Caribbean and in sub-Saharan Africa, vaccine coverage decreased over time, while spending increased.

**Interpretation:**

These estimates highlight the progress over the past two decades in increasing spending on immunisation. However, many challenges still remain and will require dedication and commitment to ensure that the progress made in the previous decade is sustained and advanced in the next decade for the Immunization Agenda 2030.

**Funding:**

Bill & Melinda Gates Foundation.

## Introduction

Decades of research have highlighted that childhood immunisations are some of the most cost-effective health interventions available.^1^, ^2^, ^3^ Estimates of lives saved, health-care costs saved, and total social costs saved per vaccine are staggering and highlight why access to safe, effective, high-quality, and affordable vaccines for all was a key global target identified in 2015 as part of the UN Sustainable Development Goals.^4^, ^5^, ^6^, ^7^ Yet, despite their known value, access to vaccines remains far from complete. As of 2019, global coverage, which captures the proportion of children who receive recommended vaccines, had remained constant for several years and an estimated 14·6 million (95% uncertainty interval [UI] 13·5–15·8) children had not received a dose of any vaccine.^8^

**Research in context**

**Evidence before this study**

Understanding health financing mechanisms in a more targeted and granular manner is important to identify and prioritise strategies to strengthen public financing systems. Although no formal literature search was done before undertaking this study, the available evidence shows that previous efforts to quantify immunisation spending comprehensively, such as those by Ozawa and colleagues and by Gandhi and colleagues, have been more forward looking, aiming to project financial costs. Additionally, those reports that do capture retrospective trends have focused on a narrow timeframe spanning the past 5 or 10 years, and relied on single data sources, either WHO's Joint Reporting Forms or comprehensive multi-year plans. As a consequence, these existing studies are limited to the weakness of each individual data source. Other studies have quantified development assistance or government spending, the scope of which has varied from illustrating spending trajectories to assessing the impact on different vaccination outcomes. Furthermore, very few studies have specifically tracked household spending on immunisation. The study by Levin and colleagues on private spending in Benin, Malawi, and Georgia highlights the variability of household spending across countries.

**Added value of this study**

Our analysis provides standardised estimates of total, government, prepaid private, and out-of-pocket spending, and development assistance for immunisations, spanning 2000–17, for 135 low-income and middle-income countries and territories. We disaggregate spending by activity and component, differentiating vaccines from delivery expenditures, and routine expenditure from that for supplementary immunisation activities. Our analyses show an increase in total immunisation spending across the study period, with the largest contributions coming from governments. We observed increases in annual disbursements for development assistance for immunisation up until 2015, after which time development assistance stagnated. Spending on routine immunisation exceeded that for supplementary immunisation activities such as mass campaigns, while investments in delivery were notably higher than those for vaccines in more recent years.

**Implications of all the available evidence**

Our study highlights the crucial role governments play in supporting the immunisation programme and demonstrates the gradual changes in development assistance contributions towards immunisation. As more low-income and middle-income countries move towards transitioning from donor support as a result of sustained economic growth, there is a need to identify sources of domestic resource mobilisation, with a targeted focus on areas traditionally supported through development assistance. Furthermore, given the additional strain on public health systems during the COVID-19 pandemic, renewed strategies should be put in place to reprioritise and maximise efficiency of available resources.

Constraints associated with the procurement of vaccines and systems to administer vaccines can be directly affected by prioritisation of financial resources for immunisation programmes.^9^, ^10^, ^11^ Consequently, the Global Vaccine Action Plan (GVAP) 2011–20^12^ and the Immunization Agenda 2030 (IA2030),^13^ each intending to catalyse progress towards universal coverage for key vaccines, have identified sustainable financing as a key need to ensure progress towards universal immunisation coverage. Strategic objective 5 of the GVAP seeks to ensure that “immunisation programmes have sustainable access to predictable funding”,^12^ whereas objective 3 of Strategic Priority Goal 6 of the IA2030 includes “secur[ing] government funding to achieve and sustain high coverage for all vaccines”.^13^

Despite these goals, there is no complete picture of immunisation financing over time. Without comprehensive and comparable immunisation spending estimates, tracking progress, holding governments accountable, identifying gaps, and advocating for more resources are challenging. Although major initiatives exist to estimate and track immunisation spending, such as the WHO–UNICEF Joint Reporting Form, WHO–UNICEF comprehensive multi-year plans for immunisation, and reports following the guidelines of the System of Health Accounts, these reporting mechanisms track different data, do not always include private spending, and are not available for a large set of countries across time. We aimed to fill this knowledge gap by drawing from and standardising spending data from all major immunisation spending reports, and using statistical estimation methods to generate estimates where underlying data are not available. We estimated annual spending on immunisations for 135 low-income and middle-income countries and territories for 2000 to 2017, with a focus on government, donor, and out-of-pocket spending, and identified whether the spending was used for vaccines or delivery costs and whether the spending was for routine immunisation schedules or supplemental campaigns.

## Methods

### Overview

For each year between 2000 and 2017, we estimated government, out-of-pocket, and donor spending on immunisations. Spending for each of these sources was estimated independently, and combined with rough estimates of prepaid private spending on immunisations to estimate total spending on immunisation for 135 low-income and middle-income countries and territories (designated according to World Bank income groups). Prepaid private spending is from private insurance or domestic philanthropy provided through non-governmental organisations (NGOs), and comprises a very small proportion of total immunisation spending. For this study, immunisation spending was defined as spending on commodities and delivery costs, including operational costs, salaries and training, transportation, and cold chain equipment meant specifically for the immunisation programme. When possible, we excluded spending on shared health-system costs such as facility or human resource costs that were used more broadly by the health system for non-immunisation activities. In general, we excluded research and development (R&D) spending, unless it was funded by an international development agency and thus assumed to be specifically for improving vaccines or delivery for low-income or middle-income countries. Using budget and expenditure data, we further disaggregated immunisation spending by two dimensions: vaccines versus delivery costs, and routine immunisation versus supplemental campaigns. All spending estimates were adjusted for inflation and then exchanged to 2019 US dollars, using inflation rates and exchange rates based on those from the International Monetary Fund.

### Estimating government spending

We extracted data from multiple sources reporting government spending for immunisation, including National Health Accounts, the Joint Reporting Form, WHO–UNICEF comprehensive multi-year plans and financial sustainability plans, co-financing by Gavi, the Vaccine Alliance, and the Immunization Delivery Cost Catalogue.^14^, ^15^, ^16^, ^17^, ^18^, ^19^ These data sources varied in scope (appendix pp 4–5). We limited our analysis to recurrent and capital spending, excluding any shared costs. From these data sources and for each country and year, we extracted five sets of estimates where they were available: total spending on immunisation, routine immunisation, supplementary immunisation, vaccines, and delivery.

From the National Health Accounts, we extracted immunisation spending estimates (categorised in the System of Health Accounts as HC.6.2) from the System of Health Accounts documents. Wherever explicit government spending on immunisation programmes or vaccines was identified, data were extracted directly and coded as such. From the WHO Global Health Expenditure Database, we extracted domestic general government expenditure on immunisation programmes, which was coded as total government spending on immunisation programmes. From available WHO–UNICEF comprehensive multi-year plans and financial sustainability plans, we extracted baseline data capturing annual historical spending on government, sub-national government, and government co-financing of Gavi vaccines. Routine recurrent and capital costs were aggregated and categorised as government spending on routine immunisation, whereas campaign costs were categorised as supplementary spending. Recurrent vaccine and injection supplies spending were aggregated and coded as vaccine spending, and non-vaccine spending coded as delivery spending. From the Joint Reporting Form, we separately extracted government spending data on routine immunisation (categorised in the Joint Reporting Form as indicator 6540) and government spending data on vaccines (categorised by the Joint Reporting Form as indicator 6510). Additional data on vaccine spending were based on Gavi co-financing data obtained from the Gavi Secretariat, whereas delivery spending estimates were based on data from the Immunization Delivery Cost Catalogue,^14^ as described in the appendix (p 6).

We implemented three techniques to standardise the data we extracted from these data sources. First, because the Joint Reporting Form data on government spending on vaccines do not include spending on vaccines used during campaigns, we leveraged data on WHO–UNICEF comprehensive multi-year plans and financial sustainability plans to scale up these data to represent total government spending on vaccines. Specifically, we calculated the proportion of total spending that comprised supplementary vaccines from the available data on WHO–UNICEF comprehensive multi-year plans and financial sustainability plans. We used a general linear model to account for missing data. We then used these proportions (observed, and where necessary fitted with the linear model) to scale up the Joint Reporting Form data. This adjustment assumes that for country-years with Joint Reporting Form data and without WHO–UNICEF comprehensive multi-year plans or financial sustainability plans, the fraction of spending that was allocated to campaigns could be modelled on the basis of data on WHO–UNICEF comprehensive multi-year plans and financial sustainability plans from other years or from other countries, or both, such that variation in this fraction followed trends and relationships observed more broadly across countries. Second, Gavi co-financing and self-financing data were scaled up as these reported values did not include spending on all vaccines on a country's national immunisation schedule. We used data on government spending on vaccines from the Joint Reporting Forms and linear mixed-effects models to standardise these data. This adjustment assumes that for country-years with Gavi co-financing and self-financing data but without Joint Reporting Form data, the fraction of immunisation spending that was allocated to Gavi-supported vaccines could be modelled according to Joint Reporting Form data from other years or other countries, or both, and that variation in this fraction followed trends and relationships observed more broadly across countries. Third, we used Cook's distance^20^, ^21^ to identify outlier data points and excluded these from additional analyses. Removing these data points assumes that these data are outliers because of measurement errors and not reflective of true trends. The covariates used to generate estimates for each model are shown in table 1. We used covariates from Financing Global Health 2019^22^ and the Global Burden of Diseases, Injuries, and Risk Factors Study 2019 (GBD 2019) data.^23^

**Table 1:** List of covariates used in model specification

	**Covariates**
Total government spending on immunisation	Surviving infant population
Government spending on routine immunisation	DTP3 vaccine coverage, maternal education per capita (years), health access and quality index, government health spending per capita
Government spending on supplementary immunisation	MCV1 coverage, surviving infant population, health access and quality index
Government spending on vaccines	Infant mortality rate, surviving infant population, maternal education per capita (years), health access and quality index, government health spending per capita
Government spending on delivery	Government health spending per capita

Government health expenditure spending per capita is from Financing Global Health 2019 data.^22^ All other covariates are from the Global Burden of Diseases, Injuries, and Risk Factors Study 2019 data.^23^ DTP3=diphtheria-tetanus-pertussis, third dose. MCV1=measles-containing vaccine, first dose.

The five immunisation components referenced above were modelled individually. We used a spatiotemporal Gaussian process regression (ST-GPR) to model estimates. ST-GPR is a flexible modelling approach that uses the observed data where they are available and reliable, and otherwise leverages data from neighbouring countries and years, as well as covariates, to generate a complete time series of estimates and to estimate uncertainty. We down-weighted data from the Immunization Delivery Cost Catalogue, WHO–UNICEF comprehensive multi-year plans, and financial sustainability plans, because the Joint Reporting Form data were more complete and consistent over time. A detailed description of ST-GPR is given in section 4.3.3 of appendix 1 of the 2020 report by the GBD 2019 Diseases and Injuries Collaborators.^23^ Model specification was determined for each of the five components of immunisation spending separately and was based on the Akaike information criterion and Bayesian information criterion, and corresponding out-of-sample root mean square error values. Model specification and parameter estimates are provided in the appendix. ST-GPR assumes missing values can be estimated by drawing information about trends and relationships between key covariates and observed estimates.

We calculated total government spending on immunisations by taking the mean of total government spending, the sum of government spending on vaccines and delivery, and the sum of government spending on routine and supplementary immunisations. To ensure internal consistency between these three entities, we raked government spending on vaccines and delivery costs and government spending on routine and supplementary immunisation to the final estimate of government spending on immunisations.

### Generating estimates of development assistance for immunisation

We used disbursement data from Gavi and other international development agencies channelling immunisation funds from 2000 to 2017. Gavi's disbursements have been classified by Gavi into 12 high-level categories, with their corresponding disbursements for each immunisation project reported. These categories comprise the cold chain equipment optimisation programme, civil society organisation, cash support, the Ebola Expanded Programme on Immunization recovery grant, graduation grant, health systems support, injection safety support, immunisation system strengthening, new vaccine support, operational support, product switch grant, and vaccine introduction grant. Additionally, these categories encompass different sub-categories. Based on the category, sub-category, and programme definitions, disbursements were categorised into the five immunisation components relevant to this study (total spending, vaccine spending, delivery costs, routine vaccine spending, and supplementary campaign spending). The appendix (pp 21–23) details the categorisation of the grants.

Disbursement data from non-Gavi funding channels were obtained from the development assistance for health database compiled by the Institute for Health Metrics and Evaluation (IHME).^24^ This database contains updated estimates of development assistance for health by funding source, channel, and health focus area. Data are typically compiled with revenue and disbursement data from online project databases, financial statements, budgets, audited reports, and through correspondence. We included only disbursing agencies that reported development assistance for vaccines or immunisation programmes. These were bilateral agencies; WHO; non-governmental organisations; UNICEF; the Bill & Melinda Gates Foundation; private, philanthropic, and charitable foundations based in the USA; the World Bank; the Asian Development Bank; and the Inter-American Development Bank. When resources were provided from one development agency to another (eg, a bilateral aid agency providing funds to Gavi), the resources were counted only for the agency receiving the resources in order to avoid counting resources twice. Methods detailing this process have been published previously and are summarised in the appendix (p 27).^22^ Development assistance for immunisations was classified into vaccine versus delivery, routine versus supplementary, as well as vaccine R&D, by use of available project-level description information. By use of a predetermined set of keywords (appendix pp 16–20) for each spending category, a search was applied to project descriptions; each disbursement was distributed into the relevant buckets based on keyword count ratios. Additional details are provided in the appendix (pp 26–27).

### Estimating out-of-pocket spending on immunisation

Existing estimates of out-of-pocket spending on immunisation were especially sparse, so we leveraged a bottom-up approach, by first calculating total spending on vaccines as the product of price (of the vaccine and the delivery of immunisation service combined) and quantity (doses of vaccines delivered), and then scaling that estimate down to reflect the fraction of the spending that was out of pocket. We used data on the doses of vaccines delivered from the Decade of Vaccine Economics Project at Johns Hopkins University^25^ and extracted price data from WHO's Market Information for Access to Vaccines project.^26^ For delivery costs, we relied on select data from the Immunization Delivery Cost Catalogue.^14^ Based on the availability of data, we limited the estimation to ten key vaccines (human papillomavirus, inactivated poliovirus, Japanese encephalitis, measles, measles-rubella, meningococcal group A, pneumococcal, rotavirus, pentavalent, and yellow fever vaccines) for which estimates of annual doses delivered were available. Furthermore, neither the volume nor price data had estimates for the entire study period. For volume, data on the ten key vaccines were available for 1692 country-years (29 328 data points available and consequently 36% missing data); for price, data were available for 255 country-years (1134 data points available and consequently 95% missing data); and for delivery cost, data were available for 26 country-years (54 data points and 99% missing data). To fill in the missing country-years of volume data, we used ST-GPR. This imputation assumes that missing estimates of volume varied according to trends and relationships observed in the 29 328 data points included. Moreover, this method assumes that out-of-pocket spending on vaccines was limited to the ten vaccines listed above. To fill in the missing observations for the price and delivery cost data, we used linear mixed-effects regression to generate regional average prices and delivery costs. We produced regional estimates for price and global estimates for delivery cost, rather than country-level estimates, because of restricted data availability. This imputation assumes that vaccine prices and delivery costs are similar for countries within the same geographical regions.

We calculated a scalar to estimate the proportion of immunisation spending (estimated by taking the product of price and volume) that was out-of-pocket spending. We leveraged data from two sources in computing this scalar. To do this, we extracted data points from a literature review focused on private spending and utilisation of immunisation, which yielded six articles of relevance and 34 estimates (appendix pp 11–12). Next, we extracted data from the Demographic Health Survey on the proportion of children younger than 5 years who used private facilities when they sought care for common childhood illnesses, specifically diarrhoea. To estimate the proportion of spending attributable to out-of-pocket spending, we adjusted country-specific estimates for the proportion of children younger than 5 years who used private facilities based on the region-specific ratio of the proportion of private facilities accessed for immunisations relative to the proportion of private facilities accessed for diarrhoea. Finally, we used ST-GPR to generate estimates of private utilisation of immunisation for all country-years within the study period. We multiplied our estimate of total spending on immunisation (from the price × volume estimation) with these scalars to obtain our estimate of out-of-pocket spending on immunisation. This method was based on the observed relationship between the fraction of immunisation spending that was paid for out of pocket and the fraction of the population seeking care for childhood diarrhoea for a child at a private clinic, and assumed that this relationship held even for countries for which we did not have information on out-of-pocket immunisation spending. We assumed out-of-pocket spending on immunisations included routine immunisation activities only (and that no out-of-pocket spending on immunisations was for supplementary campaigns). Additional details of all the covariates used in the models and years of data are provided in the appendix (p 11).

### Estimating prepaid private spending on immunisation

There were 119 data points of prepaid private spending on immunisation available from the National Health Accounts. For these data points, we made first-order estimates of prepaid private health spending on immunisation to ensure we could calculate total health spending on immunisations. To estimate prepaid private spending on immunisations, we calculated the median of the ratio of prepaid private spending for immunisations measured as a share of total immunisation spending (per country and year) relative to prepaid private spending in the entire health sector measured as a share of total health sector spending. We then used this information (and the complete set of health sector prepaid private health spending estimates from the IHME Health Expenditure database) to estimate prepaid private spending on immunisations for each country.^27^ This estimation assumes that the relationship between the fraction of immunisation spending that is prepaid private health spending and the fraction of health spending that is prepaid private health spending is relatively constant across countries and time.

### Estimating total spending on immunisation and uncertainty intervals

We estimated total spending on immunisation by aggregating the government, donor, household, and prepaid private spending estimates on immunisation activities. We report immunisation spending estimates and immunisation spending per surviving infant. We calculated estimates of the number of surviving infants using data on livebirths and infant mortality from GBD 2019. The estimates are reported by GBD super-region and Gavi co-financing category. GBD super-regions categorise countries into seven regions according to cause of death patterns. For these aggregates, spending was summed across countries and then divided by the total population to reflect the average of spending levels with these aggregates, rather than the mean across these countries. We generated 95% UIs by taking the 2·5th and 97·5th percentile of the 1000 estimated random draws. Uncertainty was calculated for each of the five modelled entities for government spending, estimates of price and volume, and the out-of-pocket spending ratio for out-of-pocket spending estimates, and resulting variation in the prepaid private spending estimates. Development assistance for immunisation estimates was not reported with associated 95% UIs as the estimates were made on the basis of accounting methods rather than statistical estimation. The study period was limited to 2000 to 2017 due to data lags in the availability of key input data sources. We completed all analyses using Stata (versions 13 and 15) and R (versions 3.6.0 and 3.6.1).

### Role of the funding source

The funder of this study had no role in study design, data collection, data analysis, data interpretation, or writing of the report.

## Results

**Figure 1:**
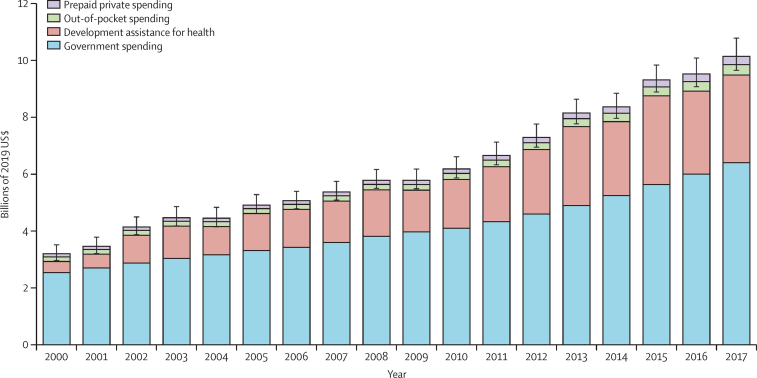
Total spending on immunisation by financing source, 2000–17 Spending estimates are presented in inflation-adjusted 2019 US dollars. Total spending estimates: $3·2 billion in 2000 and $10·2 billion in 2017. Immunisation spending in low-income and middle-income countries increased from $5·9 billion (95% UI 5·6–6·2) in 2010 to $9·6 billion (9·2–10·1) in 2017, with 55·9% (50·6–60·5) of the increase coming from domestic governments. Error bars denote 95% uncertainty intervals (UIs) of total spending.

We found substantial variation in immunisation spending patterns over time, with 132 (97·8%) of 135 countries and territories increasing spending, and three (2·2%) countries decreasing spending (Venezuela, Syria, and Samoa), with the largest increases seen in Tajikistan, Afghanistan, and the Democratic Republic of the Congo. The increasing trends in total immunisation spending by source from 2000 to 2017 are shown in figure 1. Overall, funding for immunisation totalled $112·4 billion (95% UI 108·5–118·5) between 2000 and 2017. Government spending consistently remained the largest source of funding throughout our study period, providing between 60·0% (57·7–61·9) and 79·3% (73·8–81·4) of total immunisation spending, corresponding to between $2·5 billion (2·3–2·8) in the lowest year and $6·4 billion (6·0–7·0) in the highest year.

Development assistance was the second largest source of immunisation spending, accounting for 28·2% (95% UI 26·7–29·2), or $31·7 billion, of the total immunisation spending over the study period. Of this, around $13·3 billion was channelled through Gavi, while approximately $18·3 billion was disbursed by other development agencies. In addition to Gavi, UN agencies were the most prominent agencies through which immunisation programmes were funded, with UNICEF accounting for 23·8% ($4·3 billion) of non-Gavi development assistance for health and WHO accounting for 24·4% ($4·4 billion). Bilateral agencies provided a similar amount of funding, at 15·8% ($2·9 billion), whereas the Bill & Melinda Gates Foundation disbursed 19·7% ($3·6 billion) directly. Additionally, NGOs disbursed 15·3% ($2·8 billion), and the World Bank disbursed 0·5% ($87·4 million), whereas disbursements from the European Commission, development banks, and US-based charitable and philanthropic foundations accounted for 0·6% ($104·3 million). In 2017 alone, development assistance for immunisation spending was estimated at $3·1 billion, with $1·5 billion from Gavi and $1·6 billion from other channels. The largest share of development assistance that can be traced to a specific geographical region was targeted to sub-Saharan Africa.

Out-of-pocket spending remained relatively stable up to 2010, after which we observed substantial growth, with spending in 2017 amounting to double the amount spent in 2000. Out-of-pocket spending totalled $4·0 billion (95% UI 2·8–9·0) throughout our period of study. Prepaid private spending was the smallest source of funding for immunisation, totalling $3·0 billion (95% UI 2·8–3·3) throughout the study period.

**Figure 2:**
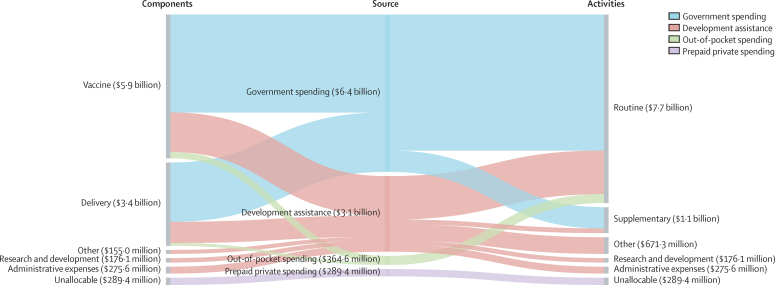
Flows of total immunisation spending from immunisation component to financing source to immunisation activities, 2017 Values are reported in inflation-adjusted 2019 US dollars. “Other” captures immunisation spending for which we have information on immunisation component or immunisation activities but which is not identified as being allocated to any of the components or activities listed. Spending for which we have no information on immunisation component or immunisation activities is designated as “Unallocable”.

The allocation of immunisation spending in 2017 by source, disaggregated by component and by activity, is shown in figure 2. In 2017, $6·4 billion (95% UI 6·0–7·0) was provided by government sources, $4·0 billion (3·7–4·4; 62·2% [58·6–65·6]) of which was allocated to vaccines, and $2·4 billion (2·2–2·7; 37·8% [34·4–41·4]) to delivery. The bulk of government spending was allocated to routine immunisation, making up 86·5% (82·1–90·1; $5·6 billion [5·2–6·0]) of the total, compared to supplementary immunisation activities, which represented 13·5% (9·9–17·9; $0·9 billion [0·6–1·2]) of spending. We observed similar spending patterns for development assistance, where 57·5% ($1·8 billion) of the $3·1 billion was allocated to routine immunisation and 6·1% (or $0·2 billion) to supplementary activities. Vaccines accounted for 52·3% ($1·6 billion) of spending and delivery accounted for 28·0% ($0·9 billion). Additional categories of development assistance spending included R&D and administrative expenses, which collectively made up 14·6% of spending, and non-specified immunisation spending, which comprised 21·8% of spending. Out-of-pocket spending for vaccines in routine services accounted for 75·1% (35·2–92·0; $256 million [221–296]) of spending, whereas out-of-pocket spending on delivery activities was 24·9% (8·0–64·8; $108 million [22–449]).

**Figure 3:**
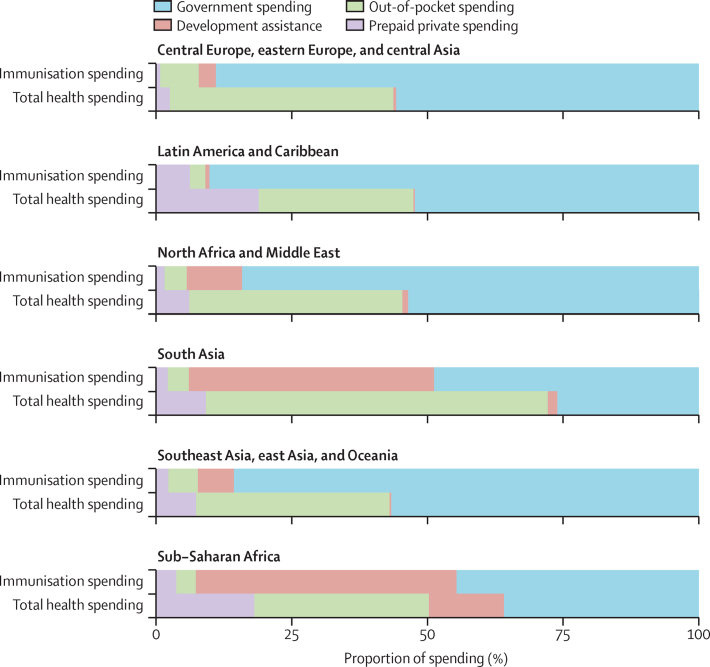
Comparison of total health spending with total spending on immunisation patterns by GBD super-region, 2017 Each set of bars represents a GBD super-region, colour-coded by financing source. GBD=Global Burden of Diseases, Injuries, and Risk Factors Study.

In 2017, the share of spending by source in the health sector as a whole was different from that in the immunisation programme (figure 3). Although government spending was a dominant source of spending across both sectors, out-of-pocket spending was dominant in the health sector but not a major source of spending on immunisation. Similarly, although development assistance was a key source of immunisation spending, it comprised a much smaller proportion of spending in the health sector. These distinctions were even more pronounced between GBD super-regions. For instance, in sub-Saharan Africa, development assistance for immunisation was 48·1% (95% UI 44·7–51·2; $879 million) of total immunisation spending for each region in 2017, whereas in south Asia it was 45·4% (37·2–52·9; $495 million); however, development assistance for health made up 13·8% (12·8–14·8) or $11·4 billion of total health spending in sub-Saharan Africa, and 1·8% (1·4–2·2) or $1·9 billion of total health spending in south Asia.

In 2017, immunisation spending per surviving infant was similar in low-income and lower-middle-income countries, with spending at an average of $40 (95% UI 38–42) in low-income countries and $42 (39–46) in lower-middle-income countries (table 2). In low-income countries, however, development assistance made up the largest share of total immunisation spending (69·4% [64·6–72·0]; $630·2 million), whereas in lower-middle-income and upper-middle-income countries, governments were the main source of spending on immunisation (53·8% [49·5–57·6] or $1·4 billion [1·2–1·6] in lower-middle-income countries, and 91·8% [89·5–92·9] or $4·8 billion [4·4–5·3] in upper-middle-income countries). The rate of growth in immunisation spending was highest in low-income countries, at 11·2% (10·5–11·8) between 2000 and 2017, followed by 7·7% (7·0–8·5) in lower-middle-income countries. Across super-regions, spending per surviving infant was highest in Latin America and the Caribbean in 2017, at $213 (187–243). Across all super-regions except for sub-Saharan Africa and south Asia, governments were the source of almost all immunisation spending. In sub-Saharan Africa and south Asia, government spending and development assistance made up similar proportions of total immunisation spending in 2017. In sub-Saharan Africa, government spending accounted for 44·6% (41·5–48·0) of total immunisation spending, or $818 million (727–936), and development assistance accounted for 48·1% (44·7–51·2) of total immunisation spending, or $879 million; meanwhile, in south Asia, government spending accounted for 48·6% (41·3–55·9) of total immunisation spending, or $536 million (396–727), and development assistance accounted for 45·4% (37·2–52·9) of total immunisation spending, or $495 million. These two regions also experienced the highest growth rates in total immunisation spending between 2000 and 2017, with a growth rate of 10·4% (9·5–11·2) for sub-Saharan Africa and 7·2% (5·9–8·5) for south Asia.

**Table 2:** Spending on immunisation by World Bank income group, GBD super-region, Gavi support status, and in 135 low-income and middle-income countries and territories

		**Health spending by source, 2017**		**Fraction of total immunisation spending, 2017**				**AROC, 2000–17**				
		Total immunisation spending per surviving infant	Income classification	Government fraction	Out-of-pocket fraction	Prepaid private fraction	Development assistance for health fraction	Government AROC	Out-of-pocket AROC	Prepaid private AROC	Development assistance for health AROC	Total immunisation AROC
Global		84 (80 to 90)	..	63·2 (60·6 to 65·0)	3·6 (2·6 to 6·7)	2·8 (2·6 to 3·2)	30·4 (28·6 to 31·9)	5·6 (4·9 to 6·2)	5·1 (3·2 to 6·7)	5·9 (4·5 to 7·2)	12·9	7·0 (6·5 to 7·5)
World Bank income group												
	Upper-middle Income	145 (133 to 160)	..	91·8 (89·5 to 92·9)	3·8 (2·8 to 5·9)	4·2 (3·7 to 4·8)	0·2 (0·2 to 0·2)	5·6 (4·7 to 6·4)	5·6 (2·8 to 7·8)	5·3 (3·7 to 6·8)	−0·9	5·5 (4·7 to 6·4)
	Lower-middle Income	42 (39 to 46)	..	53·8 (49·5 to 57·6)	4·7 (3·0 to 10·8)	2·1 (1·6 to 2·6)	39·4 (35·5 to 42·6)	5·9 (4·9 to 6·9)	3·6 (1·6 to 5·6)	7·7 (5·0 to 10·2)	13·2	7·7 (7·0 to 8·5)
	Low income	40 (38 to 42)	..	24·2 (21·7 to 26·3)	4·6 (2·8 to 10·8)	1·8 (1·3 to 2·6)	69·4 (64·6 to 72·0)	4·2 (3·3 to 5·1)	8·3 (6·3 to 10·4)	12·2 (9·4 to 15·1)	20·6	11·2 (10·5 to 11·8)
GBD super-region												
	Central Europe, eastern Europe, and central Asia	131 (116 to 147)	..	89·0 (85·8 to 91·2)	7·1 (5·0 to 10·4)	0·7 (0·5 to 0·9)	3·1 (2·8 to 3·5)	4·0 (2·8 to 5·0)	8·6 (5·8 to 11·4)	−0·8 (−4·0 to 2·4)	19·5	4·3 (3·3 to 5·3)
	Latin America and Caribbean	213 (187 to 243)	..	90·2 (88·5 to 91·6)	2·9 (1·8 to 4·4)	6·2 (5·2 to 7·4)	0·7 (0·6 to 0·8)	5·4 (4·2 to 6·6)	10·1 (6·2 to 13·8)	7·7 (5·7 to 9·6)	4·0	5·6 (4·4 to 6·8)
	North Africa and Middle East	106 (94 to 121)	..	84·1 (81·3 to 86·2)	4·1 (2·7 to 7·0)	1·5 (1·2 to 1·8)	10·2 (8·9 to 11·5)	5·3 (4·2 to 6·5)	4·7 (1·9 to 7·3)	5·7 (3·1 to 8·4)	26·1	5·9 (4·9 to 7·0)
	South Asia	34 (29 to 41)	..	48·6 (41·3 to 55·9)	3·9 (1·6 to 11·2)	2·1 (1·3 to 3·3)	45·4 (37·2 to 52·9)	5·7 (3·6 to 8·1)	4·9 (1·2 to 9·3)	10·8 (5·4 to 16·1)	9·5	7·2 (5·9 to 8·5)
	Southeast Asia, east Asia, and Oceania	63 (53 to 76)	..	85·7 (79·7 to 88·6)	5·5 (3·3 to 11·7)	2·2 (1·6 to 3·0)	6·6 (5·5 to 7·9)	6·2 (3·8 to 8·1)	2·1 (−0·4 to 4·6)	1·9 (−1·9 to 5·8)	14·0	6·0 (3·9 to 7·8)
	Sub-Saharan Africa	55 (51 to 59)	..	44·6 (41·5 to 48·0)	3·6 (2·4 to 7·8)	3·7 (3·1 to 4·3)	48·1 (44·7 to 51·2)	6·9 (5·9 to 8·0)	6·0 (4·4 to 7·9)	5·9 (4·0 to 7·6)	21·9	10·4 (9·5 to 11·2)
Gavi status												
	Gavi	41 (38 to 45)	..	43·5 (39·6 to 47·3)	4·2 (2·5 to 10·4)	1·9 (1·6 to 2·4)	50·3 (45·9 to 53·4)	6·1 (5·1 to 7·1)	4·5 (2·6 to 6·6)	8·7 (6·1 to 11·2)	15·2	9·0 (8·3 to 9·7)
	Non-Gavi	132 (121 to 144)	..	91·1 (88·7 to 92·2)	4·1 (3·1 to 6·6)	4·1 (3·7 to 4·7)	0·7 (0·6 to 0·7)	5·5 (4·7 to 6·2)	5·5 (2·8 to 7·5)	5·3 (3·8 to 6·8)	5·7	5·5 (4·7 to 6·2)
Unallocable development assistance												
	Unallocable	12 (12 to 12)	..	..	..	..	100·0 (100·0 to 100·0)	..	..	..	11·3	11·3 (11·3 to 11·3)
Central Europe, eastern Europe, and central Asia												
	Albania	97 (75 to 122)	Upper-middle income	95·6 (92·8 to 97·2)	3·1 (1·6 to 6·0)	1·2 (0·6 to 2·1)	0·2 (0·1 to 0·2)	7·9 (5·7 to 10·1)	0·3 (−4·0 to 4·9)	9·5 (2·6 to 16·5)	..	7·5 (5·3 to 9·6)
	Armenia	102 (81 to 128)	Upper-middle income	73·8 (67·1 to 79·5)	3·1 (1·5 to 6·0)	0·3 (0·2 to 0·6)	22·7 (17·9 to 28·1)	9·3 (6·5 to 12·3)	182·7 (36·2 to 592·1)	8·2 (2·4 to 14·8)	..	11·3 (8·8 to 13·9)
	Azerbaijan	42 (33 to 53)	Upper-middle income	72·3 (63·0 to 79·2)	8·0 (3·5 to 17·3)	0·2 (0·1 to 0·3)	19·5 (15·2 to 24·5)	7·9 (5·1 to 10·7)	10·1 (4·5 to 15·5)	11·0 (4·2 to 18·3)	..	9·4 (7·0 to 11·9)
	Belarus	149 (116 to 188)	Upper-middle income	86·6 (79·0 to 92·5)	12·8 (7·0 to 20·4)	0·6 (0·3 to 1·0)	..	2·8 (0·6 to 5·1)	2·3 (−2·9 to 7·4)	−2·7 (−8·0 to 3·2)	..	2·7 (0·7 to 4·8)
	Bosnia and Herzegovina	144 (116 to 178)	Upper-middle income	95·8 (94·2 to 97·1)	2·2 (1·1 to 3·8)	1·1 (0·6 to 2·0)	0·8 (0·7 to 1·0)	5·9 (3·9 to 8·0)	−5·2 (−9·9 to −0·7)	7·7 (1·0 to 14·3)	..	5·2 (3·3 to 7·2)
	Bulgaria	387 (311 to 473)	Upper-middle income	90·8 (83·7 to 95·1)	8·8 (4·5 to 15·8)	0·4 (0·2 to 0·7)	..	4·5 (2·7 to 6·4)	9·0 (3·5 to 14·7)	17·6 (10·6 to 24·4)	..	4·8 (3·1 to 6·6)
	Georgia	125 (96 to 162)	Upper-middle income	77·0 (69·8 to 82·1)	7·3 (4·6 to 13·7)	1·5 (0·8 to 2·4)	14·2 (10·8 to 18·2)	12·0 (9·2 to 15·2)	4·6 (1·9 to 6·9)	48·0 (39·3 to 57·0)	..	12·0 (9·3 to 14·6)
	Kazakhstan	278 (216 to 360)	Upper-middle income	87·2 (79·6 to 92·5)	11·6 (6·2 to 19·2)	1·2 (0·7 to 2·0)	..	9·0 (6·3 to 11·6)	43·3 (34·5 to 53·8)	22·5 (15·0 to 30·2)	..	9·8 (7·1 to 12·3)
	Kyrgyzstan	45 (41 to 50)	Lower-middle income	23·9 (18·2 to 30·4)	6·6 (2·9 to 14·9)	0·0 (0·0 to 0·0)	69·5 (61·6 to 75·9)	2·8 (0·2 to 5·6)	4·9 (−0·4 to 9·5)	27·8 (0·1 to 68·3)	..	10·7 (8·5 to 12·9)
	Mongolia	39 (30 to 51)	Lower-middle income	89·5 (82·1 to 93·3)	5·8 (2·6 to 13·1)	0·7 (0·4 to 1·4)	3·9 (2·9 to 5·0)	6·5 (3·9 to 9·2)	40·5 (30·2 to 52·2)	5·0 (−1·3 to 11·2)	..	7·1 (4·6 to 9·7)
	Montenegro	195 (141 to 270)	Upper-middle income	96·3 (94·6 to 97·4)	2·4 (1·2 to 4·4)	1·3 (0·8 to 2·0)	..	5·9 (2·9 to 8·9)	−1·7 (−6·0 to 3·0)	8·0 (1·5 to 15·0)	..	5·5 (2·7 to 8·4)
	North Macedonia	217 (149 to 306)	Upper-middle income	96·6 (95·3 to 97·6)	1·9 (0·9 to 3·4)	1·1 (0·7 to 1·8)	0·3 (0·2 to 0·4)	5·7 (2·8 to 8·9)	−3·0 (−7·3 to 1·5)	7·3 (1·0 to 14·2)	..	5·4 (2·6 to 8·4)
	Moldova	106 (83 to 136)	Lower-middle income	80·7 (75·8 to 85·3)	2·1 (0·9 to 4·1)	0·3 (0·2 to 0·5)	16·9 (13·0 to 21·1)	3·7 (1·2 to 6·3)	3·8 (−1·7 to 9·6)	5·2 (−0·8 to 12·2)	..	4·8 (2·6 to 7·2)
	Romania	168 (134 to 212)	Upper-middle income	96·0 (93·4 to 97·9)	3·7 (1·9 to 6·3)	0·2 (0·1 to 0·4)	..	4·0 (2·0 to 6·1)	6·1 (0·9 to 11·8)	1·3 (−4·7 to 7·3)	..	4·0 (2·1 to 6·1)
	Russia	168 (136 to 206)	Upper-middle income	92·7 (87·6 to 95·8)	6·7 (3·5 to 11·8)	0·6 (0·4 to 1·0)	..	3·0 (1·2 to 4·7)	9·4 (3·9 to 15·1)	−4·3 (−8·7 to 0·2)	..	3·2 (1·4 to 4·8)
	Serbia	237 (188 to 296)	Upper-middle income	97·9 (96·6 to 98·8)	1·7 (0·9 to 3·0)	0·4 (0·2 to 0·6)	..	6·7 (4·6 to 8·8)	−1·8 (−6·2 to 2·7)	−1·1 (−6·3 to 4·1)	..	6·0 (4·1 to 8·0)
	Tajikistan	31 (29 to 35)	Low income	24·5 (18·2 to 31·5)	3·9 (1·7 to 9·2)	0·1 (0·0 to 0·1)	71·5 (64·1 to 78·2)	13·9 (10·9 to 17·1)	11·8 (6·4 to 18·3)	47·9 (38·7 to 58·0)	..	22·3 (19·5 to 24·9)
	Turkmenistan	73 (56 to 95)	Upper-middle income	91·9 (85·2 to 95·6)	6·8 (3·2 to 13·6)	1·3 (0·8 to 2·0)	..	6·8 (4·3 to 9·2)	40·3 (31·6 to 51·4)	9·2 (3·1 to 15·7)	..	6·8 (4·5 to 9·2)
	Ukraine	91 (69 to 117)	Lower-middle income	93·7 (89·4 to 96·5)	5·7 (2·8 to 10·1)	0·6 (0·3 to 1·1)	..	0·7 (−1·5 to 3·0)	−0·7 (−5·5 to 4·2)	−2·5 (−8·3 to 3·6)	..	0·6 (−1·5 to 2·7)
	Uzbekistan	29 (24 to 37)	Lower-middle income	67·9 (59·2 to 75·0)	6·3 (2·7 to 15·2)	0·2 (0·1 to 0·3)	25·6 (20·0 to 31·0)	5·8 (2·9 to 8·8)	41·4 (29·1 to 54·8)	10·6 (4·2 to 17·4)	23·3	8·1 (5·4 to 10·7)
Latin America and Caribbean												
	Argentina	420 (334 to 526)	Upper-middle income	82·1 (73·3 to 89·0)	15·1 (8·0 to 24·0)	2·9 (2·3 to 3·5)	..	5·1 (2·9 to 7·5)	15·9 (9·5 to 21·4)	3·5 (0·4 to 6·5)	..	5·9 (3·8 to 8·0)
	Belize	89 (71 to 110)	Upper-middle income	97·2 (95·5 to 98·2)	1·2 (0·5 to 2·6)	1·6 (1·0 to 2·4)	..	6·7 (4·7 to 8·7)	3·4 (−1·7 to 8·9)	3·9 (−1·5 to 9·4)	..	6·6 (4·6 to 8·5)
	Bolivia	73 (59 to 91)	Lower-middle income	80·5 (76·7 to 83·9)	1·8 (0·8 to 3·5)	1·0 (0·6 to 1·7)	16·6 (13·2 to 20·2)	6·8 (4·6 to 9·1)	6·3 (1·0 to 11·8)	2·8 (−3·1 to 8·6)	−2·1	4·0 (2·6 to 5·6)
	Brazil	366 (289 to 454)	Upper-middle income	90·1 (87·8 to 92·1)	0·3 (0·1 to 0·5)	9·7 (7·7 to 12·0)	..	4·9 (3·0 to 7·0)	6·3 (−0·2 to 12·8)	8·1 (5·6 to 10·5)	..	5·1 (3·2 to 7·2)
	Colombia	106 (84 to 132)	Upper-middle income	94·1 (92·4 to 95·4)	1·7 (0·8 to 3·3)	4·2 (3·2 to 5·4)	..	5·0 (3·1 to 6·8)	5·7 (−0·1 to 11·5)	7·8 (4·0 to 11·7)	..	5·1 (3·3 to 7·0)
	Costa Rica	222 (180 to 269)	Upper-middle income	93·4 (89·2 to 96·1)	5·3 (2·6 to 9·4)	1·3 (0·9 to 2·0)	..	4·9 (3·1 to 6·6)	8·2 (2·2 to 13·8)	10·6 (5·9 to 15·5)	..	5·1 (3·2 to 6·8)
	Cuba	477 (368 to 619)	Upper-middle income	98·8 (98·3 to 99·1)	0·2 (0·1 to 0·5)	0·5 (0·2 to 0·9)	0·5 (0·4 to 0·6)	7·2 (5·2 to 9·4)	1·2 (−4·3 to 6·8)	7·0 (0·6 to 13·8)	..	7·2 (5·2 to 9·4)
	Dominica	136 (105 to 176)	Upper-middle income	98·8 (97·7 to 99·4)	0·8 (0·3 to 2·0)	0·3 (0·2 to 0·6)	..	2·7 (0·6 to 4·9)	−1·3 (−6·1 to 3·7)	33·3 (25·6 to 42·0)	..	2·7 (0·6 to 4·9)
	Dominican Republic	73 (56 to 92)	Upper-middle income	95·8 (93·6 to 97·2)	1·9 (0·8 to 4·2)	2·3 (1·5 to 3·3)	..	7·4 (5·2 to 9·7)	7·3 (1·4 to 13·5)	7·2 (2·0 to 12·6)	..	7·1 (5·0 to 9·4)
	Ecuador	160 (129 to 198)	Upper-middle income	96·0 (94·2 to 97·2)	2·0 (1·0 to 3·9)	2·0 (1·3 to 2·9)	..	9·5 (7·5 to 11·7)	12·0 (6·0 to 18·1)	11·2 (6·0 to 16·4)	..	9·6 (7·6 to 11·7)
	El Salvador	99 (79 to 124)	Lower-middle income	95·6 (93·0 to 97·2)	2·8 (1·3 to 5·2)	1·7 (1·0 to 2·7)	..	3·4 (1·6 to 5·4)	7·1 (1·2 to 13·2)	7·9 (2·1 to 13·4)	..	3·6 (1·7 to 5·4)
	Grenada	115 (91 to 148)	Upper-middle income	98·0 (96·6 to 98·7)	1·0 (0·4 to 2·4)	1·0 (0·6 to 1·7)	..	3·5 (1·4 to 5·5)	0·8 (−4·5 to 6·2)	4·1 (−1·5 to 10·0)	..	3·4 (1·4 to 5·5)
	Guatemala	76 (57 to 99)	Upper-middle income	88·5 (81·3 to 93·1)	8·9 (4·4 to 16·4)	2·1 (1·2 to 3·3)	0·5 (0·4 to 0·7)	7·7 (5·3 to 10·1)	11·4 (4·6 to 17·8)	14·8 (8·5 to 21·5)	0·0	8·0 (5·7 to 10·1)
	Guyana	153 (125 to 187)	Upper-middle income	82·6 (79·2 to 85·7)	1·2 (0·5 to 2·4)	0·8 (0·4 to 1·3)	15·4 (12·4 to 18·6)	5·0 (2·7 to 7·2)	5·2 (−0·7 to 11·8)	3·7 (−2·2 to 9·8)	..	6·0 (3·9 to 8·1)
	Haiti	30 (30 to 32)	Low income	7·1 (4·9 to 9·9)	1·1 (0·5 to 2·5)	1·3 (0·6 to 2·5)	90·5 (87·3 to 93·2)	1·1 (−2·2 to 4·5)	6·7 (0·4 to 12·8)	12·3 (5·8 to 19·0)	9·6	8·4 (7·7 to 8·9)
	Honduras	51 (39 to 66)	Lower-middle income	93·9 (92·7 to 94·8)	0·8 (0·3 to 1·8)	1·6 (0·9 to 2·6)	3·7 (2·8 to 4·7)	5·4 (3·0 to 8·0)	5·8 (−0·0 to 12·1)	6·3 (−0·0 to 13·1)	4·9	5·4 (3·1 to 7·9)
	Jamaica	104 (79 to 135)	Upper-middle income	94·4 (92·5 to 95·8)	0·9 (0·4 to 2·4)	4·7 (3·4 to 6·3)	..	0·1 (−2·0 to 2·2)	1·0 (−4·1 to 6·4)	1·8 (−2·5 to 6·2)	..	0·2 (−1·9 to 2·3)
	Mexico	131 (106 to 162)	Upper-middle income	95·9 (93·9 to 97·2)	2·3 (1·1 to 4·1)	1·9 (1·2 to 2·8)	..	6·8 (5·1 to 8·7)	1·3 (−3·8 to 6·7)	14·1 (8·7 to 20·0)	..	6·7 (5·0 to 8·6)
	Nicaragua	83 (67 to 103)	Lower-middle income	78·2 (73·5 to 82·4)	1·3 (0·6 to 2·8)	0·5 (0·3 to 0·8)	20·0 (16·0 to 24·4)	5·2 (2·8 to 7·6)	3·1 (−2·1 to 8·9)	3·0 (−2·9 to 9·2)	16·8	6·3 (4·1 to 8·4)
	Paraguay	226 (172 to 293)	Upper-middle income	96·8 (95·5 to 97·7)	0·7 (0·3 to 1·4)	2·6 (1·6 to 3·9)	..	6·4 (4·2 to 8·6)	12·4 (5·6 to 19·8)	5·6 (0·7 to 10·7)	..	6·4 (4·2 to 8·7)
	Peru	170 (132 to 213)	Upper-middle income	95·3 (93·2 to 96·9)	2·5 (1·2 to 4·6)	2·2 (1·4 to 3·2)	..	7·7 (5·6 to 9·6)	12·2 (5·6 to 19·0)	5·9 (0·9 to 11·4)	..	7·7 (5·7 to 9·6)
	Saint Lucia	139 (108 to 177)	Upper-middle income	97·5 (96·1 to 98·3)	0·9 (0·4 to 2·2)	1·6 (1·0 to 2·3)	..	2·5 (0·5 to 4·6)	0·6 (−4·1 to 5·7)	0·8 (−3·4 to 5·8)	..	2·5 (0·4 to 4·5)
	Saint Vincent and the Grenadines	88 (67 to 117)	Upper-middle income	97·7 (95·8 to 98·7)	1·5 (0·6 to 3·5)	0·8 (0·5 to 1·3)	..	2·4 (0·2 to 4·6)	0·7 (−4·3 to 6·2)	4·4 (−1·7 to 10·8)	..	2·4 (0·2 to 4·6)
	Suriname	88 (67 to 115)	Upper-middle income	93·5 (91·0 to 95·1)	1·9 (0·8 to 4·1)	4·6 (3·5 to 6·0)	..	4·9 (2·8 to 7·3)	6·3 (0·1 to 12·1)	3·4 (−0·2 to 7·0)	..	4·9 (2·7 to 7·2)
	Venezuela	39 (29 to 50)	Upper-middle income	88·6 (85·5 to 91·1)	2·1 (0·9 to 4·4)	9·2 (7·1 to 11·8)	..	−1·2 (−3·2 to 0·9)	4·0 (−1·7 to 9·9)	3·7 (0·3 to 7·5)	..	−0·8 (−2·8 to 1·3)
North Africa and Middle East												
	Afghanistan	29 (28 to 32)	Low income	8·1 (5·7 to 10·9)	5·0 (2·1 to 15·1)	0·3 (0·1 to 0·5)	86·6 (77·2 to 90·6)	14·3 (11·1 to 17·4)	9·7 (4·5 to 14·7)	28·8 (19·3 to 38·2)	..	26·6 (20·3 to 30·1)
	Algeria	127 (96 to 167)	Upper-middle income	94·8 (90·2 to 97·5)	4·8 (2·1 to 9·5)	0·4 (0·2 to 0·6)	..	7·8 (5·0 to 10·4)	6·4 (−0·5 to 13·0)	5·0 (−1·1 to 11·8)	..	7·6 (4·8 to 10·2)
	Egypt	42 (29 to 58)	Lower-middle income	87·0 (73·5 to 92·3)	11·1 (5·3 to 25·0)	2·0 (1·1 to 3·2)	..	2·6 (−0·2 to 5·4)	2·2 (−1·2 to 5·8)	10·8 (4·0 to 17·6)	..	2·4 (−0·1 to 4·9)
	Iran	170 (119 to 247)	Upper-middle income	96·3 (93·7 to 97·6)	1·8 (0·7 to 4·4)	1·9 (1·2 to 2·9)	..	8·2 (5·2 to 11·4)	−3·3 (−7·7 to 1·7)	13·2 (7·5 to 19·5)	..	7·6 (4·7 to 10·7)
	Iraq	69 (49 to 93)	Upper-middle income	100·0 (100·0 to 100·0)	0·0 (0·0 to 0·0)	0·0 (0·0 to 0·0)	..	4·3 (1·8 to 7·0)	−32·9 (−83·8 to −0·1)	0·8 (−5·5 to 7·6)	..	4·3 (1·8 to 7·0)
	Jordan	195 (153 to 251)	Upper-middle income	80·5 (73·3 to 86·6)	14·4 (8·3 to 21·8)	4·5 (3·1 to 6·4)	0·6 (0·4 to 0·7)	6·0 (3·9 to 8·4)	5·9 (1·0 to 10·5)	8·9 (4·5 to 13·7)	..	6·1 (4·2 to 8·3)
	Lebanon	247 (176 to 342)	Upper-middle income	93·4 (89·6 to 94·7)	1·0 (0·1 to 4·5)	4·5 (3·8 to 5·2)	1·2 (0·8 to 1·6)	5·0 (2·1 to 8·0)	1·4 (−1·8 to 5·8)	6·9 (3·1 to 10·5)	..	5·1 (2·2 to 8·0)
	Libya	562 (421 to 751)	Upper-middle income	98·9 (97·9 to 99·5)	0·0 (0·0 to 0·0)	1·1 (0·5 to 2·1)	..	3·6 (1·0 to 6·0)	−37·1 (−83·3 to −8·2)	5·7 (−0·5 to 12·3)	..	3·6 (1·1 to 6·0)
	Morocco	76 (55 to 100)	Lower-middle income	90·0 (83·1 to 94·7)	9·2 (4·6 to 16·2)	0·8 (0·5 to 1·3)	..	8·8 (6·0 to 11·7)	13·0 (5·5 to 20·0)	−1·6 (−7·8 to 4·5)	..	8·8 (6·0 to 11·6)
	Palestine	340 (312 to 373)	Lower-middle income	23·3 (18·0 to 29·0)	0·4 (0·2 to 0·9)	4·0 (2·7 to 5·8)	72·2 (65·6 to 78·5)	6·4 (3·6 to 9·4)	−3·0 (−7·7 to 1·8)	9·3 (5·6 to 13·1)	34·4	14·4 (12·4 to 16·4)
	Sudan	31 (30 to 34)	Lower-middle income	10·5 (7·6 to 13·8)	3·0 (0·7 to 11·9)	1·0 (0·5 to 1·8)	85·5 (77·9 to 89·8)	−5·3 (−7·9 to −2·4)	15·7 (8·0 to 26·7)	8·2 (1·7 to 15·3)	..	8·1 (5·9 to 10·4)
	Syria	57 (43 to 78)	Low income	88·1 (83·5 to 91·2)	3·5 (1·4 to 7·4)	1·2 (0·7 to 2·0)	7·3 (5·3 to 9·5)	−0·3 (−2·7 to 2·4)	−4·7 (−9·7 to 0·8)	3·8 (−2·4 to 10·1)	..	−0·1 (−2·3 to 2·4)
	Tunisia	54 (40 to 73)	Lower-middle income	95·4 (86·0 to 97·9)	3·8 (1·2 to 13·4)	0·8 (0·5 to 1·4)	..	3·4 (0·8 to 6·1)	1·6 (−3·2 to 6·2)	3·0 (−3·4 to 9·2)	..	3·3 (0·8 to 5·9)
	Turkey	358 (268 to 470)	Upper-middle income	94·4 (90·3 to 96·8)	4·3 (1·9 to 8·4)	1·3 (0·8 to 2·0)	..	5·5 (3·1 to 8·0)	14·5 (8·1 to 20·9)	1·3 (−4·0 to 7·1)	..	5·6 (3·2 to 8·1)
	Yemen	36 (34 to 39)	Low income	21·5 (16·3 to 27·6)	1·4 (0·3 to 5·4)	0·2 (0·1 to 0·3)	76·9 (70·5 to 82·5)	−5·8 (−8·4 to −3·2)	2·6 (−3·8 to 13·1)	−4·9 (−10·5 to 1·5)	..	3·0 (0·9 to 5·1)
South Asia												
	Bangladesh	51 (46 to 58)	Lower-middle income	31·3 (24·4 to 38·2)	4·0 (1·8 to 9·5)	0·8 (0·4 to 1·5)	63·9 (56·4 to 71·0)	5·3 (2·4 to 8·4)	7·2 (1·8 to 12·8)	11·4 (5·2 to 18·2)	25·4	11·5 (9·6 to 13·6)
	Bhutan	31 (23 to 42)	Lower-middle income	80·9 (70·8 to 87·2)	8·4 (3·6 to 18·7)	0·3 (0·2 to 0·4)	10·5 (7·6 to 13·9)	0·0 (−2·7 to 2·7)	5·0 (0·3 to 10·0)	3·1 (−2·9 to 9·5)	..	1·0 (−1·4 to 3·4)
	India	28 (22 to 38)	Lower-middle income	63·5 (53·7 to 71·4)	4·5 (1·4 to 14·2)	2·7 (1·5 to 4·4)	29·2 (21·5 to 37·6)	7·8 (4·8 to 10·9)	3·9 (−0·9 to 10·0)	11·1 (3·9 to 18·0)	3·8	6·2 (4·3 to 8·2)
	Nepal	42 (36 to 50)	Low income	46·3 (38·3 to 53·6)	3·7 (1·6 to 9·3)	2·8 (1·4 to 4·8)	47·2 (39·1 to 55·6)	−2·2 (−4·8 to 0·5)	6·1 (0·8 to 11·5)	3·6 (−2·5 to 10·4)	..	2·1 (0·0 to 4·2)
	Pakistan	46 (43 to 51)	Lower-middle income	22·5 (17·1 to 28·5)	2·2 (1·0 to 5·7)	1·2 (0·7 to 2·1)	74·0 (67·0 to 79·9)	0·8 (−1·8 to 3·5)	8·4 (3·1 to 14·2)	12·7 (6·2 to 19·5)	25·0	9·4 (7·3 to 11·3)
Southeast Asia, east Asia, and Oceania												
	American Samoa	245 (173 to 348)	Upper-middle income	96·7 (94·0 to 98·0)	2·0 (0·8 to 4·7)	1·3 (0·7 to 2·1)	..	−2·1 (−4·8 to 0·8)	−3·4 (−7·4 to 0·7)	7·5 (1·2 to 14·2)	..	−2·1 (−4·8 to 0·7)
	Cambodia	47 (43 to 52)	Lower-middle income	21·5 (15·9 to 27·6)	5·9 (2·6 to 13·0)	0·2 (0·1 to 0·3)	72·4 (64·9 to 78·3)	3·0 (0·1 to 5·8)	11·7 (6·5 to 16·9)	8·6 (2·1 to 15·4)	24·6	11·5 (9·5 to 13·4)
	China	72 (54 to 94)	Upper-middle income	94·5 (89·1 to 96·9)	3·7 (1·6 to 9·3)	1·7 (1·0 to 2·8)	0·1 (0·0 to 0·1)	6·0 (2·9 to 8·6)	1·9 (−2·7 to 6·5)	−0·1 (−5·3 to 5·2)	−14·6	5·4 (2·5 to 7·9)
	North Korea	45 (42 to 50)	Low income	23·7 (17·8 to 30·0)	2·8 (1·2 to 7·1)	0·5 (0·2 to 0·8)	73·0 (65·8 to 79·4)	−1·0 (−3·6 to 1·9)	1·0 (−4·0 to 6·1)	6·4 (0·5 to 13·0)	..	7·3 (5·4 to 9·3)
	Fiji	211 (158 to 281)	Upper-middle income	90·1 (85·5 to 93·2)	5·8 (2·8 to 10·7)	4·1 (2·8 to 5·8)	..	3·2 (0·2 to 6·1)	9·4 (3·3 to 14·9)	11·0 (4·9 to 17·5)	..	3·6 (0·8 to 6·5)
	Indonesia	49 (38 to 63)	Lower-middle income	63·9 (54·9 to 70·9)	10·2 (5·4 to 19·2)	4·7 (2·9 to 7·3)	21·2 (16·1 to 26·8)	7·0 (4·1 to 10·0)	0·6 (−3·8 to 5·0)	4·1 (−1·4 to 10·2)	19·4	6·8 (4·3 to 9·1)
	Kiribati	177 (158 to 201)	Lower-middle income	41·6 (35·3 to 48·5)	1·3 (0·6 to 2·9)	0·7 (0·4 to 1·2)	56·4 (49·5 to 62·9)	2·5 (0·2 to 4·8)	4·1 (−1·2 to 9·3)	46·0 (37·5 to 54·8)	..	7·7 (6·0 to 9·4)
	Laos	61 (53 to 72)	Lower-middle income	43·9 (35·8 to 52·4)	3·0 (1·3 to 6·8)	0·7 (0·4 to 1·3)	52·4 (43·9 to 60·5)	13·2 (10·0 to 16·3)	4·7 (−0·5 to 9·7)	7·3 (1·2 to 13·8)	..	17·0 (14·5 to 19·5)
	Malaysia	113 (89 to 143)	Upper-middle income	85·7 (77·7 to 90·5)	11·3 (6·4 to 19·3)	3·0 (2·1 to 4·3)	0·0 (0·0 to 0·0)	7·3 (4·9 to 9·5)	2·9 (−1·0 to 7·2)	5·6 (1·0 to 9·9)	..	6·5 (4·5 to 8·5)
	Maldives	176 (125 to 242)	Upper-middle income	96·6 (94·7 to 97·6)	1·1 (0·4 to 2·9)	2·3 (1·7 to 3·0)	..	9·9 (6·7 to 13·0)	2·3 (−2·5 to 7·2)	8·9 (4·7 to 13·3)	..	9·8 (6·6 to 12·7)
	Marshall Islands	109 (81 to 148)	Upper-middle income	92·8 (87·4 to 95·9)	5·8 (2·7 to 11·2)	1·3 (0·8 to 2·1)	..	0·7 (−1·8 to 3·4)	9·2 (3·6 to 14·7)	−1·5 (−6·8 to 4·1)	..	0·9 (−1·5 to 3·6)
	Mauritius	273 (207 to 360)	Upper-middle income	89·4 (83·3 to 93·7)	8·7 (4·4 to 14·9)	1·9 (1·2 to 2·7)	..	7·2 (4·7 to 9·9)	9·7 (3·7 to 15·3)	13·8 (8·1 to 20·1)	..	7·5 (5·0 to 10·0)
	Federated States of Micronesia	61 (44 to 83)	Lower-middle income	89·6 (81·3 to 95·0)	10·4 (5·0 to 18·7)	0·0 (0·0 to 0·0)	..	2·0 (−1·0 to 5·0)	4·8 (−0·9 to 10·8)	0·2 (−42·4 to 65·8)	..	2·2 (−0·6 to 5·0)
	Myanmar	35 (32 to 39)	Lower-middle income	22·3 (16·4 to 29·1)	5·0 (2·1 to 13·8)	0·0 (0·0 to 0·0)	72·7 (64·4 to 79·2)	18·0 (14·5 to 21·4)	2·7 (−2·0 to 7·2)	3·8 (−18·2 to 34·4)	..	20·5 (13·8 to 24·4)
	Papua New Guinea	33 (28 to 38)	Lower-middle income	43·6 (35·6 to 51·7)	2·3 (1·0 to 5·8)	0·0 (0·0 to 0·0)	54·1 (46·1 to 61·9)	2·2 (−0·5 to 4·9)	1·6 (−3·0 to 6·6)	10·3 (−35·7 to 74·8)	..	6·9 (4·8 to 9·2)
	Philippines	33 (24 to 45)	Lower-middle income	82·0 (72·6 to 88·8)	13·7 (6·7 to 23·7)	3·7 (2·2 to 5·7)	0·6 (0·4 to 0·8)	8·3 (5·5 to 11·3)	8·9 (2·8 to 14·8)	9·5 (3·1 to 16·5)	−0·0	8·3 (5·7 to 11·0)
	Samoa	64 (49 to 81)	Upper-middle income	95·9 (90·4 to 98·1)	3·9 (1·7 to 9·3)	0·2 (0·1 to 0·4)	..	4·1 (1·9 to 6·2)	−0·3 (−5·4 to 4·6)	49·0 (40·3 to 58·4)	..	3·9 (1·7 to 5·9)
	Solomon Islands	98 (90 to 108)	Lower-middle income	31·2 (25·5 to 37·7)	1·5 (0·7 to 3·4)	0·0 (0·0 to 0·0)	67·3 (60·8 to 73·0)	1·5 (−1·0 to 4·0)	2·7 (−2·0 to 7·9)	13·9 (−33·2 to 75·3)	..	8·4 (6·5 to 10·4)
	Sri Lanka	34 (26 to 44)	Upper-middle income	65·3 (50·8 to 73·5)	13·2 (5·7 to 31·4)	1·4 (0·8 to 2·4)	20·1 (15·1 to 25·4)	2·4 (−0·2 to 5·1)	4·2 (−0·7 to 10·1)	3·1 (−3·0 to 9·4)	..	4·1 (1·8 to 6·6)
	Thailand	169 (128 to 221)	Upper-middle income	93·6 (89·4 to 95·7)	2·9 (1·3 to 6·7)	3·5 (2·3 to 5·0)	0·0 (0·0 to 0·0)	6·8 (4·4 to 9·4)	−0·0 (−4·3 to 4·4)	8·2 (2·8 to 13·9)	..	6·5 (4·2 to 9·0)
	Timor-Leste	132 (119 to 151)	Lower-middle income	28·3 (21·5 to 36·0)	0·4 (0·2 to 1·3)	1·9 (1·1 to 3·0)	69·4 (60·8 to 76·9)	8·7 (5·6 to 11·8)	24·6 (18·1 to 32·0)	16·0 (9·6 to 22·5)	..	17·0 (14·5 to 19·2)
	Tonga	66 (49 to 89)	Upper-middle income	92·0 (85·1 to 96·0)	6·8 (3·1 to 13·4)	1·2 (0·5 to 2·4)	..	1·8 (−0·8 to 4·5)	5·4 (−0·9 to 11·5)	12·6 (6·1 to 19·0)	..	2·0 (−0·5 to 4·6)
	Vanuatu	35 (25 to 47)	Lower-middle income	93·6 (86·9 to 96·7)	5·4 (2·3 to 12·0)	1·0 (0·6 to 1·7)	..	1·6 (−1·1 to 4·1)	4·0 (−1·3 to 9·6)	26·7 (18·7 to 34·3)	..	1·7 (−0·9 to 4·2)
	Vietnam	28 (22 to 38)	Lower-middle income	56·6 (41·6 to 67·1)	19·1 (9·6 to 39·3)	1·2 (0·7 to 2·1)	23·0 (16·7 to 29·2)	6·2 (3·4 to 9·0)	1·0 (−3·3 to 6·0)	−1·6 (−7·6 to 4·4)	..	5·9 (3·2 to 8·4)
Sub-Saharan Africa												
	Angola	31 (26 to 37)	Lower-middle income	53·3 (46·7 to 59·4)	1·9 (0·7 to 4·7)	4·3 (2·7 to 6·5)	40·5 (33·2 to 48·0)	9·9 (7·0 to 12·6)	10·3 (4·5 to 16·3)	14·5 (9·1 to 20·5)	18·4	12·3 (10·4 to 14·3)
	Benin	28 (25 to 32)	Low income	27·8 (21·3 to 34·9)	2·3 (0·7 to 8·0)	1·3 (0·7 to 2·3)	68·5 (60·1 to 75·6)	10·4 (7·3 to 13·7)	9·2 (2·6 to 16·5)	16·4 (9·8 to 23·7)	27·4	16·9 (14·6 to 19·1)
	Botswana	196 (157 to 239)	Upper-middle income	90·4 (86·7 to 92·9)	4·7 (2·4 to 8·1)	4·9 (3·9 to 6·1)	..	7·8 (5·7 to 9·9)	7·4 (1·8 to 13·2)	5·3 (2·2 to 8·6)	..	7·6 (5·6 to 9·6)
	Burkina Faso	40 (36 to 46)	Low income	29·1 (22·7 to 35·7)	1·6 (0·7 to 3·4)	2·3 (1·1 to 3·8)	67·0 (59·1 to 74·1)	3·9 (1·2 to 6·9)	14·7 (7·9 to 22·2)	15·5 (9·0 to 23·0)	..	11·6 (9·5 to 13·8)
	Burundi	22 (21 to 25)	Low income	12·2 (9·1 to 15·9)	6·3 (2·9 to 14·2)	5·2 (2·8 to 9·0)	76·3 (67·5 to 82·9)	8·2 (4·8 to 11·5)	6·4 (1·3 to 12·0)	16·4 (9·3 to 23·3)	..	18·3 (14·0 to 21·1)
	Cape Verde	113 (80 to 157)	Lower-middle income	98·1 (95·9 to 98·9)	1·2 (0·4 to 3·4)	0·7 (0·4 to 1·1)	..	3·1 (0·2 to 6·0)	2·4 (−2·3 to 7·7)	9·8 (2·6 to 17·2)	..	3·1 (0·2 to 6·1)
	Cameroon	26 (22 to 31)	Lower-middle income	51·7 (42·5 to 60·0)	5·3 (2·2 to 11·6)	0·6 (0·3 to 1·0)	42·5 (35·2 to 50·5)	7·3 (4·1 to 10·0)	9·8 (4·0 to 15·9)	12·9 (5·7 to 20·5)	..	11·0 (8·5 to 13·2)
	Central African Republic	60 (59 to 61)	Low income	3·6 (2·6 to 5·0)	0·8 (0·3 to 2·3)	0·2 (0·1 to 0·4)	95·3 (93·5 to 96·7)	−3·8 (−6·6 to −0·9)	3·6 (−1·5 to 8·9)	15·4 (8·7 to 22·4)	..	16·6 (13·9 to 19·1)
	Chad	16 (15 to 18)	Low income	25·6 (20·0 to 31·6)	0·4 (0·1 to 1·3)	1·6 (0·8 to 3·0)	72·5 (65·7 to 78·5)	6·1 (3·0 to 9·0)	4·7 (−1·3 to 11·1)	19·0 (12·0 to 26·3)	..	14·8 (12·5 to 17·0)
	Comoros	65 (55 to 79)	Lower-middle income	55·5 (47·4 to 63·3)	1·4 (0·5 to 4·2)	0·5 (0·2 to 0·9)	42·5 (34·9 to 50·5)	9·1 (6·1 to 12·3)	3·0 (−1·8 to 8·3)	15·6 (8·0 to 22·9)	..	12·5 (10·1 to 15·1)
	Congo	51 (44 to 61)	Lower-middle income	45·4 (37·2 to 54·2)	7·0 (3·1 to 14·5)	0·8 (0·4 to 1·3)	46·9 (38·8 to 54·4)	7·7 (4·7 to 10·8)	15·3 (9·8 to 21·5)	17·0 (10·1 to 24·2)	..	12·4 (9·9 to 15·0)
	Côte d'Ivoire	74 (66 to 84)	Lower-middle income	23·5 (18·1 to 29·3)	2·2 (0·9 to 5·8)	4·7 (2·5 to 7·6)	69·7 (61·1 to 76·9)	6·3 (3·2 to 9·3)	5·4 (0·6 to 10·4)	13·6 (7·2 to 20·3)	..	14·7 (12·3 to 17·1)
	Democratic Republic of the Congo	42 (40 to 48)	Low income	5·9 (4·1 to 7·8)	7·2 (3·1 to 18·4)	2·1 (1·1 to 3·9)	84·7 (73·9 to 89·7)	13·5 (10·5 to 16·6)	18·7 (13·2 to 24·5)	22·6 (15·1 to 30·3)	26·4	23·5 (22·1 to 24·5)
	Djibouti	77 (70 to 87)	Lower-middle income	33·0 (26·3 to 40·5)	0·5 (0·1 to 2·0)	0·2 (0·1 to 0·3)	66·3 (58·7 to 73·1)	7·6 (4·8 to 10·6)	8·4 (1·3 to 18·0)	16·8 (10·2 to 23·7)	..	14·7 (12·5 to 17·2)
	Equatorial Guinea	29 (21 to 40)	Upper-middle income	87·0 (71·5 to 93·5)	12·2 (5·6 to 27·8)	0·9 (0·5 to 1·7)	..	11·1 (8·1 to 14·0)	12·1 (7·1 to 17·0)	13·8 (6·4 to 21·4)	..	11·2 (8·3 to 14·0)
	Eritrea	28 (27 to 30)	Low income	10·6 (7·7 to 14·0)	6·0 (2·7 to 13·3)	0·6 (0·3 to 1·1)	82·8 (76·0 to 87·3)	3·4 (0·5 to 6·4)	5·4 (0·5 to 11·1)	6·7 (0·4 to 13·4)	31·1	14·9 (12·2 to 17·0)
	Eswatini	133 (102 to 175)	Lower-middle income	83·8 (74·6 to 89·5)	12·0 (6·3 to 21·1)	4·1 (2·8 to 5·9)	..	6·9 (4·3 to 9·5)	11·7 (5·6 to 17·7)	2·5 (−1·9 to 7·3)	..	7·0 (4·7 to 9·4)
	Ethiopia	40 (35 to 48)	Low income	40·5 (33·6 to 46·7)	0·6 (0·3 to 1·5)	5·1 (2·7 to 8·5)	53·8 (44·9 to 62·4)	11·2 (8·2 to 14·3)	2·7 (−3·0 to 8·5)	23·3 (16·4 to 30·4)	26·8	15·9 (13·6 to 18·2)
	Gabon	106 (73 to 150)	Upper-middle income	93·7 (87·8 to 96·1)	3·4 (1·3 to 9·4)	2·9 (1·9 to 4·3)	..	4·2 (1·2 to 7·3)	6·9 (2·4 to 11·4)	6·4 (1·1 to 12·2)	..	4·3 (1·4 to 7·4)
	Gambia	90 (85 to 97)	Low income	17·9 (13·7 to 22·8)	3·2 (1·5 to 7·0)	1·9 (0·9 to 3·5)	77·0 (71·6 to 81·5)	6·2 (3·2 to 9·2)	6·4 (1·7 to 11·3)	8·9 (2·4 to 15·5)	..	16·1 (13·7 to 18·2)
	Ghana	74 (59 to 92)	Lower-middle income	61·9 (53·6 to 68·7)	3·8 (1·6 to 8·2)	2·4 (1·3 to 4·0)	31·8 (25·1 to 39·2)	9·6 (6·3 to 12·7)	1·7 (−3·0 to 6·5)	9·1 (2·6 to 15·8)	16·7	10·3 (7·5 to 12·7)
	Guinea	41 (39 to 44)	Low income	15·2 (11·2 to 19·7)	1·6 (0·6 to 4·0)	2·0 (1·0 to 3·5)	81·2 (75·3 to 86·2)	2·6 (−0·0 to 5·5)	7·8 (2·3 to 13·5)	29·1 (21·6 to 37·5)	39·3	14·2 (12·1 to 16·3)
	Guinea-Bissau	25 (24 to 28)	Low income	18·2 (13·5 to 23·7)	3·5 (1·5 to 8·4)	1·1 (0·5 to 2·1)	77·1 (70·3 to 82·7)	−2·4 (−5·3 to 0·6)	7·5 (2·0 to 13·1)	37·2 (28·8 to 46·0)	30·0	7·5 (4·9 to 9·8)
	Kenya	52 (45 to 62)	Lower-middle income	32·6 (25·5 to 39·8)	7·9 (4·0 to 16·0)	4·3 (2·4 to 6·6)	55·2 (46·2 to 63·2)	6·1 (3·4 to 9·2)	8·1 (3·1 to 13·3)	9·3 (3·5 to 15·4)	15·2	9·9 (8·2 to 11·7)
	Lesotho	44 (40 to 50)	Lower-middle income	31·9 (24·9 to 39·8)	3·0 (1·0 to 8·3)	0·0 (0·0 to 0·0)	65·1 (57·7 to 71·8)	0·1 (−2·5 to 2·9)	6·3 (0·6 to 12·6)	−45·3 (−57·1 to −27·5)	17·7	6·0 (4·2 to 7·8)
	Liberia	94 (88 to 104)	Low income	15·6 (11·7 to 21·0)	7·2 (3·8 to 14·3)	1·4 (0·7 to 2·5)	75·8 (68·7 to 81·2)	9·2 (5·9 to 12·7)	8·4 (4·3 to 12·8)	20·0 (13·2 to 27·5)	19·2	15·0 (13·0 to 16·3)
	Madagascar	26 (24 to 28)	Low income	16·8 (12·4 to 22·0)	6·2 (2·9 to 13·2)	1·9 (1·0 to 3·2)	75·0 (68·6 to 80·6)	4·8 (1·8 to 7·7)	6·9 (1·5 to 12·3)	9·7 (3·6 to 16·1)	19·8	12·5 (10·6 to 14·1)
	Malawi	50 (45 to 56)	Low income	19·8 (14·2 to 26·6)	7·9 (4·2 to 16·2)	1·6 (0·8 to 2·6)	70·6 (62·3 to 77·1)	13·8 (10·5 to 17·3)	16·5 (11·0 to 22·4)	8·0 (1·9 to 14·3)	5·2	6·7 (6·2 to 7·4)
	Mali	55 (46 to 66)	Low income	52·8 (44·6 to 60·8)	1·3 (0·6 to 3·2)	0·6 (0·3 to 1·1)	45·3 (37·4 to 53·4)	5·6 (2·6 to 8·6)	15·4 (8·8 to 21·9)	9·8 (2·7 to 16·8)	18·1	8·8 (6·4 to 11·1)
	Mauritania	114 (101 to 130)	Lower-middle income	37·9 (31·0 to 45·2)	1·3 (0·5 to 3·2)	1·2 (0·6 to 2·0)	59·7 (52·0 to 67·0)	11·7 (8·4 to 14·9)	8·6 (2·4 to 14·9)	19·7 (12·4 to 27·3)	..	17·8 (15·2 to 20·4)
	Mozambique	51 (48 to 54)	Low income	17·0 (13·0 to 22·0)	0·5 (0·2 to 1·2)	0·3 (0·1 to 0·6)	82·2 (77·1 to 86·2)	6·2 (3·3 to 9·2)	10·3 (3·6 to 17·0)	17·7 (10·9 to 24·5)	54·8	17·7 (15·1 to 20·2)
	Namibia	369 (274 to 489)	Upper-middle income	82·9 (80·5 to 84·6)	2·5 (1·2 to 4·5)	14·6 (13·4 to 15·8)	−	3·6 (0·8 to 6·6)	15·1 (8·4 to 21·9)	4·7 (1·6 to 7·7)	..	3·8 (1·2 to 6·8)
	Niger	35 (33 to 37)	Low income	15·4 (11·7 to 20·0)	0·7 (0·3 to 1·7)	0·7 (0·3 to 1·2)	83·2 (78·2 to 87·4)	9·4 (6·7 to 12·3)	6·6 (0·7 to 12·5)	14·6 (8·0 to 21·7)	62·4	21·5 (19·2 to 23·9)
	Nigeria	58 (49 to 69)	Lower-middle income	57·1 (49·5 to 64·0)	1·0 (0·3 to 3·3)	0·3 (0·2 to 0·6)	41·6 (34·5 to 48·8)	7·1 (4·3 to 9·9)	−0·6 (−4·9 to 4·2)	1·3 (−5·1 to 7·8)	45·8	10·2 (7·8 to 12·5)
	Rwanda	66 (62 to 71)	Low income	13·7 (10·0 to 17·9)	3·0 (1·4 to 6·7)	2·5 (1·3 to 4·2)	80·8 (75·1 to 86·0)	8·4 (5·2 to 11·6)	7·1 (1·5 to 12·9)	9·9 (4·3 to 16·1)	20·4	15·6 (14·1 to 16·9)
	São Tomé and Príncipe	545 (503 to 594)	Lower-middle income	27·4 (21·9 to 33·3)	0·5 (0·2 to 1·2)	0·5 (0·2 to 0·8)	71·6 (65·5 to 77·3)	5·0 (2·4 to 7·6)	6·1 (0·7 to 11·5)	16·1 (8·8 to 23·1)	..	13·2 (11·2 to 15·1)
	Senegal	44 (41 to 48)	Lower-middle income	21·0 (15·6 to 26·2)	2·6 (1·1 to 6·5)	2·0 (1·0 to 3·7)	74·4 (68·3 to 79·9)	3·8 (0·8 to 6·5)	11·6 (5·6 to 17·8)	15·7 (9·1 to 22·7)	50·1	13·4 (11·2 to 15·6)
	Sierra Leone	46 (42 to 52)	Low income	25·6 (19·3 to 32·8)	3·0 (1·2 to 8·1)	1·9 (1·0 to 3·5)	69·6 (61·5 to 76·7)	3·5 (0·5 to 6·6)	8·0 (2·7 to 13·9)	31·0 (23·3 to 39·5)	..	11·9 (9·7 to 14·1)
	Somalia	11 (11 to 12)	Low income	8·9 (6·4 to 11·9)	3·5 (1·3 to 9·9)	0·1 (0·1 to 0·2)	87·5 (80·7 to 91·0)	5·7 (2·7 to 8·8)	5·4 (0·2 to 10·6)	14·9 (7·8 to 22·5)	..	19·5 (16·4 to 22·1)
	South Africa	282 (214 to 362)	Upper-middle income	82·8 (80·0 to 84·8)	3·8 (2·1 to 6·6)	12·6 (11·4 to 13·8)	0·8 (0·6 to 1·0)	6·9 (4·6 to 9·2)	2·4 (−2·3 to 7·3)	3·9 (1·3 to 6·5)	..	6·3 (4·1 to 8·5)
	South Sudan	50 (49 to 52)	Low income	4·5 (3·2 to 6·3)	1·3 (0·4 to 4·3)	0·8 (0·4 to 1·4)	93·4 (90·3 to 95·3)	0·2 (−2·8 to 3·3)	37·4 (30·0 to 45·8)	9·9 (3·6 to 16·4)	..	20·0 (17·3 to 22·8)
	Togo	75 (71 to 80)	Low income	14·5 (10·8 to 18·4)	2·6 (1·2 to 5·7)	1·9 (1·0 to 3·2)	81·0 (75·1 to 85·6)	10·1 (7·0 to 13·1)	8·8 (3·2 to 14·3)	21·2 (14·4 to 28·6)	..	21·8 (19·2 to 24·1)
	Uganda	36 (32 to 48)	Low income	16·6 (11·4 to 21·8)	18·4 (10·6 to 40·1)	0·9 (0·4 to 1·6)	64·1 (47·2 to 72·1)	7·4 (4·4 to 10·3)	8·1 (5·3 to 11·3)	−1·7 (−7·1 to 4·0)	12·1	9·8 (8·4 to 10·9)
	Tanzania	45 (40 to 53)	Low income	31·0 (24·1 to 39·1)	9·0 (4·5 to 18·3)	0·2 (0·1 to 0·4)	59·8 (51·0 to 66·9)	5·6 (2·6 to 8·5)	8·0 (3·0 to 13·0)	2·6 (−3·3 to 8·5)	13·9	9·4 (7·8 to 11·0)
	Zambia	58 (49 to 70)	Lower-middle income	52·8 (44·9 to 60·4)	1·1 (0·5 to 2·4)	1·6 (0·9 to 2·6)	44·5 (36·5 to 52·6)	4·4 (1·6 to 7·3)	4·0 (−1·1 to 9·5)	0·0 (−5·7 to 5·6)	51·1	7·8 (5·5 to 10·2)
	Zimbabwe	57 (53 to 62)	Lower-middle income	21·2 (16·1 to 26·8)	0·4 (0·1 to 1·0)	3·5 (1·9 to 5·7)	74·9 (68·4 to 80·8)	4·4 (1·3 to 7·4)	−1·2 (−7·1 to 4·8)	11·7 (5·7 to 17·9)	..	13·8 (11·4 to 16·2)

Estimates are reported with 95% uncertainty intervals (UIs). 95% UIs were not produced for annualised rate of change in development assistance for health. Total immunisation spending is reported in inflation-adjusted 2019 US dollars per surviving infant. The surviving infant population is calculated using livebirths and infant mortality data from GBD 2019. The reported GAVI category covers 72 countries that received GAVI support in our study. AROC=annualised rate of change. GBD=Global Burden of Diseases, Injuries, and Risk Factors Study.

**Figure 4:**
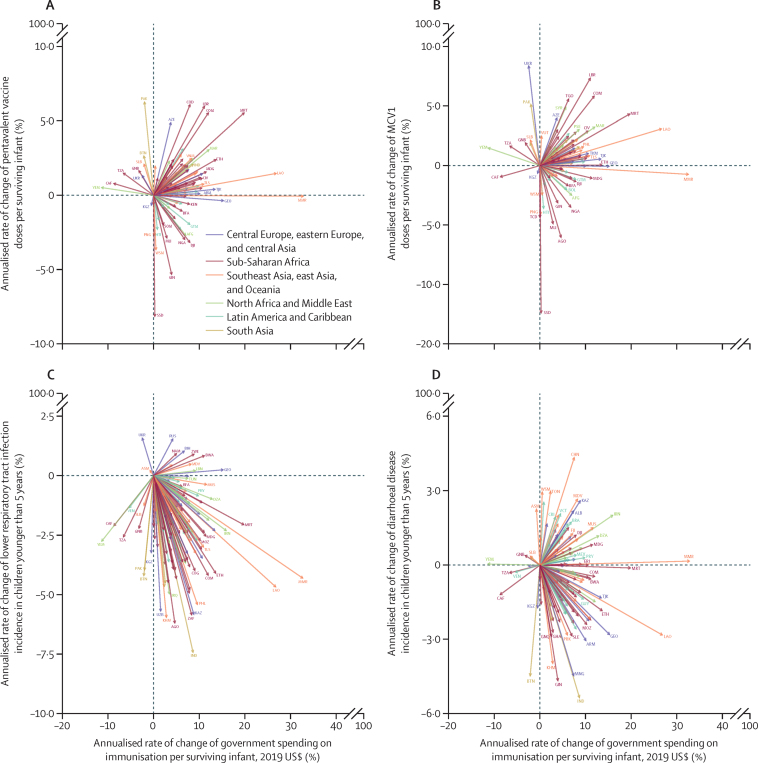
Annualised rates of change of pentavalent vaccine (A) and MCV1 (B) doses administered per surviving infant and government spending on immunisation per surviving infant, and annualised rates of change in the incidence of lower respiratory tract infection (C) and the incidence of diarrhoeal disease (D) in children younger than 5 years and government spending on immunisation per surviving infant Of the 135 low-income and middle-income countries, 123 increased government spending per surviving infant between 2010 and 2017. Each arrow represents one country moving from 2010 to 2017. The start year for South Sudan is 2011. 92 low-income and middle-income countries and territories are represented in panels A and B. All 135 low-income and middle-income countries and territories included in the study are represented in panels C and D. Spending estimates are presented in inflation-adjusted 2019 US dollars. Colours represent GBD super-regions. The surviving infant population is calculated using livebirths and infant mortality data from GBD 2019. Disease incidence data are from GBD 2017. AFG=Afghanistan. AGO=Angola. ALB=Albania. ARM=Armenia. ASM=American Samoa. AZE=Azerbaijan. BFA=Burkina Faso. BIH=Bosnia and Herzegovina. BOL=Bolivia. BRA=Brazil. BTN=Bhutan. BWA=Botswana. CAF=Central African Republic. CHN=China. CIV=Côte d’Ivoire. COD=Democratic Republic of the Congo. COG=Congo (Brazzaville). COM=Comoros. DJI=Djibouti. DZA=Algeria. ERI=Eritrea. ETH=Ethiopia. FJI=Fiji. GBD=Global Burden of Diseases, Injuries, and Risk Factors Study. GEO=Georgia. GHA=Ghana. GIN=Guinea. GNB=Guinea-Bissau. GNQ=Equatorial Guinea. GTM=Guatemala. GUY=Guyana. HTI=Haiti. IND=India. IRN=Iran. IRQ=Iraq. KAZ=Kazakhstan. KEN=Kenya. KGZ=Kyrgyzstan. KHM=Cambodia. LAO=Laos. LBN=Lebanon. LBR=Liberia. MAR=Morocco. MCV1=measles-containing vaccine, first dose. MDG=Madagascar. MDV=Maldives. MEX=Mexico. MLI=Mali. MMR=Myanmar. MNG=Mongolia. MOZ=Mozambique. MRT=Mauritania. MUS=Mauritius. NAM=Namibia. NGA=Nigeria. PAK=Pakistan. PHL=Philippines. PNG=Papua New Guinea. PRK=North Korea. PRY=Paraguay. PSE=Palestine. RUS=Russia. SLB=Solomon Islands. SLE=Sierra Leone. SSD=South Sudan. SOM=Somalia. SYR=Syria. TCD=Chad. TGO=Togo. TJK=Tajikistan. TKM=Turkmenistan. TLS=Timor-Leste. TON=Tonga. TUN=Tunisia. TZA=Tanzania. UKR=Ukraine. UZB=Uzbekistan. VCT=Saint Vincent and the Grenadines. VEN=Venezuela. VNM=Vietnam. VUT=Vanuatu. WSM=Samoa. YEM=Yemen. ZAF=South Africa. ZWE=Zimbabwe.

Higher vaccine coverage corresponded with increased government spending for pentavalent vaccines in 56 (60·9%) countries and for measles vaccines in 54 (58·7%) countries, out of the 92 countries for which dose volume data were available (figure 4). However, there was an inverse relationship in 27 (29·3%) countries for pentavalent vaccines and in 29 (31·5%) countries for measles vaccines, where vaccine coverage decreased while government spending increased; these countries were predominantly in Latin America and the Caribbean and in sub-Saharan Africa. 115 (85·2%) of the 135 low-income and middle-income countries experienced reductions in the incidence of lower respiratory tract infection alongside increases in government spending for all immunisations. There was less concordance between government spending and incidence of diarrhoea, with 70 (51·9%) of the 135 countries documenting reductions in incidence with increased spending, and 54 (40·0%) documenting increased incidence with increased spending. Similar analyses showing annualised changes associated with immunisation spending are shown in the appendix (pp 28–29). 132 of the 135 low-income and middle-income countries had increased total immunisation spending from 2000 to 2017.

## Discussion

The 2011–20 period marked the decade of vaccines, with the GVAP and collaborations that included Gavi, the Bill & Melinda Gates Foundation, UNICEF, WHO, and countless government and non-governmental organisations attempting to deliver vaccines. During this decade, tremendous gains were made. Globally, coverage for key vaccines increased (eg, from 10·7% to 48·5% for PCV3, and from 78·9% to 83·6% for DTP3).^23^ During this same period, immunisation spending in low-income and middle-income countries increased, with the majority of the increase coming from domestic governments. Of the 135 low-income and middle-income countries, 124 increased government spending per surviving infant between 2010 and 2017. During this period, donor assistance also increased, from $1·7 billion to $3·1 billion, even as several countries, including Bhutan and Albania, transitioned away from Gavi support. Also of note are the instances where vaccine coverage decreased while spending increased (in Samoa, Honduras, and El Salvador). This unexpected inverse relationship might have occurred as a result of an unknown mix of several factors, including increasing prices, an increasing population, increasing inefficiencies, and differences in the types of vaccines that countries purchase. Additional research is needed to identify the specific drivers of this unique relationship.

Despite the end of the decade of vaccines, attention towards global financing for vaccines is reaching an unforeseen peak as pharmaceutical companies worldwide race to develop, test, produce, and deliver effective COVID-19 vaccines.^28^ In 2020, global donors committed $2·4 billion to Gavi to help supply and deliver COVID-19 vaccines in low-income and middle-income countries,^29^ although most experts agree this level of support is far from what is needed.^29^ Ongoing prioritisation of funding for immunisations is needed to ensure coverage of vaccines included in traditional essential packages, such as measles, measles-rubella, meningitis, and pneumococcal vaccines, as well as ensure access to vaccines for novel diseases such as SARS-CoV-2. As global investment in R&D for better and more effective vaccines continues, ensuring that all countries have the funds needed to access these powerful tools is essential.

Beyond COVID-19, a few other important developments could influence the availability of funds for immunisation in low-income and middle-income countries. Sustained economic growth in countries that currently receive funding implies a pending transition out of donor support and an increased reliance on domestic funding for immunisation. Although these transitions are needed to ensure that scarce resources are targeted appropriately to those countries that are unable to afford these vaccines, countries are sometimes transitioned out of donor support when government spending on health has not yet expanded to cover all needed services, which might lead to increased out-of-pocket spending.^30^ Although Gavi has put systems in place to manage these transitions, this remains an area that requires close monitoring to limit any setbacks in levels of immunisation coverage in affected countries.

Increasing funding for immunisation is not the only way to increase vaccine coverage. Reductions in the price of vaccines and preferential purchasing arrangements, and reductions in delivery costs, are key ways to improve efficiency. As competition for scarce resources exists in all countries, it is also important to explore gains in efficiency as a means to increase immunisation coverage even as budgets remain stagnant.

As global commitment towards immunisation grows,^31^ lessons from previous policies, outstanding needs, and opportunities must be considered in subsequent strategies from both national and donor perspectives. Cost estimates to achieve the GVAP objective based on both planned introductions and scale-up of routine and new vaccines over 10 years totalled $50–60 billion for 94 low-income and lower-middle income countries.^12^ These funds were tied to multiple goals, including averting childhood deaths due to vaccine-preventable diseases, improving coverage for pentavalent and pneumococcal vaccines, supporting introduction of other new vaccines, and meeting polio elimination targets between 2011 and 2020. Although comprehensive spending estimates for immunisations are not available yet for 2018–20 because of reporting lags, our estimates for 72 countries that received GAVI support in our study suggest it is very unlikely the GVAP spending targets (of $50–60 billion for 10 years) will have been met. These gaps are likely to have led to lower immunisation coverage, and higher preventable mortality in these countries. As the immunisation community turns its attention to the operationalisation of the IA2030 strategy, it is clear that more resources are needed for immunisations, and additional efforts needed to increase efficiency of procurement and delivery of vaccines.

This study has several limitations. First, to generate a comprehensive picture of immunisation spending, we used a wide range of data sources that were not initially designed to be compared. Although we standardised these inputs and they generally aligned in terms of the reported items, the purposes for which they were collected differed. Overall, these differences affected their geographical scope, completeness, and accuracy. Additionally, some of our data, such as the volume and delivery data, were based on modelled estimates. Although our study approximated uncertainty at every stage (except for the tracking of development assistance for health), our estimates remain the outputs in some models. Although these estimates can be valuable for benchmarking and evaluating change over time, more effort is needed to build underlying databases of standardised data that can be used for this type of research. Second, our approach for estimating out-of-pocket spending was especially challenging because of the sparse amount of reported input data available on household spending on immunisation. We therefore leveraged pricing and utilisation data from surveys and private for-profit facilities, but were forced to make many assumptions and take averages across regions and globally when necessary. This approach is similar to what has been implemented in other peer-reviewed disease-specific modelling studies, although the input data on immunisations for private spending were particularly sparse.^32^, ^33^ Although we had a few data sources on immunisation care-seeking preferences, most of our utilisation data were based on the Demographic and Health Surveys, where we used care-seeking practices for diarrhoea for children younger than 5 years as a proxy support for immunisation. We used available spending data from private for-profit facilities for out-of-pocket estimation, and in addition to being scarce, these data might not fully reflect the level of heterogeneity present in costing immunisation services for the different countries, such as differences in user fee structures or existing government support for immunisation services delivered through the private sector. We generated a scalar guided by existing literature on immunisation-seeking practices and costing together with modelling methods to correct for this limitation, but acknowledge that there might be residual areas for improvements in terms of the quality and precision of our estimates. Moreover, limited availability of data for out-of-pocket spending led us to include spending estimates for only ten key immunisations: human papillomavirus, inactivated poliovirus, Japanese encephalitis, measles, measles-rubella, meningococcal group A, pneumococcal, rotavirus, pentavalent, and yellow fever vaccines. Ideally, with the availability of additional data, we would expand this analysis to all vaccine spending. To the degree that households spend on immunisations for other diseases, our out-of-pocket spending estimates should be considered a lower bound, as, if anything, our estimates are biased downward. Third, we had the least data for prepaid private spending, and consider these estimates first-order approximations. However, estimating these first-order approximations (and using them to estimate total spending on immunisations) is an important first step in estimating the total amount of immunisation spending inclusive of all financing sources. Fourth, we only included immunisation spending on R&D from international donors. However, we do not believe we are missing a large set of resources because of this limitation. Out-of-pocket spending, for example, is unlikely to contribute any money towards R&D directly. To the degree that governments of low-income and middle-income countries are funding R&D for immunisations, our estimates should be considered a lower bound, as our estimates would be biased downward. Fifth, because of lags in data reporting for some of our key input data sources, the latest year of data for which estimates were generated was 2017. Although this dataset is still valuable because it is comprehensive, we acknowledge that additional and more recent years of data would have been ideal, especially given the substantial impact of the COVID-19 pandemic. Further standardisation between data sources, to allow for comparability across time and level of granularity—by antigen, for example—would be necessary to provide additional insights into spending trends and priorities. Finally, immunisation spending varies considerably across countries, and any aggregation (eg, by region) eliminates the important nuances in country-level estimates.

In conclusion, our analysis of immunisation spending shows the important role that governments have and continue to play in supporting their national programmes, both supporting the cost of vaccines and the delivery of services. As countries continue to grapple with the COVID-19 response and its economic consequences, it will be important for governments to continue to provide financing for essential health services such as immunisation. Furthermore, countries and the global community have committed to the new IA2030 goals for the next decade, including to reach at least 50% of currently unimmunised children and ensure the sustainability of immunisation programmes worldwide. This will require collective effort to ensure continued progress.

## Data sharing

Data used for this study were extracted from publicly available sources that are listed in the appendix. Further details will be made available on the Global Health Data Exchange website.

## Declaration of interests

We declare no competing interests.
