# Supplementary material for: Estimating total spending by source of funding on routine and supplementary immunisation activities in low-income and middle-income countries, 2000–17: a financial modelling study
Source: Lancet. 2021 Nov 20;398(10314):1875–93. doi: 10.1016/S0140-6736(21)01591-9 (PMC8844562; doi:10.1016/S0140-6736(21)01591-9)
Supplement: Supplementary appendix [file mmc1.pdf]

THE LANCET

Supplementary appendix

This appendix formed part of the original submission and has been peer reviewed. We post it as supplied by the authors.

Supplement to: Ikilezi G, Micah AE, Bachmeier SD, et al. Estimating total spending by source of funding on routine and supplementary immunisation activities in low-income and middle-income countries, 2000–17: a financial modelling study. *Lancet* 2021; published online Nov 4. http://dx.doi.org/10.1016/S0140-6736(21)01591-9.

Estimating immunisation spending to assess trends and gaps in immunisation financing: a financial modelling study

Methods Annex

Institute for Health Metrics and Evaluation

June 2021

Table of Contents

Section 1. Introduction

Section 2. Estimating government spending on immunisation

- S2.1 Data Extraction
- S2.2 Data standardisation and processing
- S2.3 Covariates
- S2.4 Sensitivity Analysis

Section 3. Estimating out-of-pocket spending on immunisation

- S3.1 Volume
- S3.2 Price
- S3.3 Delivery costs
- S3.4 Out-of-pocket scalar

Section 4. Estimating development assistance spending on immunisation

- S4.1 Program Areas
- S4.2 Keyword Search
- S4.3 Methods

Section 5. Estimating prepaid private spending on immunisation

Section 6. Aggregating total spending on immunisation

Section 7. Currency conversion

Section 8. Additional results

Section 9. GATHER Compliance

Section 10. References

Section 1. Introduction

There is limited supply of credible, comprehensive, and comparable data on immunisation spending. While data such as the Joint Reporting Forms, National Health Accounts, country-specific comprehensive multiyear plans provide a snapshot of the financing landscape for immunisation, each of these data sources also have known scope, data quality, and reporting issues that have constrained their credibility and makes them difficult to compare across countries and time, and ultimately prevent them from alone describing the international immunisation financing landscape. To date, no single initiative has used data from all these diverse sources to maximize strengths and compensate for various limitations to generate a comprehensive picture of the immunisation financing landscape.

Comparable immunisation spending estimates are valuable for several reasons. First, they are essential for understanding the current immunisation financing landscape as it relates to overall health spending. Second, country spending on immunisation is an important monitoring indicator for global stakeholders. Specifically, the Global Vaccine Action Plan (GVAP) monitors immunisation spending per child as a measure of government commitment and Gavi's strategy monitors progress against expenditures per child to determine its outcomes on financial sustainability and decision-making about eligibility for transition from external support. Third, it is vital in shedding light on opportunities that may exist for increased resource mobilization. Fourth, it will enable stakeholders to contextualize immunisation spending considering current global health goals. Lastly, it is an essential input for future planning of immunisation activities. With the push for universal health coverage on the global health agenda, measuring what is being spent on immunisation efforts in low- and middle-income countries, in aggregate and within each individual country, will supply valuable information for assessing where opportunities may exist for realignment.

The Gates Foundation funded this study to address this gap in data availability for policy decision making in the global health financing landscape.

Objective of the study

This study generated estimates of immunisation spending in low- and middle-income countries, including both donor funding and domestic spending, disaggregated by funding source (government, donor, household), by activity (routine or supplementary) and by component (vaccine and operational expenses) for all low- and middle-income countries from 2000 through 2017.

Section 2. Estimating government spending on immunisation

S2.1 Data Extraction

Extracted data were standardized using the following criteria. We limited our analysis to recurrent and capital spending, excluding any shared costs. We extracted country, spending year, currency, and currency year for all data.

Data source	Variable extracted	Government immunisation components	Time period of available data	Country years of data	Data points	Currency
WHO National Health Accounts						
- System of Health Accounts (2011)	- HC.6.2 Immunisation programmes	- Total	2000-2016	27	27	Varies by country
- Global Health Expenditure Database (GHED)	- Domestic general government expenditure on immunisation programmes	- Total	2015-2017	93	93	Current NCU
Comprehensive Multi-Year Plans (cMYP)	- Routine recurrent and capital costs<br>- Campaign costs<br>- Vaccine and injection supplies costs<br>- Operational costs	- Total<br>- Routine<br>- Supplementary<br>- Vaccine<br>- Delivery	2004-2017	140	543	Current USD
Financial Sustainability Plans (FSP)	- Routine recurrent and capital costs<br>- Campaign costs<br>- Vaccine and injection supplies costs<br>- Operational costs	- Total<br>- Routine<br>- Supplementary<br>- Vaccine<br>- Delivery	2000-2004	99	398	Current USD
Gavi Historical Co-Financing and Self-Financing	- Country co-financing payments<br>- Country self-financing estimates	- Vaccine	2008-2017	608	608	Current USD

WHO/UNICEF Joint Reporting Forms (JRF)	- Government spending on routine immunisation (Indicator 6540)<br>- Government spending on vaccines (Indicator 6510)	- Routine<br>- Vaccine	2006-2017	1444	2642	Current USD
Immunisation Delivery Cost Catalogue	- Cost per capita without vaccine<br>- Cost per dose without vaccine<br>- Cost per person in the target population without vaccine<br>- Cost per FIC without vaccine	Delivery	2007-2017	17	17	2016 USD

Table 1. Extracted data for government spending on immunisations

**Source**	**Immunisation component or activity**	**Country data availability**	**Country year data availability**
WHO National Health Accounts & Global Health Expenditure Database (GHED)	Total	37.0%	4.9%
Comprehensive Multi-Year Plans (cMYPs) & Financial Sustainability Plans (FSPs)	Total	48.9%	7.0%
	Routine immunisation	52.6%	8.9%
	Supplementary immunisation	45.9%	6.9%
	Vaccines	49.6%	6.0%
	Delivery	48.9%	7.0%
Gavi Historical Co-financing and Self-financing	Vaccines	52.6%	25.0%
WHO/UNICEF Joint Reporting Forms (JRF)	Routine immunisation	94.8%	49.7%
	Vaccines	97.8%	57.6%
Immunisation Delivery Cost Catalogue (IDCC)	Delivery	9.6%	0.7%

Table 2. Data availability by data source

WHO National Health Accounts

We extracted data from the 2011 System of Health Accounts documents. Reports spanning two years (one fiscal year) were extracted using the ending year as the spending year. Few reports included government spending on vaccines. Wherever explicit government spending on HC.6.2 immunisation programs was identified, data were extracted directly and coded as total government spending on immunisation programs. Due to the lack of financing source by health care function data tables, explicit government spending on health functions (HC.6.2 Immunisations programmes) data were uncommon.

Global Health Expenditure Database

We extracted domestic general government expenditure on immunisation programmes from the WHO Global Health Expenditure Database (https://apps.who.int/nha/database/Select/Indicators/en). These data were coded as total government spending on immunisation programs.

Comprehensive Multi-Year Plans & Financial Sustainability Plans

We extracted data from available comprehensive multi-year plans (cMYP) and financial sustainability plans (FSP). We extracted data only from baseline/past expenditure years, excluding forecasted estimates. We extracted government, sub-national government, and government co-financing of Gavi vaccines spending data. We extracted only secured funding, excluding unfunded spending. We excluded shared health systems costs.

Routine recurrent costs and routine capital costs were aggregated and coded as government spending on routine immunisation. Campaign costs were coded as government spending on supplementary immunisation. Routine recurrent vaccine and injection supplies spending and campaign vaccine and injection supplies spending were aggregated and coded as government spending on vaccines. All non-vaccine spending were aggregated and coded as government spending on delivery.

Gavi Historical Co-financing and Self-financing

We extracted data sent from Gavi for country co-financing and country self-financing. If country years overlapped between co-financing and self-financing, data were aggregated. Data were coded as government spending on vaccines.

WHO/UNICEF Joint Reporting Forms

We extracted data for two indicators from the joint reporting forms: indicators 6540 and 6510. Indicator 6540 was coded as government spending on routine immunisation. Indicator 6510 was coded as government spending on vaccines.

Immunisation Delivery Cost Catalogue

We estimated data following the same method as estimating out-of-pocket spending on delivery data. However, the only data used from this method were of country years utilized in the linear mixed effects model. Data were coded as government spending on delivery.

S2.2 Data standardisation and processing

All data were standardised and processed using the following method. Implausible data were flagged and removed from standardization and processing. All data were disaggregated into one of five immunisation components: total government spending on immunisation, government spending on routine immunisation, government spending on supplementary immunisation, government spending on vaccines, or government spending on delivery. All data were currency converted into 2019 USD. We used the following formula with IHME government health expenditure spending estimates to convert all data into proportions of government health expenditure spending by country year.

$$proportion = \frac{extracted\ data\ point}{total\ government\ health\ expenditure\ spending}$$

JRF government spending on vaccines data do not include spending on supplementary/campaign vaccines. We leveraged the cMYP and FSP data to standardize these data to represent the entire government spending on vaccines envelope. We used the following formula with the cMYP and FSP data to calculate proportions of vaccine spending that were supplementary vaccines.

$$proportion = \frac{spending\ on\ supplementary\ vaccines}{total\ spending\ on\ vaccines}$$

We ran a general linear model using GBD super regions and GDP pc to estimate proportions of vaccine spending that were supplementary vaccines.

$$proportion \sim log(GDP\ pc) + GBD\ super\ region$$

The proportions were used to scale up the JRF government spending on vaccines data to represent the full government spending on vaccines envelope.

Next, all data were converted into logit space for the remainder of the data processing, covariate analyses, and modeling. Covariate analyses were performed and a linear mixed effects model was selected for each of the five immunisation components. The covariate analyses are expanded on in the following section.

Gavi co-financing and self-financing government spending on vaccines data do not include spending on all nine of the vaccines we identified as of interest in our study. We leveraged the JRF government spending on vaccines data and the selected linear mixed effects model to standardize these data. We added a binary Gavi co-financing and self-financing indicator variable to the selected linear mixed effects model. We ran the model and subtracted the coefficient of the indicator variable from the Gavi co-financing and self-financing data. We used

this method to scale up these data to represent the full government spending on vaccines envelope.

We used cook’s distance with a standard cutoff of 4 to identify outlier data points in each of the five immunisation component datasets. We removed identified outlier data points from the modeling process. A small number of unreasonable outlier data points and data points that were relatively extreme were manually added or removed from the modeling process, respectively.

We calculated a variance across the entire dataset to use for the modeling process. This is a common approach for tabulated data. We multiplied the variance by a factor of two for data from IDCC, cMYP, and FSP to account for differences in data strength and quality. The data were modeled individually for the five immunisation components referenced above.

Modeled estimates in logit space were transformed into proportions. We used the following formula to transform the proportions of government health expenditure spending into expenditures.

$$expenditure = proportion * government\ health\ expenditure\ spending$$

We calculated the total government spending on immunisation envelope by leveraging all the modeled estimates. We used the following formula to calculate the total government spending on immunisation envelope at the country year level.

$$envelope\ 1 = total\ government\ spending\ on\ immunisation\ estimate$$

$$envelope\ 2 = (government\ spending\ on\ routine\ immunisation\ estimate + government\ spending\ on\ supplemetary\ immunisation\ estimate)$$

$$envelope\ 3 = (government\ spending\ on\ vaccines\ estimate + government\ spending\ on\ delivery\ estimate)$$

$$new\ total\ government\ spending\ on\ immunisation\ estimate = \frac{(envelope\ 1) + (envelope\ 2) + (envelope\ 3)}{3}$$

Government spending on routine immunisation, supplementary immunisation, vaccines, and delivery were then raked according to their original proportion of the total. An example calculation is provided below.

$$new\ government\ spending\ on\ vaccines\ estimate = new\ total\ government\ spending\ on\ immunisation\ estimate * \frac{government\ spending\ on\ vaccines\ estimate}{envelope\ 3}$$

Scatterplots comparing the three envelopes are included below. The axes represent the percent of government health spending that is immunisation according to our three envelopes. Each point represents one country-year. The color of the points represents Global Burden of Disease super regions. The Pearson’s correlation coefficient for each comparison is included.

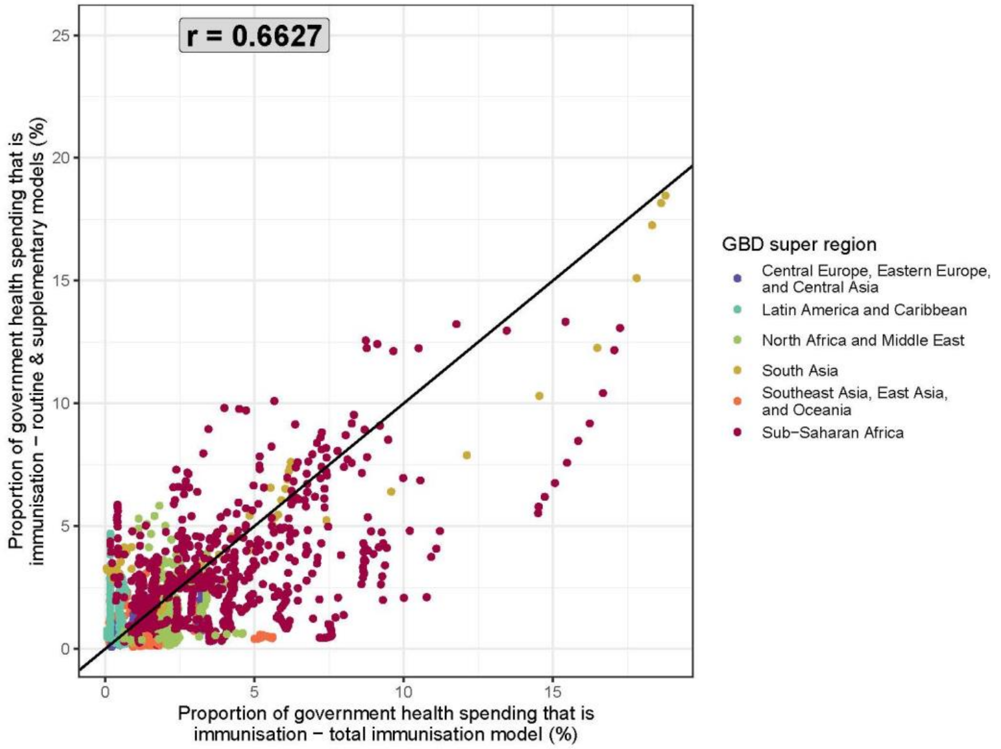
r = 0.6627
Proportion of government health spending that is immunisation − routine & supplementary models (%)
Proportion of government health spending that is immunisation − total immunisation model (%)
GBD super region
Central Europe, Eastern Europe, and Central Asia
Latin America and Caribbean
North Africa and Middle East
South Asia
Southeast Asia, East Asia, and Oceania
Sub−Saharan Africa

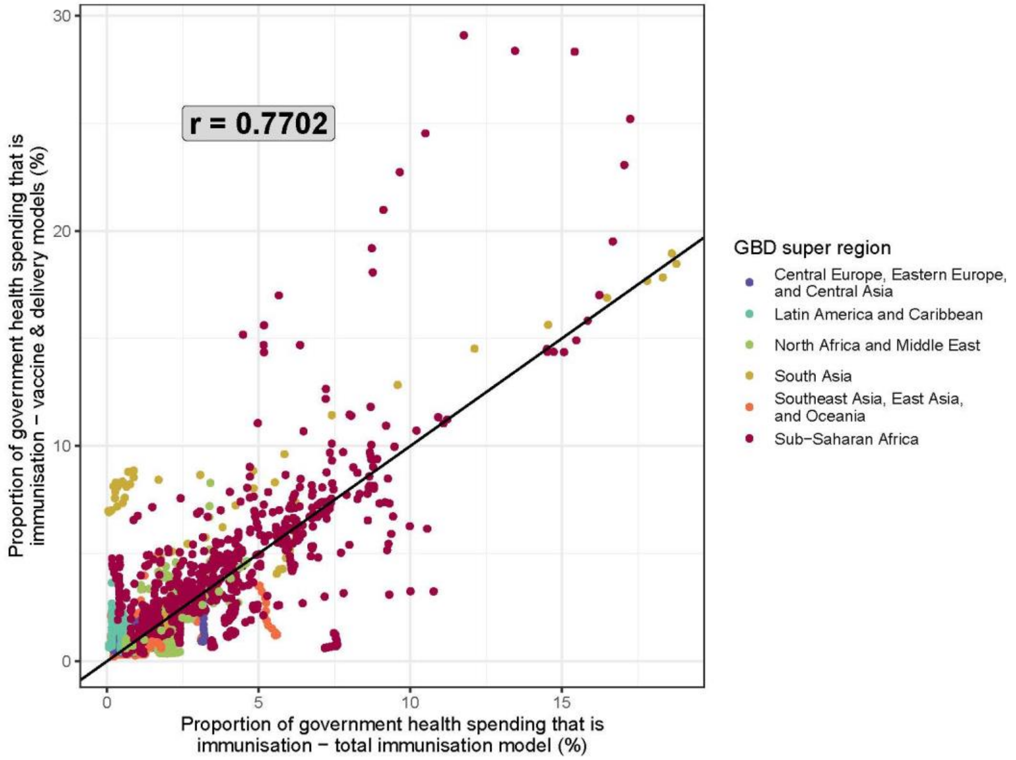
r = 0.7702
Proportion of government health spending that is immunisation − vaccine & delivery models (%)
Proportion of government health spending that is immunisation − total immunisation model (%)
GBD super region
Central Europe, Eastern Europe, and Central Asia
Latin America and Caribbean
North Africa and Middle East
South Asia
Southeast Asia, East Asia, and Oceania
Sub−Saharan Africa

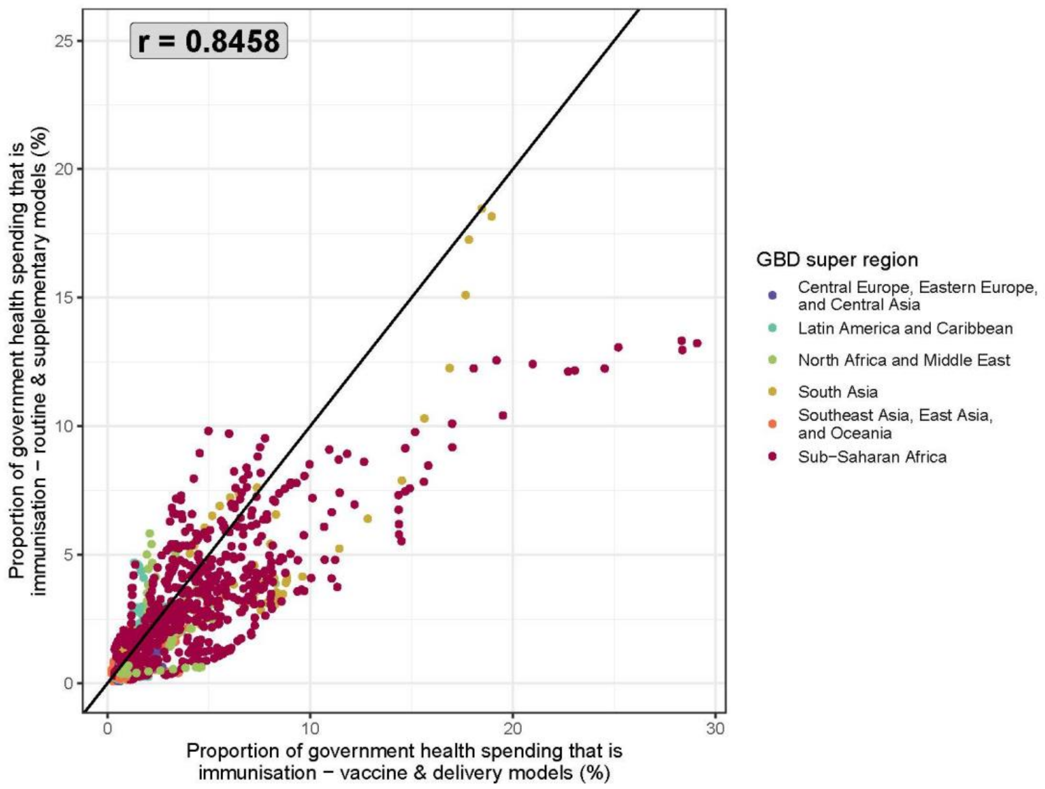

Figure 1. Scatterplot comparisons of total government spending on immunisation envelopes

S2.3 Covariates

Based on a literature review and input from researchers and collaborators, covariates were identified. We selected covariates and a subsequent linear mixed effects model for each of the five immunisation components: total government spending on immunisation, government spending on routine immunisation, government spending on supplementary immunisation, government spending on vaccines, and government spending on delivery. Government health expenditure spending per capita is from Financing Global Health 2019 data. All other covariates are from the Global Burden of Disease 2019 study data. The list of covariates considered for modeling is included below.

- DTP3 vaccine coverage
- MCV1 coverage
- Infant mortality rate
- Surviving infant population (live births * (1 – infant mortality rate))
- Maternal education per capita (years)
- IHME health access and quality index
- Government health expenditure spending per capita

All potential models were considered for selection. An AIC and BIC were calculated for each linear mixed effects model. The models with the best AIC/BIC were chosen to be run with holdouts and a corresponding out of sample root mean square error (RMSE) was calculated. We selected the model with the lowest RMSE for statistical modelling (Spatio-temporal Gaussian process regression). The covariates for each immunisation component are specified.

**Immunisation component**	**Covariates**
Total government spending on immunisation	Surviving infant population
Government spending on routine immunisation	DTP3 vaccine coverage, maternal education per capita (years), IHME health access and quality index, government health expenditure spending per capita
Government spending on supplementary immunisation	MCV1 coverage, surviving infant population, IHME health access and quality index
Government spending on vaccines	Infant mortality rate, surviving infant population, maternal education per capita (years), IHME health access and quality index, government health expenditure spending per capita
Government spending on delivery	Government health expenditure spending per capita

Table 3. Covariates leveraged in government spending on immunisation modelling

Regression output and parameter estimates

Vaccines

```
Linear mixed model fit by REML ['lmerMod']
Formula: y ~ maternal_educ_yrs_pc + logit_haqi + logit_cv_infant_mortality +
    log_cv_surviving_infant_pop_thousands + log_cv_ghes_pc +
    (1 | location_name) + (1 | region_name) + (1 | super_region_name)
   Data: loop_input
REML criterion at convergence: 6276.271
Random effects:
 Groups            Name        Std.Dev.
 location_name     (Intercept) 0.7277
 region_name       (Intercept) 0.6261
 super_region_name (Intercept) 0.5358
 Residual                      0.9663
Number of obs: 2143, groups:  location_name, 132; region_name, 17; super_region_name, 7
Fixed Effects:
                          (Intercept)                   maternal_educ_yrs_pc                             logit_haqi
                             -7.00118                                0.07358                                0.42459
            logit_cv_infant_mortality  log_cv_surviving_infant_pop_thousands                         log_cv_ghes_pc
                             -0.74811                                0.12180                               -0.57680
```

Routine

```
Linear mixed model fit by REML ['lmerMod']
Formula: y ~ maternal_educ_yrs_pc + logit_DTP3_coverage_prop + logit_haqi +
    log_cv_ghes_pc + (1 | location_name) + (1 | region_name) +      (1 | super_region_name)
   Data: loop_input
REML criterion at convergence: 2884.746
Random effects:
 Groups            Name        Std.Dev.
 location_name     (Intercept) 0.5714
 region_name       (Intercept) 0.3301
 super_region_name (Intercept) 0.3395
 Residual                      0.5927
Number of obs: 1420, groups:  location_name, 129; region_name, 17; super_region_name, 7
Fixed Effects:
             (Intercept)      maternal_educ_yrs_pc  logit_DTP3_coverage_prop                logit_haqi
                -2.61650                   0.02097                  -0.06493                   0.41666
          log_cv_ghes_pc
                -0.52051
```

Delivery

```
Linear mixed model fit by REML ['lmerMod']
Formula: y ~ log_cv_ghes_pc + (1 | location_name) + (1 | region_name) +      (1 | super_region_name)
   Data: loop_input
REML criterion at convergence: 508.2958
Random effects:
 Groups            Name        Std.Dev.
 location_name     (Intercept) 0.8116
 region_name       (Intercept) 0.3302
 super_region_name (Intercept) 0.0252
 Residual                      0.7320
Number of obs: 185, groups:  location_name, 66; region_name, 12; super_region_name, 5
Fixed Effects:
   (Intercept)  log_cv_ghes_pc
       -3.2797         -0.4091
```

Supplementary

```
Linear mixed model fit by REML ['lmerMod']
Formula: y ~ logit_haqi + logit_measles_vacc_cov_prop + log_cv_surviving_infant_pop_thousands +
    (1 | location_name) + (1 | region_name) + (1 | super_region_name)
   Data: loop_input
REML criterion at convergence: 519.1381
Random effects:
 Groups            Name        Std.Dev.
 location_name     (Intercept) 0.9685
 region_name       (Intercept) 0.4725
 super_region_name (Intercept) 0.0000
 Residual                      0.8620
Number of obs: 167, groups:  location_name, 62; region_name, 12; super_region_name, 6
Fixed Effects:
                          (Intercept)                             logit_haqi            logit_measles_vacc_cov_prop
                             -8.81575                               -0.05349                               -0.14321
log_cv_surviving_infant_pop_thousands
                              0.31047
```

Total government spending on immunizations

```
Linear mixed model fit by REML ['lmerMod']
Formula: y ~ log_cv_surviving_infant_pop_thousands + (1 | location_name) +
    (1 | region_name) + (1 | super_region_name)
   Data: input

REML criterion at convergence: 895.4

Scaled residuals:
    Min      1Q  Median      3Q     Max
-5.4442 -0.4042  0.0370  0.4106  3.5767

Random effects:
 Groups            Name        Variance Std.Dev.
 location_name     (Intercept) 0.3434   0.5860
 region_name       (Intercept) 0.1941   0.4406
 super_region_name (Intercept) 0.3165   0.5626
 Residual                      0.9475   0.9734
Number of obs: 292, groups:  location_name, 80; region_name, 15; super_region_name, 6

Fixed effects:
                                      Estimate Std. Error t value
(Intercept)                           -4.39765    0.44671  -9.845
log_cv_surviving_infant_pop_thousands -0.01075    0.06080  -0.177

Correlation of Fixed Effects:
            (Intr)
lg_cv_sr___ -0.756
```

S2.4 Sensitivity Analysis

We performed an additional analysis of the government spending components to test the robustness of our data and our methods of identifying outliered data points. We included the outliered data points from the previous analysis. We have included three figures of the following analysis. Because the prepaid private spending estimates are dependent on the government spending estimates, these were also updated for the analysis and production of these figures.

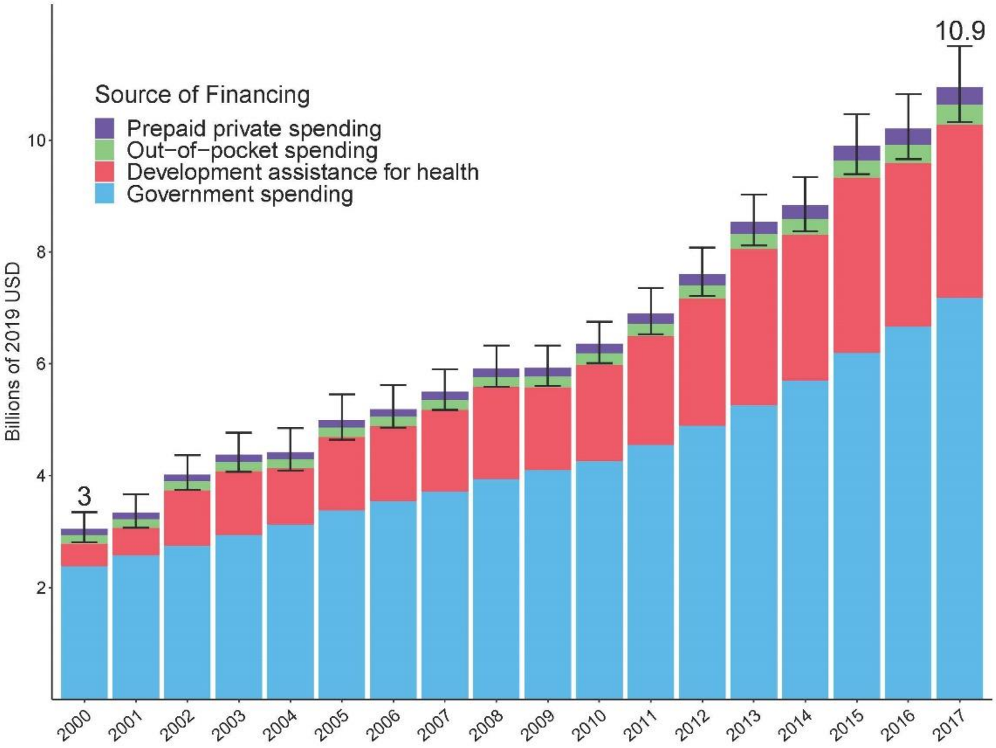

Figure 2. Total spending on immunisation by financing source

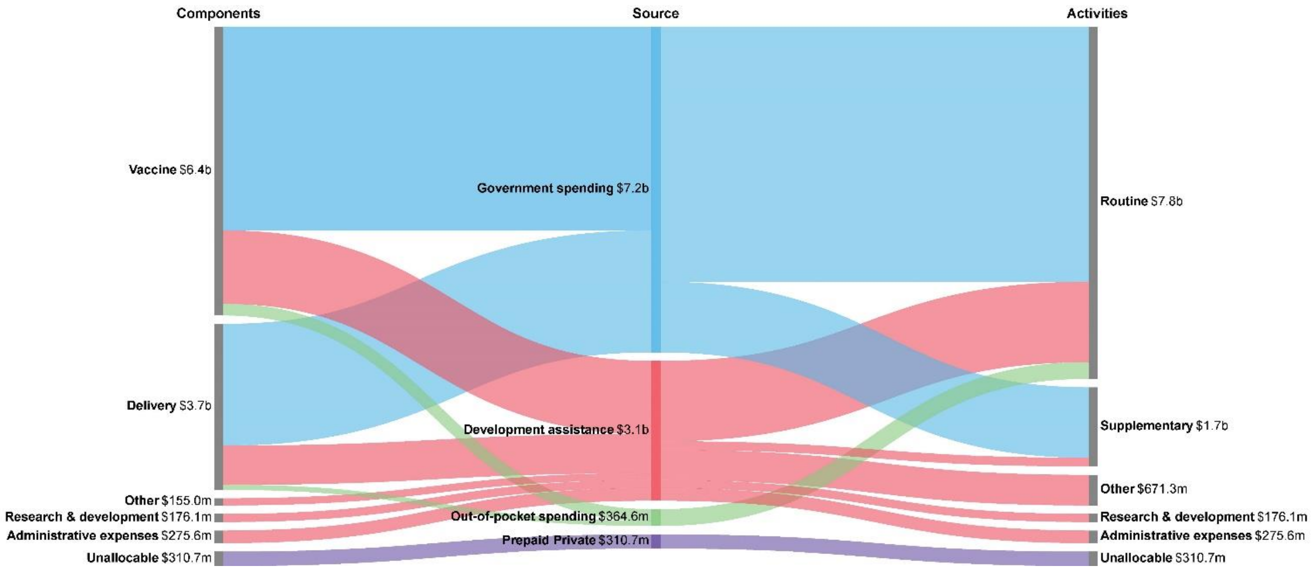

Figure 3. Flows of spending and funding from financing source to immunisation activities and components

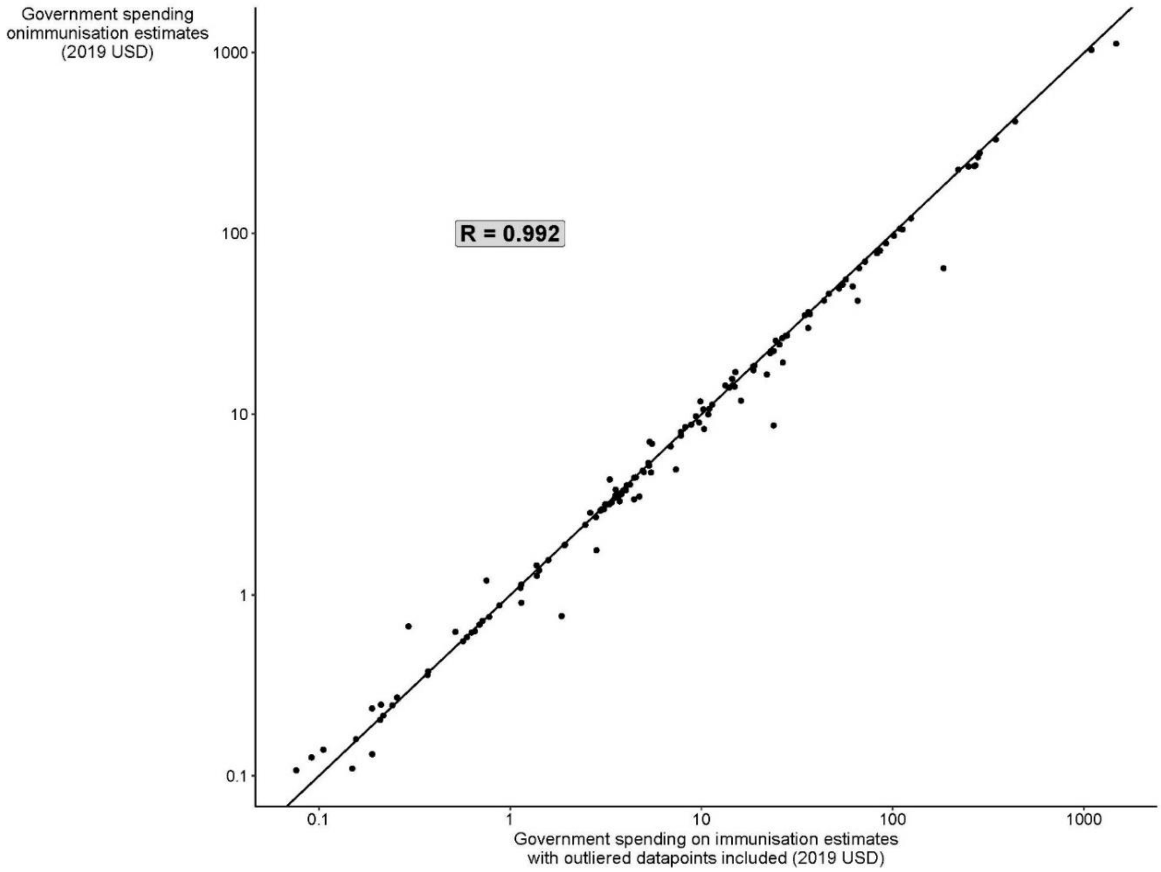

Figure 4. Scatterplot comparisons of total government spending on immunisation envelopes by location, 2017

Section 3. Estimating out-of-pocket spending on immunisation

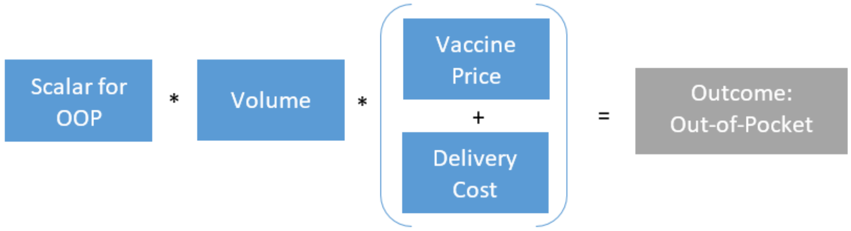

We estimated out-of-pocket spending on immunisations for 135 countries from 2000 to 2017 using a price-volume approach. We chose to limit the immunisations covered in this analysis to ten of the most common ones: Inactivated polio (IPV), Human Papillomavirus (HPV), Japanese Encephalitis (JE), Meningococcal A (MenA), Measles (Measles), Measles-Rubella (MR), Pentavalent (Penta), Pneumococcal (PCV), Rotavirus (RVV), and Yellow Fever (YF).

S3.1 Volume

For each of the vaccines, we utilized existing datasets for vaccine-specific volumes, prices, and delivery costs. For volume, by which we mean the number of vaccines bought by a particular country, we used a dataset from the Decade of Vaccine Economics (DOVE) which reported estimated country-year specific volumes of vaccine purchases, for both routine and supplementary (SIA) vaccine programs. We assume that out-of-pocket spending only occurs for routine immunisations, and so only included the estimated routine values from this dataset. This dataset provided data on 94 countries, 93 of which were within our list of 135 low-and-middle-income countries, for the years 2000-2018, for all 10 vaccines of interest.

Since we did not have reported volumes for every country-year we were estimating for, we used a spatiotemporal Gaussian process regression (St-GPR) approach to estimate the volume of vaccines for all locations. This process, described elsewhere**, leverages relationships between time, space, and covariates to produce estimates for all country-years. We ran a separate St-GPR model for each of the 10 vaccines, using internally produced covariates on vaccine-specific* coverage and universal health coverage to help inform the regressions. Additionally, we assumed vaccine-specific volumes were zero for any country in a region where no vaccine volumes were reported in the DOVE database for the entire time period.

**Universal health coverage (for HPV, JE, and YF), and Vaccine-specific coverage proportions for: Polio, PCV, Rotavirus, Measles, Meningococcal A, and DTP3 (for the Pentavalent model)*
** Stanaway, J. D., et al. (2018). Global, regional, and national comparative risk assessment of 84 behavioural, environmental and occupational, and metabolic risks or clusters of risks for 195 countries and territories, 1990–2017: A systematic analysis for

the Global Burden of Disease Study 2017. Supplementary Appendix 1, Section 2.3.3. *The Lancet*, *392*(10159), 1923–1994. https://doi.org/10.1016/S0140-6736(18)32225-6

S3.2 Price

For price estimates, we used a dataset produced by the Market Information for Access to Vaccines. This reported 1109 data points on the 10 vaccines for 2005-2016 (missing 2009), for all of the countries we were interested in. Because of the relative sparsity of the data, and the desire to leverage the relationship between prices for different vaccines, we elected to use a linear model rather than St-GPR for this component of our model. We therefore produced estimates of vaccine unit price (excluding delivery costs) for each vaccine, and 6 GBD super regions which the 135 LMICs are categorized into. Where reported prices exist for a particular vaccine-country, we use those prices, and otherwise fill missing values with this super-region specific estimate.

Formula: log(PricePerDoseInUSD) ~ vaccine + super_region_name

S3.3 Delivery costs

In addition to the price of a unit of vaccine, there is also the cost of the delivery of the vaccine to consider. While we investigated ways to directly approximate the delivery cost to the consumer, in the end we decided to infer a relationship between the delivery cost countries pay to receive vaccines and the final price paid by consumers. Too little is known about the different component costs charged to consumers at the point of immunisation. The Immunisation Delivery Cost Catalogue is a compilation of many studies on the costs of delivering vaccines. The cost catalogue reports a broad set of estimates which are not directly comparable; for this analysis, we selected only data points which met all of the following criteria: delivery-only costs for one of vaccines of interest, single-vaccine delivery costs (not multi-vaccine), and fiscal/financial costs (not economic costs). For the vaccines we were interested in, there were 54 costs reported for 15 countries. We then adjusted reported per-person and per-FIC costs to per-dose costs to further standardize them. Because of the limited data, we only estimated non-country-specific vaccine-specific delivery costs using a linear mixed effects regression, with random effects by super-region.

Formula: log(Cost) ~ vaccine + (1|super_region_name)

S3.4 Out-of-pocket scalar

With these datasets, we are able to multiply estimated volumes by price and delivery cost to estimate the total envelope of vaccine spending, a portion of which is

out-of-pocket spending. To compute this out-of-pocket portion, we need to estimate a proxy for out-of-pocket spending. We investigated reported data in many SHA reports, but very few of them reported any information on out-of-pocket spending on immunisations. Instead, we decided to estimate the frequency with which people used private healthcare clinics for immunisations. We reviewed literature for estimates of the proportion of immunisations which take place in private facilities at the country-level, and used these estimates as a proxy for out-of-pocket spending. We only included data for for-profit facilities (under the assumption that immunisations at public facilites do not incur out-of-pocket expenses), and estimates which were relatively representative of the country of interest (not exclusively urban). We identified 34 data points in 28 countries which met these criteria.

To estimate a full time-series for these estimates, we needed additional information about how private facility use has changed over time. DHS surveys report the frequency with which parents take their children to private facilities for specific health reasons. Since diarrhea is a common childhood illness which parents would likely go to their typical clinic for, we elected to use that frequency for our time series. With this data, we are only assuming that the time trend of private facility use follows a similar pattern for both diarrhea and immunisations. Rather than using the reported diarrhea private facility utilization rate directly, we adjusted that data using a super-region adjustment factor from the existing immunisation private facility data, bringing the DHS data in line with the immunisation-specific data.

We then ran a St-GPR model for out-of-pocket spending, using GDP per capita as a predictive covariate. By multiplying the 'total expenditure' estimates by this scalar, we finally estimate the amount of out-of-pocket spending in each country from 2000-2018.

Formula: data ~ GDP_per_capita + (1|level_1/level_2/level_3);
Hyperparameters:
data_transform=logit;
gpr_amp_factor=2, 2x amplification scalar;
gpr_scale=5, 5-year temporal correlation;
st_lambda=0.3/0.05, medium-to-high temporal smoothing;
st_zeta=0.001, minimum spatial smoothing.

We tested using other covariates in the ST-GPR model, including LDI per capita, universal health coverage, HAQI (Healthcare Access and Quality Index), and fraction of OOP out of total health expenditure (from FGH 2019). GDP per capita was the best covariate based on visual inspection and goodness of fit.

Private immunisation facility utilization data – literature

**Citation**	**Countries**
Dayan, G. H., Orellana, L. C., Forlenza, R., Ellis, A., Chaui, J., Kaplan, S., & Strebel, P. (2004). Vaccination coverage among children aged 13 to	Argentina

59 months in Buenos Aires, Argentina, 2002. *Revista Panamericana de Salud Pública*, *16*(3). https://doi.org/10.1590/S1020-49892004000900002	
Levin, A., Munthali, S., Vodungbo, V., Rukhadze, N., Maitra, K., Ashagari, T., & Brenzel, L. (2019). Scope and magnitude of private sector financing and provision of immunisation in Benin, Malawi and Georgia. *Vaccine*, *37*(27), 3568–3575. https://doi.org/10.1016/j.vaccine.2019.05.023	Benin, Malawi, Georgia
Dayan, G. H., Orellana, L. C., Forlenza, R., Ellis, A., Chaui, J., Kaplan, S., & Strebel, P. (2004). Vaccination coverage among children aged 13 to 59 months in Buenos Aires, Argentina, 2002. *Revista Panamericana de Salud Pública*, *16*(3). https://doi.org/10.1590/S1020-49892004000900002	Bangladesh, Brazil, Costa Rica, Ethiopia, Honduras, India, Sri Lanka, Morocco, Mauritania, Nicaragua, Pakistan, El Salvador, Thailand, Zimbabwe
Private Sector Engagement in Immunisation: Findings and recommendations from the Middle East and North Africa Region, Final Report. April 2020. *Unicef*.<br>AND<br>Kaddar, Miloud. July 2, 2019. Effective Engagement of the Private Sector: For universal immunisation coverage. *LNCT*.	Egypt, Iraq, Jordan, Lebanon, Libya, Morocco, Sudan, Tunisia, Yemen
Mitrovich, R., Marti, M., Watkins, M., Duclos, P. (2017). A Review of the Private Sector's Contribution to Immunisation Service Delivery in Low, Middle, and High-Income Countries. *WHO (unpublished).* *https://www.who.int/immunisation/sage/meetings/2017/april/2_Review_private_sector_engagement_Mitrovich_et_al.pdf?ua*	India, Lebanon, Mexico, Pakistan, Philippines, Romania

Table 4.

Section 4. Estimating development assistance spending on immunisation

The pipeline to complete the work is shown below.

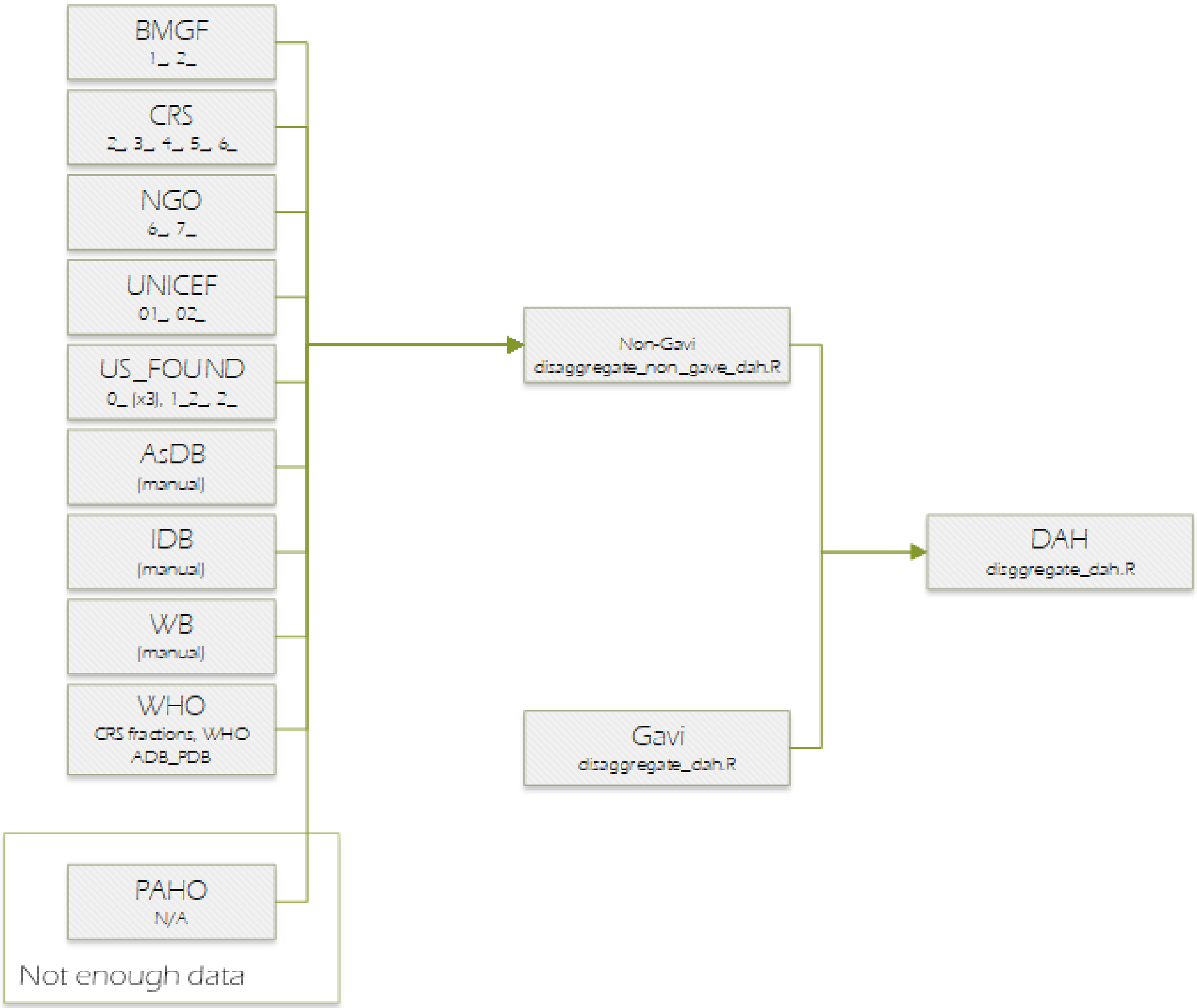

Figure 5. ImmFin DAH pipeline

S4.1 Program areas

IHME currently disaggregates the “newborn and child health” (nch) health focus area into various program areas, one of which is “vaccines” (nch_cnv). This project aims to further disaggregate the nch_cnv program area into the following buckets:

- Programmatic – routine/supplementary
  - Routine (nch_cnv_rout)
  - Supplementary (nch_cnv_supp)
  - Other routine/supplementary (nch_cnv_rs_other)
- Programmatic – vaccine/delivery
  - Vaccine (nch_cnv_comm) – note that “comm” is for commodity (the original term used and the one used in code)
  - Delivery (nch_cnv_deli)
  - Other vaccine/delivery (nch_cnv_dc_other)
- Non-programmatic – R&D (nch_cnv_rd)

It must be noted that the routine/supplementary and vaccine/delivery buckets are simultaneous and mutually exclusive. That is, aside from R&D, a given disbursement must be classified completely as routine, supplementary, and other routine/supplementary *as well as* vaccine, delivery, and other vaccine/delivery.

The figure below shows the project program area hierarchy.

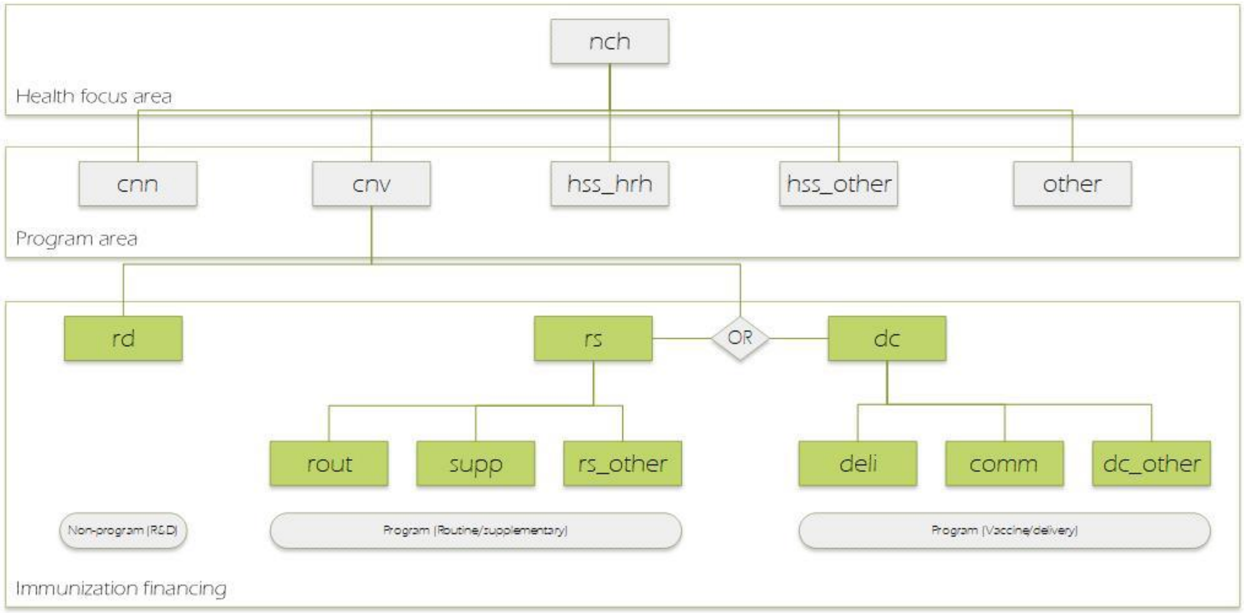

Figure 6.1ImmFin program area hierarchy structure

In a departure from the current DAH hierarchy, the sum of all ImmFin program areas does *not* equal nch_cnv. The OR gate in the above diagram visualizes this – the total cnv spending is the sum of the non-programmatic R&D and either routine/supplementary or vaccine/commodity. Said another way, the total routine/supplementary spending is definitively equal to the total vaccine/commodity spending.

S4.2 Keyword search

A significant component of the ImmFin project consists of modifying the existing DAH keyword search approach to account for the new nch_cnv disaggregation. ImFinance only includes keywords in English, Spanish, and French.

Approach

The general approach to calculating ImmFin spending for channels with project-level descriptions is the same as used for DAH – a keyword search is run across project descriptions and each disbursement is distributed into the relevant buckets based on keyword count ratios. Search strings are provided for the ImmFin program areas as well as for non-programmatic R&D. Any projects that do not get tagged with at least one program area are tagged as “other” within that given program bucket.

A basic disaggregation workflow is shown below.

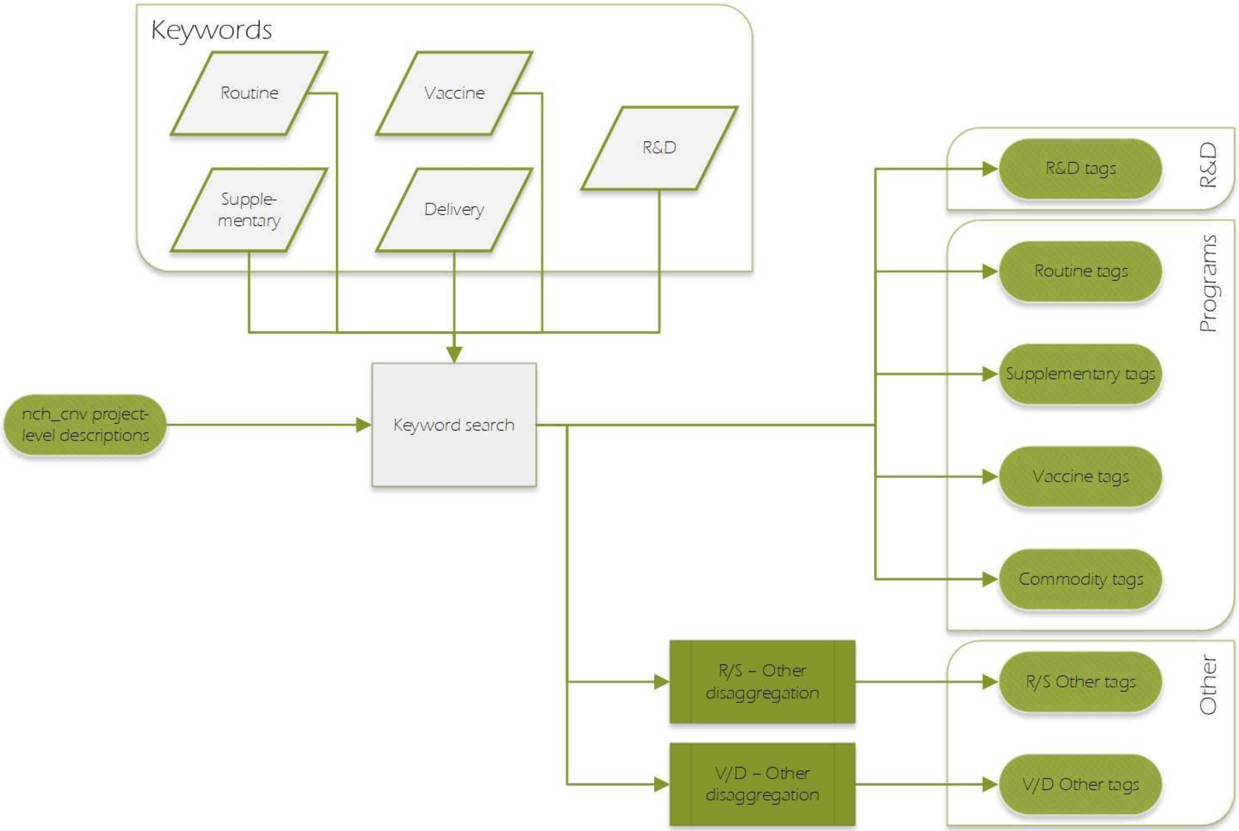

Figure 7.2 Keyword search workflow

Keywords

The following is the list of keywords (or, more accurately, search strings) for each ImmFin program area. The keyword search is currently done in Stata and it is important to note that Stata assumes a wildcard whenever a string ends in a non-space character. For example, if searching for the string " VACCINE ", the program will find " VACCINE " but not " VACCINES ", " VACCINATION ", etc. Conversely, if there is no space at the beginning or end of a search string then Stata assumes a wildcard. For example, " VACCIN" will find " VACCINE ", " VACCINES ", " VACCINATION ", " VACCINATIONS ", etc.

The immunisation financing keywords used are:

ENGLISH
global nch_cnv_rout ABCDEFGHI ///
" IMMUNISATION PROGRAM" " IMMUNISATION PROGRAM" " OUTREACH " ///
" SUSTAINABLE IMMUNIZ" " SUSTAINABLE IMMUNIS" " INTEGRAT" ///
" SUPPORT " " DISTRIBUT" " ESSENTIAL " " PROGRAMMATIC " " ROUTINE " " EPI "
///
" EXPANDED PROGRAMME ON IMMUNI" " EXPANDED PROGRAM ON IMMUNI" ///
" EXPANDED PROGRAMME FOR IMMUNI" " EXPANDED PROGRAM FOR IMMUNI"
///
" IMUNNISATION SERVICE" " IMMUNISATION SERVICE" ///
" IMMUNISATION COVER" " IMMUNISATION COVER" ///
" PERIODIC INTENSIFICATION OF ROUTINE IMMUNI" " PIRI " " P I R I " ///
" VACCINATION PROGRAM" " ELIMINATION PROGRAM"

global nch_cnv_supp ABCDEFGHI ///
" EBOLA " " ZIKA " " AVIAN " " CORONAVIRUS " " SARS " " MERS " ///
" SUPPLEMENTAL " " SUPPLEMENTARY " " CAMPA" " IMMUNISATION PLUS " ///
" IMMUNISATION PLUS " " NIDS " " NID " " IMMUNISATION DAY" ///
" IMMUNISATION DAY" " SIA " " S I A " " POLIO PLUS " " RPM PLUS " " REFUGEE"

global nch_cnv_comm ABCDEFGHI ///
" POLIO " " IPV " " OPV " " DIPTHERIA " " TETANUS " " PERTUSSIS " " DTP " " DTAP "
///
" DPT " " HIB " " ROTAVIRUS " " MEASLES " " HEPB " " HEP B" " RUBELLA " ///
" MENINGITIS " " PENTA" " PNEUMOCOCC" " HAEMOPHILUS INFLU" " TETRA" ///
" POLIOVIRUS " " PCV " " YELLOW FEVER " " CHOLERA " " HIV VACCIN" ///
" MALARIA VACCIN" " DENGUE " " EBOLA VACCIN" " SMALLPOX VACCIN" " BCG "
///
" INFLUENZA " " HPV " " HUMAN PAPILLOMA VIRUS " " JEV " ///
" JAPANESE ENCEPHALITIS " " HEPATITIS B " " MMR " " INJECTION SUPP" "
INJECTION SAFETY DEVICE"

global nch_cnv_deli ABCDEFGHI ///
" PROCUREMENT " " OUTREACH " " TA " " TECHNICAL ASSISTANCE " ///
" EQUIPMENT " " EQPMT " " EQMT " " REFRIGERATOR" " REFRIDGERATOR" "
FRIDGE" ///

" INCREASE AVAILABILITY " " INCREASE THE AVAILABILITY " " INJECTION SAFETY " ///
" COLD CHAIN " " TRAIN" " HSS " " DEMAND " " ADVOCACY " " HARD TO REACH " ///
" HARD-TO-REACH " " MOBILISE " " MOBILIZE " " OPERATIONAL " " CATCH UP " ///
" CATCH-UP " " CATCHUP " " SURVEILLANCE " " SCALE UP " " SCALE-UP " ///
" SCALING UP " " HEALTH INFORMATION " " INFORMATION SYSTEM" ///
" DISTRIBUTION " " STRENGTHEN" " INTEGRATED " " ACCESS " " CAPACIT" ///
" SUPPLY " " ACCELERATE" " CONSTRUCTION " " MEDICAL SUPPL" ///
" SECRETARIAT COST" " CLINIC" /// " DELIVERY "///
" DELIVER" /// copies from swap_hss_level below:
" SWAP" " SECTOR WIDE APPROACH" " DATA SYSTEM" " HEALTH SYSTEM" ///
" SKILLED WORKER" " HEALTH WORKER" " SKILLED STAFF " " HEALTH PROFESSIONAL" ///
" FACILITIES " " POLICY DEVELOPMENT" " INSTITUTIONAL STRENGTHENING " ///
" HSPSP " " M&E " " M & E " " MONITORING " " GOVERNANCE " " HUMAN RESOURCE" ///
" HUMAN CAPITAL " " REALLOCATE RESOURCES " " STRATEGIES AND PROGRAM" ///
" MEDICAL WORKER" " HEALTH CARE PERSONNEL " " OPERATIONAL RESEARCH " ///
" SUPPORTIVE ENVIRONMENT " " WORKFORCE " " INFRASTRUCTUR" ///
" MEDICAL EDUCATION " " HEALTH EDUCATION " " CONTINUING EDUCATION " ///
" HEALTH MANAGEMENT" " HEALTH POLICY " " MANAGEMENT AND COORDINATION " ///
" ADMINISTRATIVE MANAGEMENT " " MANAGEMENT AND ADMINISTRATION " ///
" DSS " " DISTRIBUTION SYSTEMS " " BUILDINGS " " HEALTH FACILIT" ///
"NURSE" "DOCTOR" "PHYSICIAN" "MEDICAL LABORATORY SCIENTIST" "HEALTH LABOR" ///
"LABOR MARKET" "PERSONNEL" "MEDICAL PRACTIONER" "TASK SHIFTING"

global nch_cnv_rd ABCDEFGHI ///
" RESEARCH" /// " DEVELOPMENT " " DEVELOP" ///
" R&D " " TRIAL " " TEST NEW VACCIN" ///
" TEST VACCIN" /// " CLINICAL STUD"
" CLINICAL" " VACCINE CANDIDATE" " MODEL " " AWARD" ///

SPANISH

global nch_cnv_rout_s ABCDEFGHI ///
" PROGRAMA DE INMUNIZACIONES " " ALCANCE " " INMUNIZACION SOSTENIBLE " ///
" INTEGRADO " " APOYO " " DISTRIBU" " ESENCIAL " " PROGRAMATIC" " RUTINA " "PAI " " PROGRAMA AMPLIADO DE INMUNI" ///
" SERVICIO DE IMMUNI" "PROGRAMA DE VACUNA" ///
" INTENSIFICACION PERIODICA DE LA INMUNIZACION SISTEMATICA " " PIRI " "P I R I"
global nch_cnv_supp_s ABCDEFGHI ///

" REFUERZO " ///
" CAMPANA " " INMUNIZACION PLUS " " INMUNIZACION PLUS " " DNI " " DNI "
" DIAS DE INMUNIZACION " " ASI " " A S I " " REFUGIAD" " AVIAR "
global nch_cnv_comm_s ABCDEFGHI ///
" DIFTERIA " " TETANOS " " TOS FERINA" " SARAMPION " " VHB " " RUBEOLA "
" NEUMOCOCO " " HAEMOPHILUS INFLU" " FIEBRE AMARILLA " " COLERA " ///
" VACUNA CONTRA EL VIH " VACUNA CONTRA LA MALARIA " " VACUNA CONTRA EL EBOLA " ///
" VPH " " VIRUS DEL PAPILOMA HUMANO " " ENCEFALITIS JAPONESA " " SPR "
" TRIPLE VIRAL " " VACUNA CONTRA LA VIRUELA "
global nch_cnv_deli_s ABCDEFGHI ///
" DIVULGACION " " ALCANCE " " AT " " ASISTENCIA TECNICA " " EQUIPO " " REFRIGERADOR " ///
" NEVERA" " AUMENTAR DISPONIBILIDAD " " AUMENTAR LA DISPONIBILIDAD " ///
" SEGURIDAD DE LA INYECCION " " CADENA DE FRIO " " FORMACION " " CAPACITACION " ///
" CAMPANA " " HSS " " DEMANDA " " ABOGACIA " " DIFICIL DE ALCANZAR " " MOVILIZAR " ///
" OPERACIONAL " " RECUPERAR " " VIGILANCIA " " AUMENTAR " ///
" SISTEMAS DE INFORMACION " " DISTRIBUCION " " FORTALECER " "REFORZAR" ///
" INTEGRADO" " ACCESO " " CAPACIDAD " " SUMINISTRO " " ACELERAR " " CONSTRUCCION " ///
" SUPLEMENTO MEDICO " "ENTREGA"
global nch_cnv_rd_s ABCDEFGHI ///
" I+D " " I + D " " INVESTIGAC" "DESARROLL" " ENSAYO" " PRUEBA DE VACUNA" " CLINIC" ///
" MODELO "

FRENCH

global nch_cnv_rout_f ABCDEFGHI ///
" CAMPAGNE DE VACCINATION" " CAMPAGNE D IMMUNISATION" " SENSIBILISATION " ///
" IMMUNISATION DURABLE" " VACCINATION DURABLE" " INTEGRE" ///
" SOUTIEN " " APPUI " " DISTRIBUT" " ESSENTIEL " " INDISPENSABLE " " PROGRAMMATIQUE " ///
" ROUTINE " " EPI " ///
" PROGRAMME ELARGI D IMMUNISATION" " PROGRAMME ELARGI DE VACCINATION" ///
" SERVICE D’IMUNNISATION" " SERVICE DE VACCINATION" ///
" COUVERTURE D IMMUNISATION" " COUVERTURE DE VACCINATION" ///
" PROGRAMME DE VACCINATION" " PROGRAMME D IMMUNISATION" " PNI " " P N I " ///
" PROGRAMME NATIONAL DE VACCINATION" " PROGRAMME NATIONAL D IMMUNISATION" ///

" INTENSIFICATION PERIODIQUE DE LA VACCINATION DE ROUTINE" " IPVR " " I P V R " ///

global nch_cnv_supp_f ABCDEFGHI ///
" EBOLA " " ZIKA " " GRIPPE AVIAIRE " " CORONAVIRUS " " SRAS " " SRMO " ///
" SUPPLEMENTAIRE " " CAMPA" " IMMUNISATION PLUS " ///
" IMMUNISATION PLUS " " JNV " " JOURNEE DE VACCINATION" ///
" JOURNEE D IMMUNISATION " " AVS" " A V S " " POLIO PLUS " " RPM PLUS " ///
" REFUGIE"
global nch_cnv_comm_f ABCDEFGHI ///
" POLIO " " POLIOMYELITE " " VPI " " VPO " " DIPTHERIE " " TETANOS " " PERTUSSIS " ///
" DTC " " DTAC " " DCT " " HIB " " ROTAVIRUS " " ROUGEOLE " " HEPB " " HEP B" ///
" RUBEOLLE " " MENINGITE " " PENTA" " PNEUMOCOQUE " " HAEMOPHILUS INFLU" " HIB DTC"
" VIRUS DE LA POLIOMYELITE " " VPC " " FIEVRE JAUNE " " CHOLERA " ///
" VACCIN CONTRE LE VIH" " VACCIN ANTIPALUDIQUE" " DENGUE " " VACCIN EBOLA" ///
" VSV EBOV " " VACCIN ANTIVARIOLIQUE " " BCG " " GRIPPE " " PVH " ///
" PAPILLOMAVIRUS HUMAIN " " VEJ " " ENCEPHALITE JAPONAISE " " HEPATITE B " " ROR "
global nch_cnv_deli_f ABCDEFGHI ///
" APPROVISIONNEMENT " " SENSIBILISATION " " AT " " ASSISTANCE TECHNIQUE " ///
" EQUIPEMENT " " EQPMT " " EQMT " " REFRIGERATEUR" " FRIGO" ///
" AUGMENTER LA DISPONIBILITE " " INJECTION SECURISEE " " LA CHAINE DU FROID " ///
" FORMATION" " SERVICES DE SANTE " " DEMANDE " " PLAIDOYER " ///
" DIFFICILE A ATTEINDRE " " DIFFICILE D ACCES " " MOBILISER " " OPERATIONNELLE " ///
" RATTRAPAGE " " SURVEILLANCE " " ELARGIR " " ELARGI " " INFORMATION SANITAIRE " ///
" SYSTEME D INFORMATION " " DISTRIBUTION " " RENFORCER " " INTEGRE " " ACCES " ///
" CAPACIT" " OFFRE " " MATERIEL " " ACCELERER" " CONSTRUCTION " " MATERIEL MEDICAL" ///
" SECRETARIAT COST" " LIVRAISON " " LIVRER"
global nch_cnv_rd_f ABCDEFGHI ///
" RECHERCHE" " DEVELOPPEMENT" " DEVELOPPER" " R&D " " ESSAI " ///
" TEST DE NOUVEAU VACCIN" " TEST VACCIN" " CLINIQUE" " VACCIN CANDIDAT" " MODELE " ///
" RECOMPENSE "

"Other" disaggregation

Within each of the two immunisation financing program buckets (routine/supplementary and vaccine/delivery), any programs that do not get tagged based on the keyword search are moved

into a respective “other” bucket. This explains why there are no keywords for “other routine/supplementary” or “other vaccine/delivery”.

S4.3 Methods

A description of the methods used for each channel is provided here.

Gavi

Disaggregation of Gavi’s disbursements by program to routine or supplementary/commodity or delivery categories

A key deliverable of the immunisation financing project is to determine funding amounts spent on routine versus supplementary activities, and how much is spent on commodities versus delivery based activities. In addition to other available data sources for both domestic funding and development assistance, we will disaggregate Gavi disbursements into the same categories based on the program types and definitions.

Between 2000 to date, Gavi’s disbursements have been classified into 12 unique high level categories with their corresponding disbursements by year paid. These include; cold chain equipment optimization program (CCEOP), civil society organization (CSO type A and B), cash support, Ebola EPI recovery grant, graduation grant, health systems support(HSS), injection safety support(ISS), immunisation system strengthening(ISS), new vaccine support(NVS), operational support, product switch grant and vaccine introduction grant. These categories encompass different programs labelled as sub-categories for which the disbursements are tagged to. New vaccine support and operational support have further been disaggregated into 19 and 12 sub-categories respectively. In addition, 18 subcategories are listed without a corresponding high level category. We also include disbursements for 21 additional programs listed under investment cases (emergency outbreak support and other strategic investments) bringing the total number of unique programs/sub-categories to 80.

Commodity versus delivery based disbursements

Based on the category, sub-category and program definitions, disbursements for vaccine introduction grants, new vaccine support, investment cases (excluding any operational support listed therein), campaigns, catch-up activities or outbreak responses targeting specific vaccines and will be categorized as commodity based spending. Categories or program types including CCEOP, cash support, operational support, graduation grants, product switch grants, CSO, HSS, ISS, INS, will be categorized as delivery based including investment cases for operational support, WHO debt relief and Vodafone programs. The Ebola EPI recovery grants will be disaggregated as per the specific amounts that were spent on different activities ranging from vaccine campaigns, cold chain optimization and social mobilization. The aggregated amount from these two categories should ideally match total disbursements.

Spending on routine versus supplementary immunisation activities

Additionally, disbursements will be grouped into routine or supplementary activities where routine spending will reflect programs disbursements that support day to day implementation of immunisation activities while the supplementary category will reflect any additional activities driven by particular contextual factors within recipient countries. Examples of such contextual factors include declines in facility based vaccine uptake or delivery necessitating localized or nation-wide campaigns, a strain on the immunisation program as a result of existing health crises such as the Ebola crisis in West Africa, or transition from eligibility for countries meeting the criteria established for graduation. As such, program disbursements tagged to different campaigns, graduation grants, Ebola EPI recovery grants and vaccine pilot implementation will be classified as supplementary. All other programs types i.e. CCEOP, operational support, product switch grants, CSO, HSS, ISS, INS, other investment cases (excluding vaccine pilots) will be categorized as routine. As for the disaggregation above, the aggregate amount from these two main components will match total disbursements.

High level category	Sub-category	Vaccine/Delivery	Routine/Supplementary
**CCEOP**	CCEOP	Delivery	Routine
**CSO**	CSO A, CSO B	Delivery	Routine
**Cash support**	HPV Demo - cash support	Delivery	Routine
**Ebola EPI recovery plan**	Ebola EPI recovery plan	*Reference: Ebola Mitigation plan 2015*	Supplementary
**Graduation grant**	Graduation grant	Delivery	Supplementary
**HSS**	HSS	Delivery	Routine
**INS**	INS	Delivery	Routine
**Product switch grant**	Product switch grant	Delivery	Routine
**Vaccine introduction grant**	Vaccine introduction grant	Vaccine	Routine
**NVS**	HPV<br>HPV Demo<br>HepB mono<br>Hib mono<br>IPV<br>JEV<br>Measles<br>Measles SIA<br>Measles-Rubella<br>Meningitis A<br>Meningitis A – campaign<br>Meningitis A - mini catch-up campaign<br>Penta<br>Pneumo<br>Rotavirus<br>Tetra DTP-HepB<br>Tetra DTP-Hib<br>Yellow Fever<br>Yellow Fever - campaign	Vaccine	Campaigns<br>= Supplementary<br><br>All other programs<br>= Routine

**Operational support**	HPV MAC - Op costs<br>JEV - Operational costs<br>MR - Operational costs<br>MR-Catch-up campaign op.costs<br>MR-Follow-up campaign op.costs<br>Measles SIA - Operational costs<br>Measles-Follow-up campaign op.costs<br>Meningitis A - mini catch-up op.costs<br>Meningitis A - operational costs<br>Operational costs<br>TCV - Outbreak op. costs<br>YF - Operational costs	Delivery	Campaign related operational costs = Supplementary<br><br>All other programs<br>= Routine
**No program type**	Additional Intro Support<br>HPV MAC<br>HPV MAC - Op costs<br>IPV Catch-up RI<br>Injection Safety Devices<br>JEV-Routine<br>MR 1st and 2nd dose<br>MR 1st dose<br>MR 2nd dose<br>MR-Catch-up campaign<br>MR-Follow-up campaign<br>Measles 1st and 2nd dose<br>Measles-Catch-up campaign<br>Measles-Follow-up campaign<br>Penta – campaign<br>Pneumo – campaign<br>TCV Outbreak<br>Td - campaign	HPV MAC – Op costs<br>Injection Safety Devices<br>= Delivery<br><br>All other programs<br>=Vaccine	Campaign related programs<br>= Supplementary<br><br>All other programs<br>= Routine

High level category	Program	Vaccine/Delivery	Routine/Supplementary
**Investment case**	MNT<br>MNT - operational costs<br>Measles<br>Measles - operational costs<br>Measles-Rubella<br>Measles-Rubella - operational costs<br>Meningitis<br>Meningitis - operational costs<br>Merck - Adv purchase comm – Ebola<br>Mongolia – Vodafone<br>Polio<br>Polio - operational costs<br>UNICEF – CCEOP<br>UNICEF - vaccine stockpile<br>Vaccine Independence Initiative<br>WHO - Debt Relief Study<br>WHO - Vaccine pilot implementation<br>WHO - operational costs<br>WHO - vaccine stockpile<br>Yellow Fever<br>Yellow Fever - operational costs	Operational costs,<br>Mongolia Vodafone,<br>UNICEF CCEOP,<br>WHO Debt relief study,<br>WHO - Vaccine pilot implementation,<br>WHO - vaccine stockpile,<br>Merck - Adv purchase comm – Ebola<br>= Delivery<br><br>All other programs<br>= Vaccine	Merck - Adv purchase comm – Ebola,<br>WHO - Vaccine pilot implementation<br>= Supplementary<br><br>All other programs<br>= Routine
**Other**	TCA	Delivery	Routine & Supplementary (50%)

Disaggregation of ebola EPI recovery grants by country in 2015	
**Country**	**Commodity/Delivery**
**Liberia ($2.9m)**	
**MCV campaigns**	Commodity

**Polio and measles SIA**	Commodity
**Guinea ($6.05m)**	
**Measles ORI**	Commodity
**Penta ORI**	Commodity
**Social mob/communication ($334,518)**	Delivery
**Cold chain & other operational costs ($1.9m)**	Delivery
**Sierra Leone ($4.3m)**	
**Polio SIA**	Commodity
**Polio and measles immunisation**	Commodity

Table 5. Gavi funding disaggregation

PAHO

No project-level data exists; data was not included.

World Health Organization

No project-level data exists; Instead WHO project fractions are created using WHO projects in the CRS and applied to the WHO envelope from the FGH 2019 database. For 2008 to 2011, WHO immunisation disbursement is 0. To correct for the missing data in these years, total immunisation spending was linearly interpolated for these years.

African Development Bank

There were no relevant immunisation disbursements in the African development bank envelope in the FGH 2019 data and so African development bank was not included in the analyses.

Asian Development Bank

There are very few immunisation disbursements in the project level dataset and so program areas are manually assigned at the project level.

Years	Project ID	Project Name	Project Description	Immunisation assignment
2016-2019	3257	Supporting National Urban Health Mission	ADB is reinforcing the efforts of Government of India to improve the health of people who live in cities, especially the poor. The project is strengthening broadly-defined urban health systems across cities and towns to deliver quality essential health services for all, with a particular focus on the poor and vulnerable. The project is improving the networks of primary health facilities in urban areas and introducing a quality assurance mechanism for them. It is also helping to improve planning, management, and innovation, in order to bring best practices to city clinics and health centers. The financing for the project is being disbursed based on the achievement of results, including more births in health facilities and higher childhood immunisation rates	Delivery, Routine
2018	3736	System strengthening for effect coverage of new vaccines, Pacific	This proposed Supporting Effective Coverage of Health Technology in the Pacific (the project) will form part of the regional response to reduce the number of cervical cancer cases and other infectious diseases in children and women. The project proposes to use the introduction of new vaccines and early detection through communicable disease platforms to drive necessary improvements in the public health system in selected Pacific DMCs in line with the Asia Pacific Strategy for Emerging Diseases and Public Health Emergency	Delivery, Routine

Table 6.

InterAmerican Development Bank

There are very few immunisation disbursements in the project level dataset and so program areas are manually assigned at the project level.

World Bank

There are very few immunisation disbursements in the project level dataset and so program areas are manually assigned at the project level.

**Channel**	**Years**	**Project ID**	**Project Name**	**Purpose(s)**	**Immunisation assignment**
World Bank	2000-2005	P067330	Immunisation Strengthening Project	Rural services and infrastructure, Participation and civic engagement, Child health, Urban services and housing for the poor	Routine, Vaccine/Delivery Other
World Bank	2017	P132308	National Immunisation Support Project	Other communicable diseases, Child health, Health system performance	Other

Table 7.

Creditor Reporting System

The CRS channel uses a modified version of the standard DAH pipeline. See FGH 2019 Report Annex[1]. Modifications include the updated keyword search and Global Polio Eradication Initiative (GPEI) project reassignment where GPEI projects were assigned completely to supplementary spending. Additionally, negative disbursements were altered so that all negative immunisation disbursements were added to other positive immunisation disbursements.

Non-Governmental Organization

The NGOs channel uses a modified version of the standard DAH pipeline. See FGH 2019 Report Annex[1]. Modifications included using the updated keyword search and application of those keyword search results in immunisation component assignment.

Bill and Melinda Gates Foundation

The Gates Foundation channel uses a modified version of the standard DAH pipeline. See FGH 2019 Report Annex[1]. Modifications include the updated keyword search and Global Polio

Eradication Initiative (GPEI) project reassignment where GPEI projects were assigned completely to supplementary spending.

US Foundations

The US Foundations channel uses a modified version of the standard DAH pipeline. See FGH 2019 Report Annex[1]. Modifications included using the updated keyword search and application of those keyword search results in immunisation component assignment.

United Nations Children's Fund

An outline of the methods used for UNICEF follows.

Generating IATI data

We use the IATI query tool (http://datastore.iatistandard.org/query/) with the following filters to build the UNICEF dataset used for project descriptions:

- Reporting organization: United Nations Children's Fund (UNICEF): XM-DAC-41122
- Sector codes: 12110, 12181, 12182, 12191, 12220, 12230, 12250, 12261, 12262, 12263, 12281, 12310, 12350, 12382

Note that a search for "UNICEF" for the reporting organization also shows "Unicef Belgium National Committee : BE-BCE_KBO_0407562029" and "UNICEF Netherlands National Committee : NL-KVK-27102631"; neither of these are used here.

Cleaning data

The IATI dataset has many variables, most of which are not useful for our purposes. The variables kept are:

- transactiondate
- transactionvalue – the disbursement amount in USD
- title – descriptive project title
- description – project description
- transaction_ref – type of transaction: commitment, disbursement, expense, or incoming funds
- reportingorg – the channel, ie UNICEF
- recipientcountry
- iatiidentifier – project ID
- participatingorgFunding
- participatingorgExtending
- participatingorgImplementing

Running keyword search

The keyword search is then run on the dataset across the title and description variables. This outputs keyword counts and fractions for all DAH program areas; we drop everything except the relevant ones (_supp, _rout, _deli, _comm, and _rd).

Determining relevant transaction type

There are four unique transaction_ref (transaction types) for the UNICEF data: commitment, disbursement, expense, and incoming funds. An exploration of the data along with the UNICEF DAH dataset found that the "expense" transaction type tracks somewhat consistently with DAH (~50-75%). It is unclear whether "expense" is the same as the IATI-defined "Expenditure", but we assume it is. We therefore keep only transactions of type "expense" and base the fractions on that subset.

Account for double counting other channels

Some of the transactions in the IATI data are not actually from the UNICEF channel but are instead from other channels that we track separately in the standard DAH pipeline. To prevent double counting of these transactions, we drop them if:

- participatingorgImplementing is "CIVIL SOCIETY ORGANIZATION: National NGO", "CIVIL SOCIETY ORGANIZATION: International NGO", or "CIVIL SOCIETY ORGANIZATION: Academic Institution"
- participatingorgFunding contains the strings "African Development Bank", "Gates Foundation", "BMGF", "GAVI", "Pan American Health Org", "PAHO", "Rotary International", "The Global Fund to Fight AIDS", "UNAIDS", "UNFPA", "UNITAID", "UNOCHA", "WHO", or "World Bank"

Calculate fractions

Each transaction is disaggregated into program areas by multiplying the total transaction by the fractions calculated from the keyword search (routine, supplementary, other routine/supplementary, vaccine, delivery, other vaccine/delivery, and R&D).

The generated expenses are then aggregated by year and then annual fractions calculated by dividing the program area expenses by the total expenses for the year.

The generated yearly fractions are then scaled to be per immunisation fraction. This is done so that the immunisation fractions can be used directly with immunisation envelope rather than total DAH.

Expand to all years of interest

The IATI data only includes UNICEF data for 2012+. For previous years, we assume that each program area fraction (routine, supplementary, other routine/supplementary, vaccine, delivery, other vaccine/delivery, and R&D) is an average of the annual fractions for 2012+.

Estimating UNICEF Immunisation DAH

With the program area fractions (per year) calculated, we then load the DAH dataset, subset to the UNICEF channel and years of interest, drop observations with that are flagged for double counting and merge on the fractions dataset (by year). Finally, we obtain the estimated disbursements by multiplying the immunisation envelope values by the fractions.

Section 5. Estimating prepaid private spending on immunisation

We leveraged the relation between the reported values of prepaid private spending on immunisation from the National Health Accounts to the proportion of prepaid private spending in the entire health sector to generate estimates of prepaid private spending on immunisation. The steps below detail how the estimates were calculated.

1. Calculate A = PPP/(non-PPP) for health sector by country year
2. Calculate B = PPP/(non-PPP) for immunisation by country year (using NHA data)
3. Calculate rho = median(B/A) (across country/years)
4. Calculate Imm. PPP = rho * PPP/(non-PPP) for health sector * non-PPP for immunisation

Section 6. Aggregating total spending on immunisation

We aggregated total spending on immunisation estimates using the estimates of all financing sources (government spending, out-of-pocket spending, development assistance for health, and prepaid private spending). All estimates for individual financing sources, excluding development assistance for health, were produced with 1000 draws or estimates. To ensure valid statistical error, the estimates were aggregated at the draw level. We averaged the estimates by draw to produce estimates for total spending on immunisation. This same method was used to aggregate the immunisation components and to produce estimates by country year.

Section 7. Currency conversion

All immunisation expenditure estimates were made and reported in 2019 United States dollars (USD). Data sources reported expenditure in either nominal local currency units (LCUs) or nominal USD. To convert nominal LCUs to USD, we applied deflators to nominal LCUs to inflate to 2019 LCUs. We then applied exchange rates to produce 2019 USD. When LCUs were not reported, we extracted reported expenditure in nominal USD, applied corresponding nominal exchange rates to produce nominal LCUs, inflated nominal LCUs to 2019 LCUs with deflators, and finally exchanged 2019 LCUs to 2019 USD. All deflators and exchange rates were extracted from the World Bank, International Monetary Fund, Penn World Tables, the United Nations National Accounts and the World Health Organisation, and were imputed to provide a complete series for each of the variables between 1950 and 2019. We then used several models including ordinary least-squares regression and mixed effects models, to complete each source series. More information about the approach to converters and deflators may be found in GBD Health Financing Collaborator Network (2020)[2].

Section 8. Additional results

Figure showing annualized rate of change of number of Penta doses, number of MCV1 doses, LRTI incidence in children under 5, Diarrhoeal disease incidence in children under 5, and annualized rate of change in total immunisation spending per surviving infant between 2010 and 2017. Surviving infant population is calculated using live births and infant mortality rate from the Global Burden of Disease 2019 study data. Disease incidence are from the Global Burden of Disease 2017 study data.

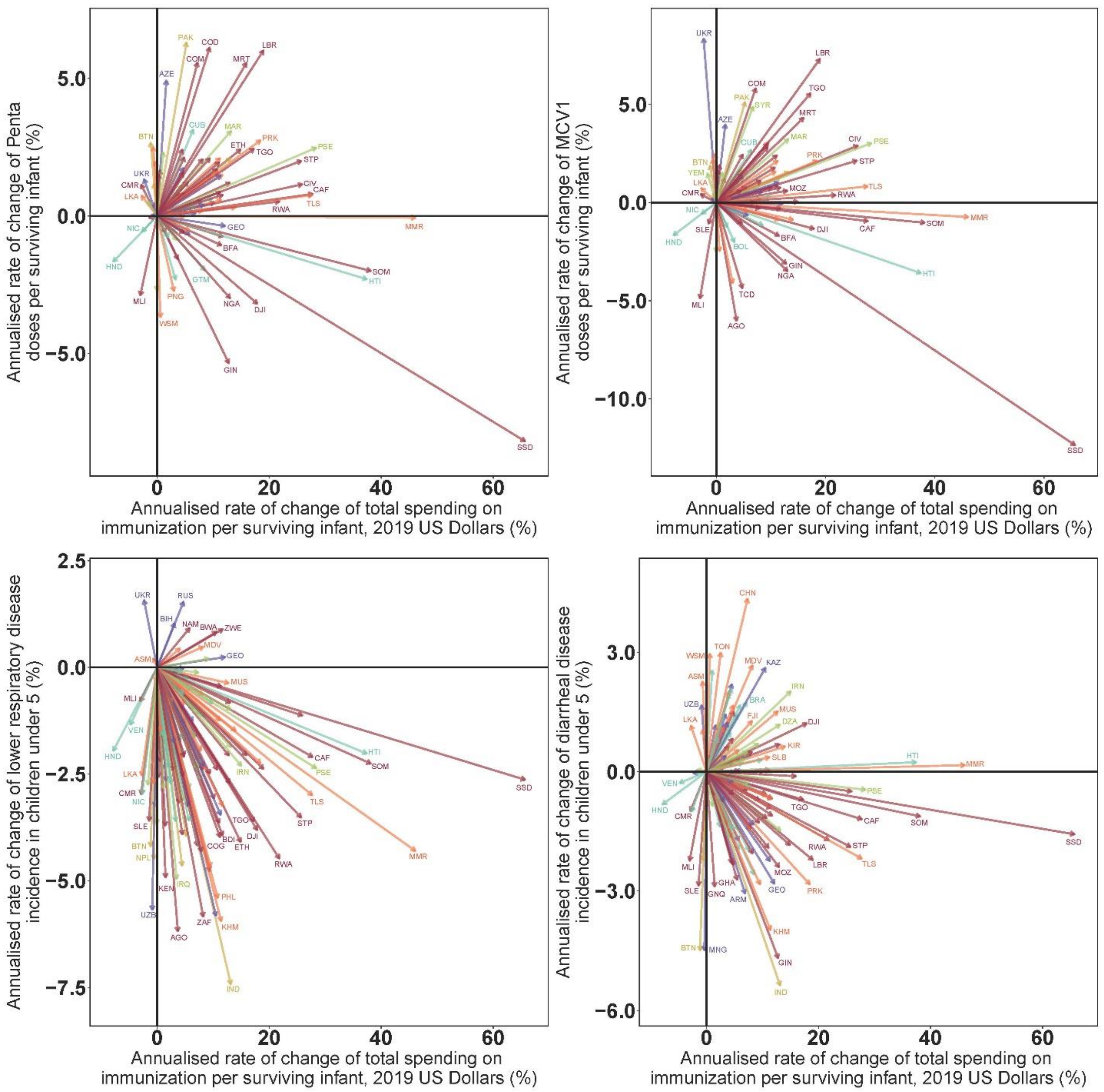

Figure 8.

Figures showing estimates of spending per surviving infant in 135 countries by funding source and immunisation activity, component, or total for 2017. Countries outside of the scope or with values of zero are marked in grey. Colors represent quintiles. Total out-of-pocket spending on immunisations is equivalent to out-of-pocket spending on routine immunization.

Total spending on immunisations per surviving infant, 2017

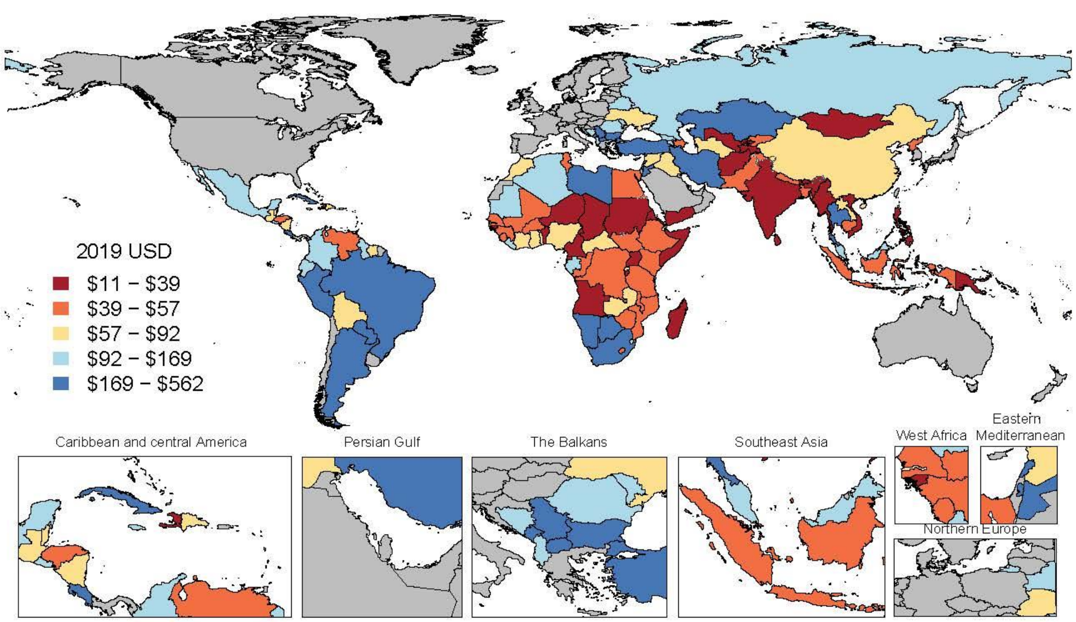

Government spending on immunisations per surviving infant, 2017

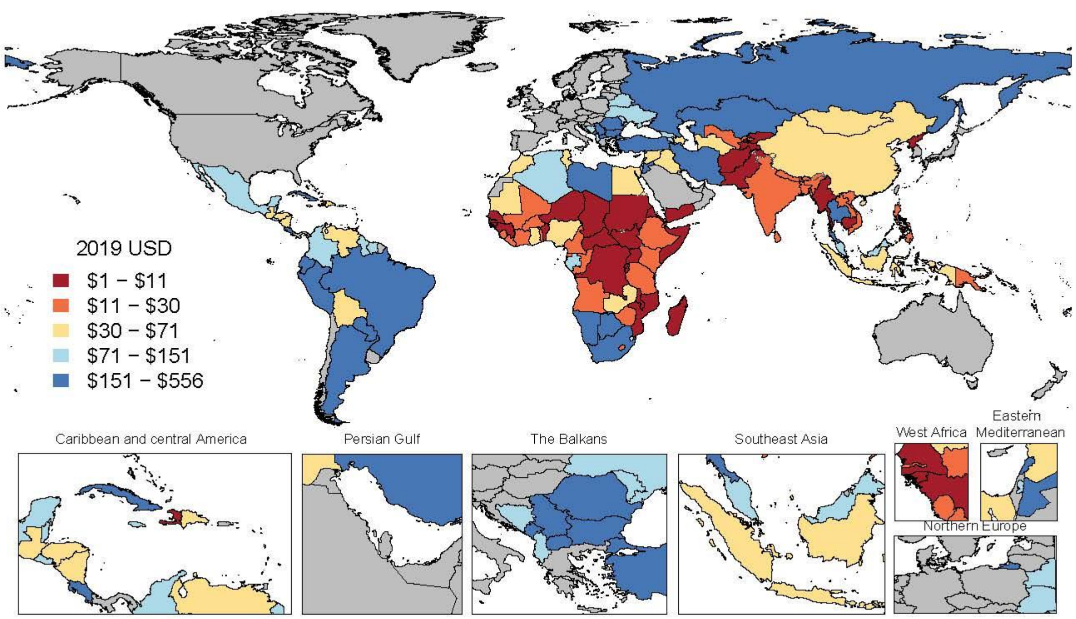

Development assistance on immunisations per surviving infant, 2017

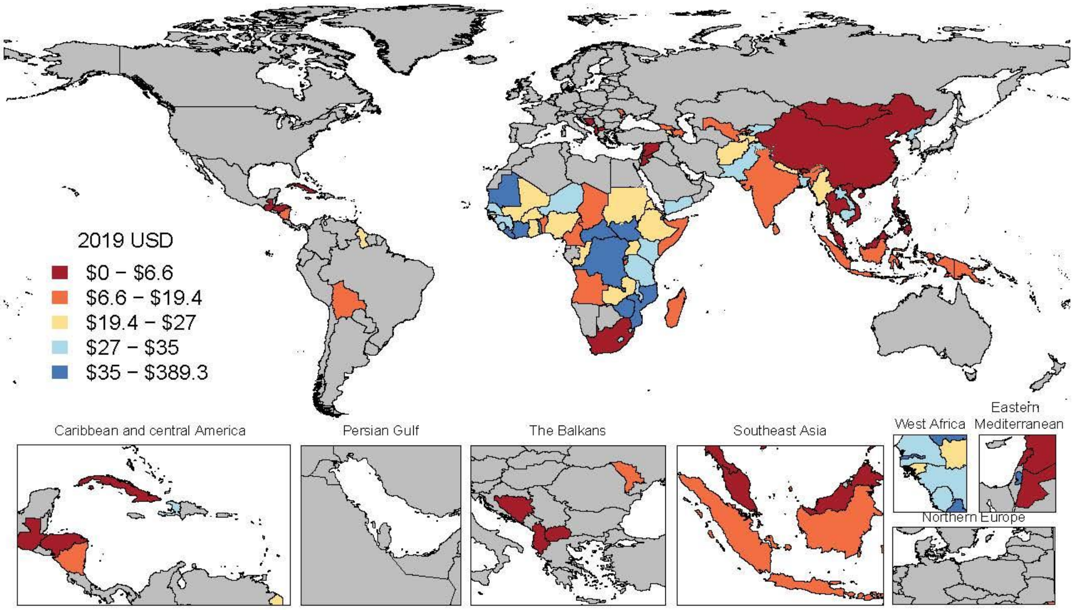

Out-of-pocket spending on immunisations per surviving infant, 2017

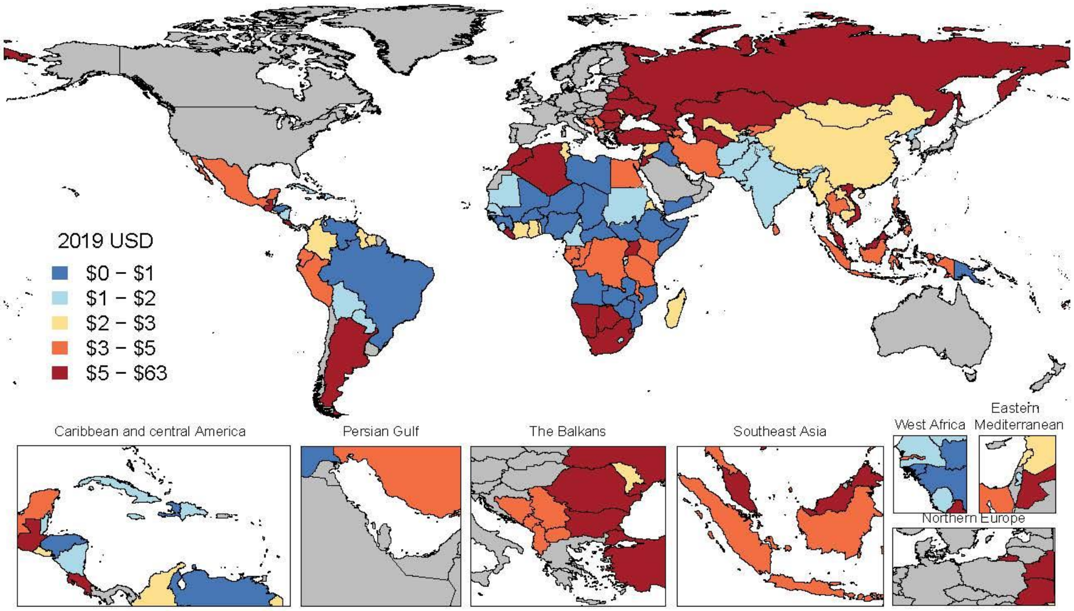

Prepaid private spending on immunisations per surviving infant, 2017

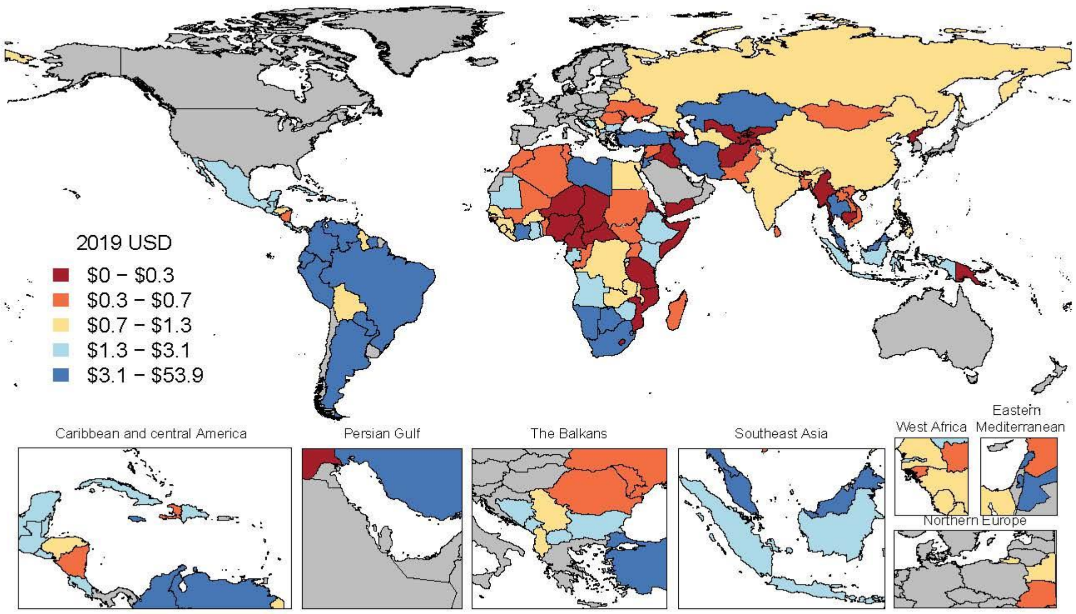

Government spending on routine immunization per surviving infant, 2017

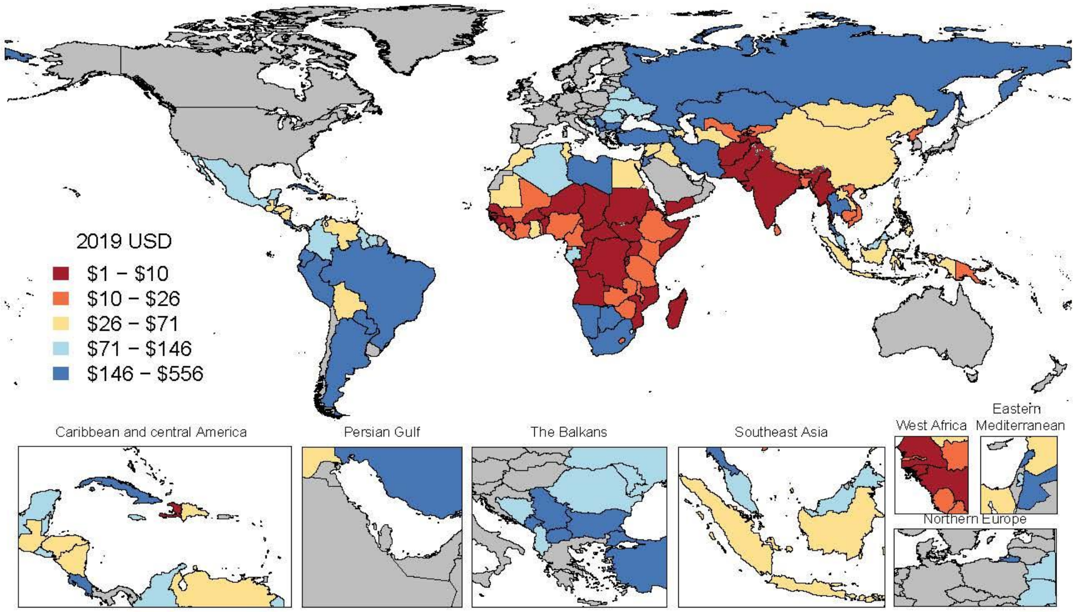

Government spending on supplementary immunization per surviving infant, 2017

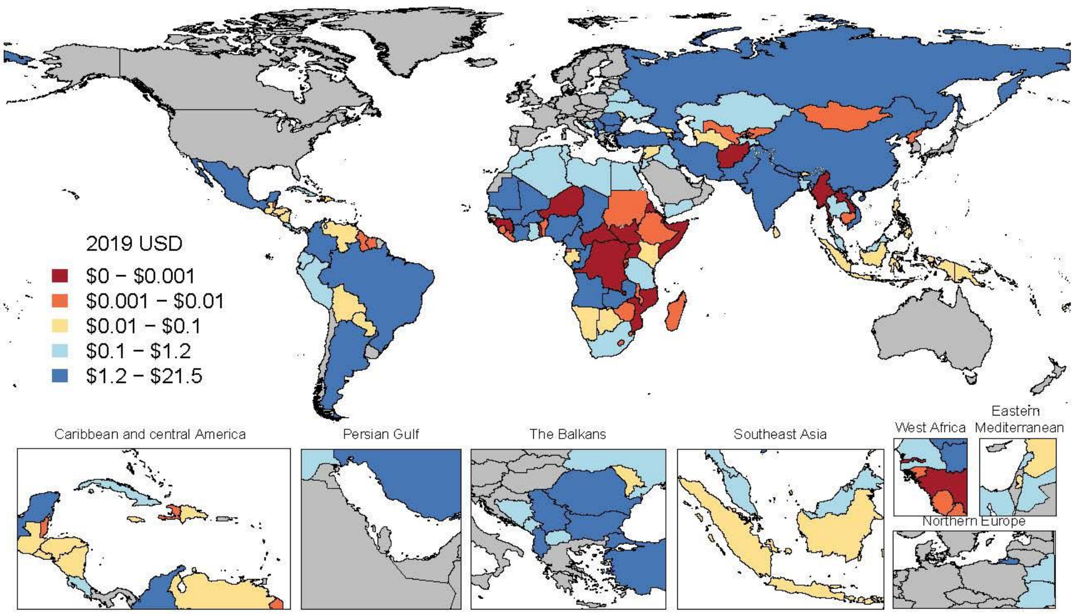

Government spending on delivery per surviving infant, 2017

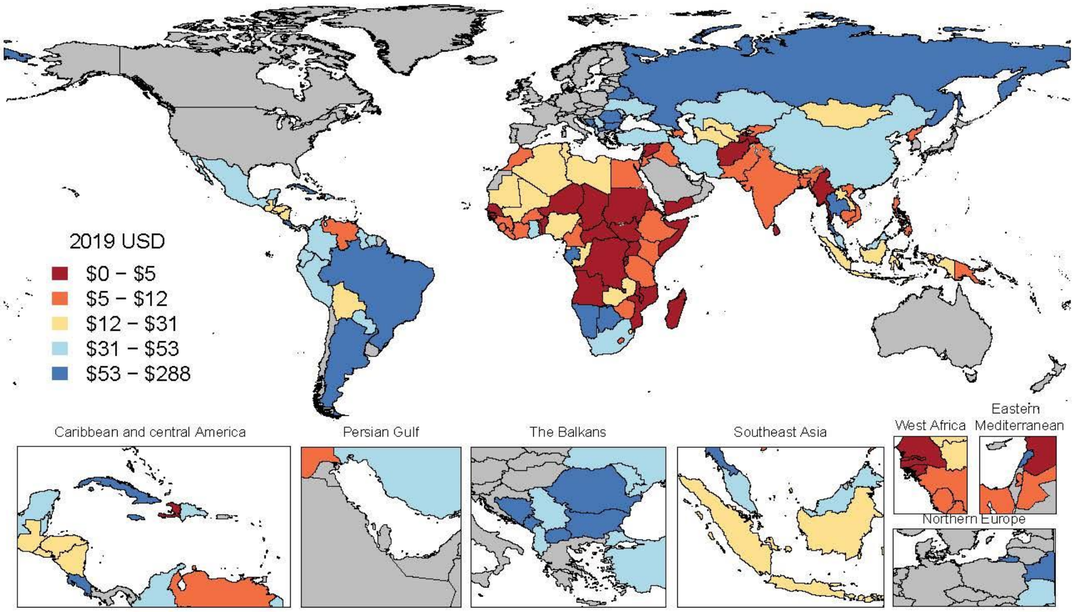

Government spending on vaccines per surviving infant, 2017

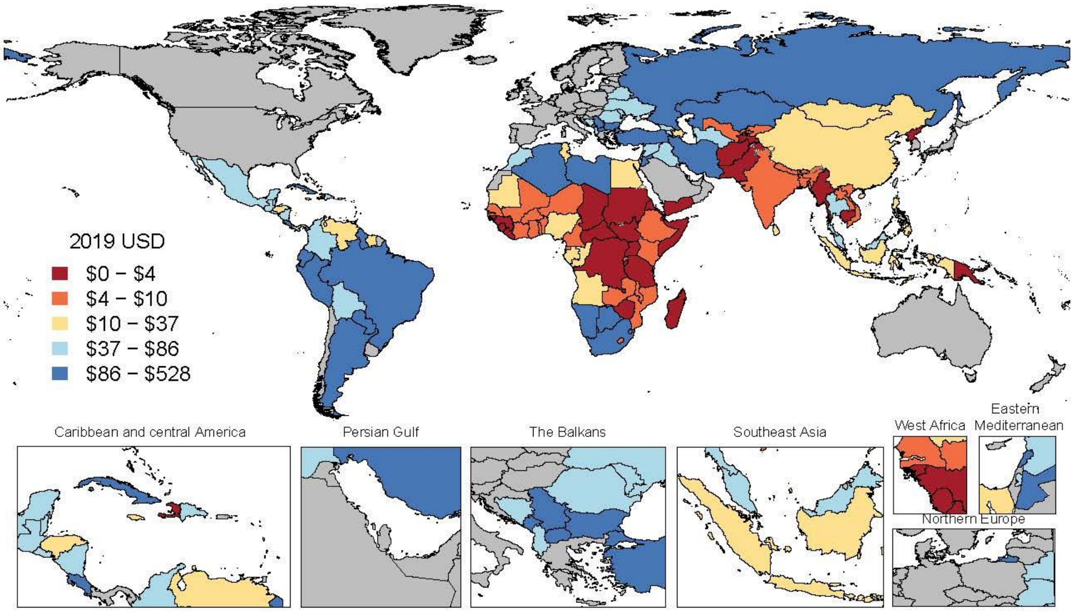

Development assistance for routine immunization per surviving infant, 2017

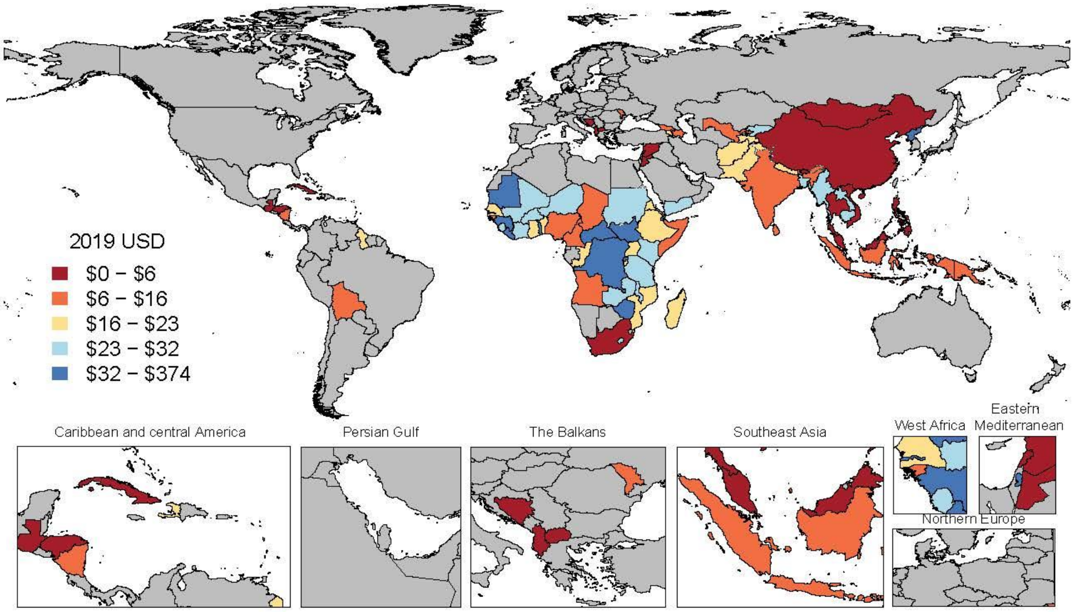

Development assistance for supplementary immunization per surviving infant, 2017

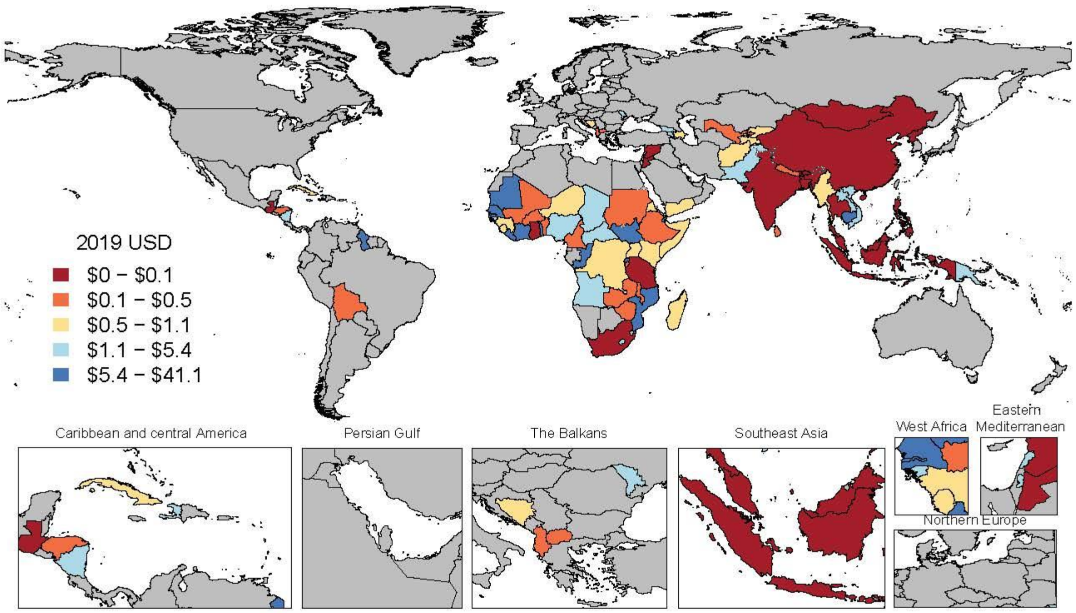

Development assistance for delivery per surviving infant, 2017

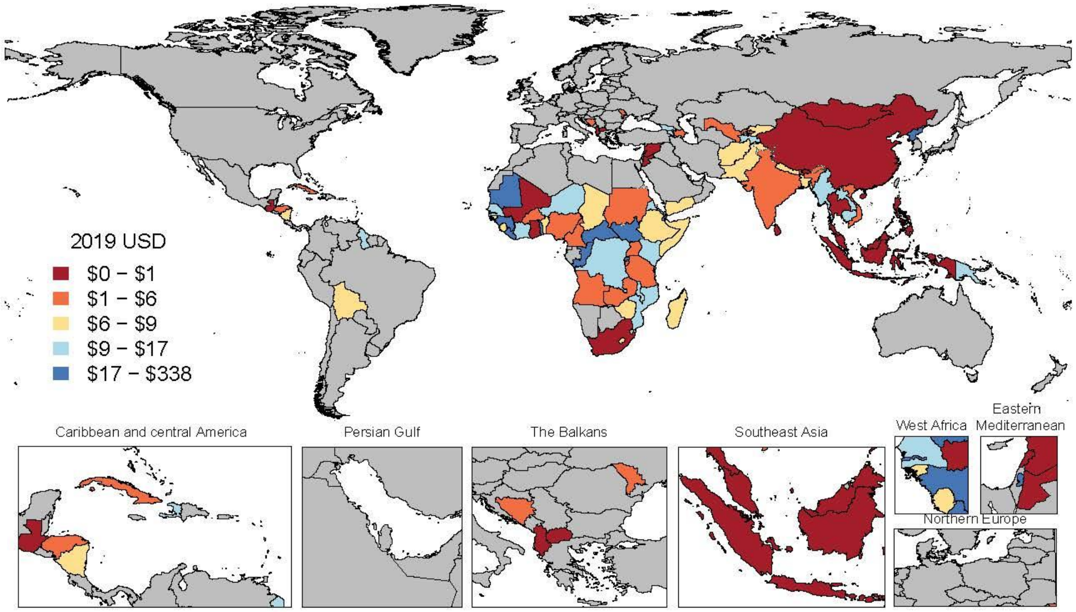

Development assistance for vaccines per surviving infant, 2017

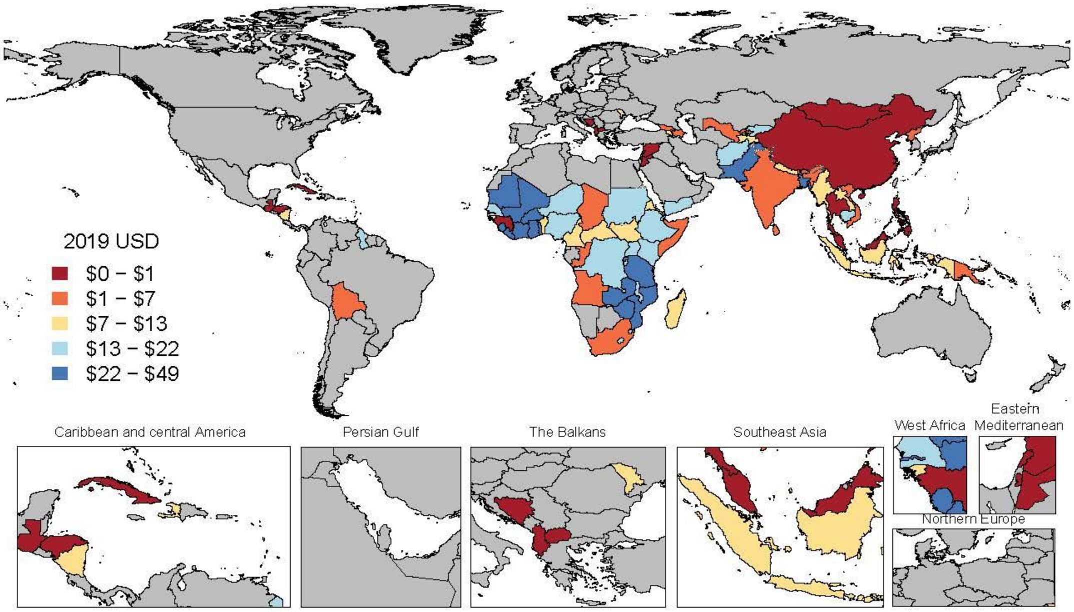

Out-of-pocket spending on delivery per surviving infant, 2017

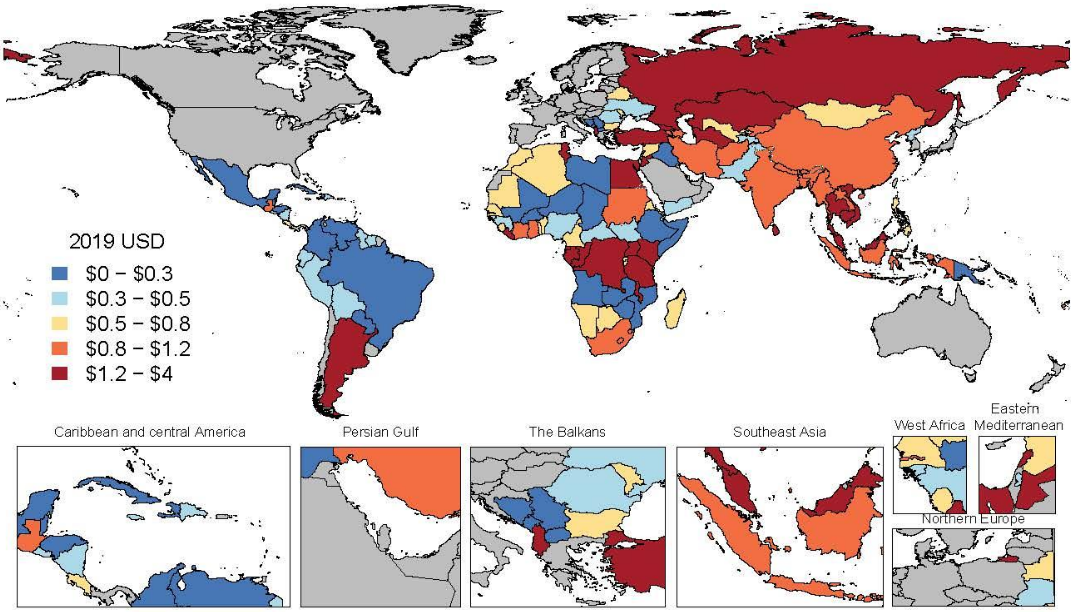

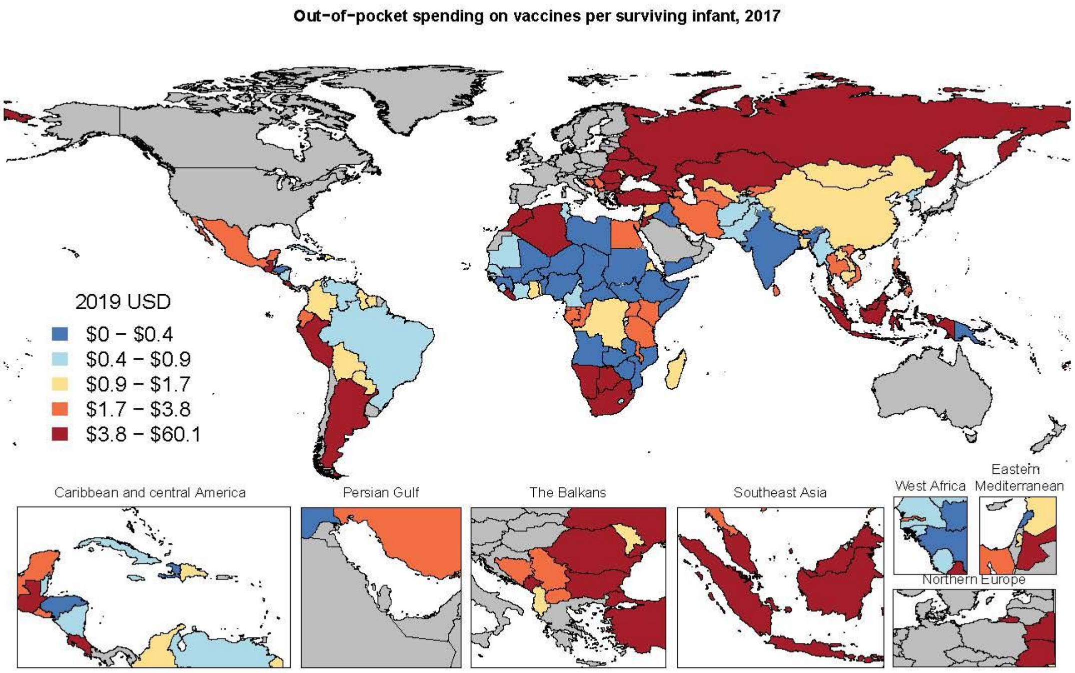

Figure 9. Maps of spending or funding by financing source and immunisation activities or components, 2017

Country	Gavi country	Income classification	GBD super region
Afghanistan	Yes	Low-income	North Africa and Middle East
Albania	No	Upper middle-income	Central Europe, Eastern Europe, and Central Asia
Algeria	No	Upper middle-income	North Africa and Middle East
American Samoa	No	Upper middle-income	Southeast Asia, East Asia, and Oceania
Angola	Yes	Lower middle-income	Sub-Saharan Africa
Argentina	No	Upper middle-income	Latin America and Caribbean
Armenia	Yes	Upper middle-income	Central Europe, Eastern Europe, and Central Asia
Azerbaijan	Yes	Upper middle-income	Central Europe, Eastern Europe, and Central Asia
Bangladesh	Yes	Lower middle-income	South Asia
Belarus	No	Upper middle-income	Central Europe, Eastern Europe, and Central Asia
Belize	No	Upper middle-income	Latin America and Caribbean
Benin	Yes	Low-income	Sub-Saharan Africa
Bhutan	Yes	Lower middle-income	South Asia
Bolivia	Yes	Lower middle-income	Latin America and Caribbean
Bosnia and Herzegovina	No	Upper middle-income	Central Europe, Eastern Europe, and Central Asia
Botswana	No	Upper middle-income	Sub-Saharan Africa
Brazil	No	Upper middle-income	Latin America and Caribbean
Bulgaria	No	Upper middle-income	Central Europe, Eastern Europe, and Central Asia
Burkina Faso	Yes	Low-income	Sub-Saharan Africa
Burundi	Yes	Low-income	Sub-Saharan Africa
Cape Verde	No	Lower middle-income	Sub-Saharan Africa
Cambodia	Yes	Lower middle-income	Southeast Asia, East Asia, and Oceania
Cameroon	Yes	Lower middle-income	Sub-Saharan Africa
Central African Republic	Yes	Low-income	Sub-Saharan Africa
Chad	Yes	Low-income	Sub-Saharan Africa
China	No	Upper middle-income	Southeast Asia, East Asia, and Oceania
Colombia	No	Upper middle-income	Latin America and Caribbean
Comoros	Yes	Lower middle-income	Sub-Saharan Africa
Congo (Brazzaville)	Yes	Lower middle-income	Sub-Saharan Africa
Costa Rica	No	Upper middle-income	Latin America and Caribbean
Cuba	Yes	Upper middle-income	Latin America and Caribbean
CÃ´te d'Ivoire	Yes	Lower middle-income	Sub-Saharan Africa
North Korea	Yes	Low-income	Southeast Asia, East Asia, and Oceania
DR Congo	Yes	Low-income	Sub-Saharan Africa
Djibouti	Yes	Lower middle-income	Sub-Saharan Africa
Dominica	No	Upper middle-income	Latin America and Caribbean
Dominican Republic	No	Upper middle-income	Latin America and Caribbean
Ecuador	No	Upper middle-income	Latin America and Caribbean
Egypt	No	Lower middle-income	North Africa and Middle East

El Salvador	No	Lower middle-income	Latin America and Caribbean
Equatorial Guinea	No	Upper middle-income	Sub-Saharan Africa
Eritrea	Yes	Low-income	Sub-Saharan Africa
eSwatini	No	Lower middle-income	Sub-Saharan Africa
Ethiopia	Yes	Low-income	Sub-Saharan Africa
Fiji	No	Upper middle-income	Southeast Asia, East Asia, and Oceania
Gabon	No	Upper middle-income	Sub-Saharan Africa
The Gambia	Yes	Low-income	Sub-Saharan Africa
Georgia	Yes	Upper middle-income	Central Europe, Eastern Europe, and Central Asia
Ghana	Yes	Lower middle-income	Sub-Saharan Africa
Grenada	No	Upper middle-income	Latin America and Caribbean
Guatemala	No	Upper middle-income	Latin America and Caribbean
Guinea	Yes	Low-income	Sub-Saharan Africa
Guinea-Bissau	Yes	Low-income	Sub-Saharan Africa
Guyana	Yes	Upper middle-income	Latin America and Caribbean
Haiti	Yes	Low-income	Latin America and Caribbean
Honduras	Yes	Lower middle-income	Latin America and Caribbean
India	Yes	Lower middle-income	South Asia
Indonesia	Yes	Lower middle-income	Southeast Asia, East Asia, and Oceania
Iran	No	Upper middle-income	North Africa and Middle East
Iraq	No	Upper middle-income	North Africa and Middle East
Jamaica	No	Upper middle-income	Latin America and Caribbean
Jordan	No	Upper middle-income	North Africa and Middle East
Kazakhstan	No	Upper middle-income	Central Europe, Eastern Europe, and Central Asia
Kenya	Yes	Lower middle-income	Sub-Saharan Africa
Kiribati	Yes	Lower middle-income	Southeast Asia, East Asia, and Oceania
Kyrgyzstan	Yes	Lower middle-income	Central Europe, Eastern Europe, and Central Asia
Laos	Yes	Lower middle-income	Southeast Asia, East Asia, and Oceania
Lebanon	No	Upper middle-income	North Africa and Middle East
Lesotho	Yes	Lower middle-income	Sub-Saharan Africa
Liberia	Yes	Low-income	Sub-Saharan Africa
Libya	No	Upper middle-income	North Africa and Middle East
Madagascar	Yes	Low-income	Sub-Saharan Africa
Malawi	Yes	Low-income	Sub-Saharan Africa
Malaysia	No	Upper middle-income	Southeast Asia, East Asia, and Oceania
Maldives	No	Upper middle-income	Southeast Asia, East Asia, and Oceania
Mali	Yes	Low-income	Sub-Saharan Africa
Marshall Islands	No	Upper middle-income	Southeast Asia, East Asia, and Oceania
Mauritania	Yes	Lower middle-income	Sub-Saharan Africa
Mauritius	No	Upper middle-income	Southeast Asia, East Asia, and Oceania
Mexico	No	Upper middle-income	Latin America and Caribbean

Federated States of Micronesia	No	Lower middle-income	Southeast Asia, East Asia, and Oceania
Mongolia	Yes	Lower middle-income	Central Europe, Eastern Europe, and Central Asia
Montenegro	No	Upper middle-income	Central Europe, Eastern Europe, and Central Asia
Morocco	No	Lower middle-income	North Africa and Middle East
Mozambique	Yes	Low-income	Sub-Saharan Africa
Myanmar	Yes	Lower middle-income	Southeast Asia, East Asia, and Oceania
Namibia	No	Upper middle-income	Sub-Saharan Africa
Nepal	Yes	Low-income	South Asia
Nicaragua	Yes	Lower middle-income	Latin America and Caribbean
Niger	Yes	Low-income	Sub-Saharan Africa
Nigeria	Yes	Lower middle-income	Sub-Saharan Africa
North Macedonia	No	Upper middle-income	Central Europe, Eastern Europe, and Central Asia
Pakistan	Yes	Lower middle-income	South Asia
Palestine	No	Lower middle-income	North Africa and Middle East
Papua New Guinea	Yes	Lower middle-income	Southeast Asia, East Asia, and Oceania
Paraguay	No	Upper middle-income	Latin America and Caribbean
Peru	No	Upper middle-income	Latin America and Caribbean
Philippines	No	Lower middle-income	Southeast Asia, East Asia, and Oceania
Moldova	Yes	Lower middle-income	Central Europe, Eastern Europe, and Central Asia
Romania	No	Upper middle-income	Central Europe, Eastern Europe, and Central Asia
Russia	No	Upper middle-income	Central Europe, Eastern Europe, and Central Asia
Rwanda	Yes	Low-income	Sub-Saharan Africa
Saint Lucia	No	Upper middle-income	Latin America and Caribbean
Saint Vincent and the Grenadines	No	Upper middle-income	Latin America and Caribbean
Samoa	No	Upper middle-income	Southeast Asia, East Asia, and Oceania
SÃ£o TomÃ© and PrÃ• ncipe	Yes	Lower middle-income	Sub-Saharan Africa
Senegal	Yes	Lower middle-income	Sub-Saharan Africa
Serbia	No	Upper middle-income	Central Europe, Eastern Europe, and Central Asia
Sierra Leone	Yes	Low-income	Sub-Saharan Africa
Solomon Islands	Yes	Lower middle-income	Southeast Asia, East Asia, and Oceania
Somalia	Yes	Low-income	Sub-Saharan Africa
South Africa	No	Upper middle-income	Sub-Saharan Africa
South Sudan	Yes	Low-income	Sub-Saharan Africa
Sri Lanka	Yes	Upper middle-income	Southeast Asia, East Asia, and Oceania
Sudan	Yes	Lower middle-income	North Africa and Middle East
Suriname	No	Upper middle-income	Latin America and Caribbean
Syria	No	Low-income	North Africa and Middle East
Tajikistan	Yes	Low-income	Central Europe, Eastern Europe, and Central Asia
Thailand	No	Upper middle-income	Southeast Asia, East Asia, and Oceania

Timor-Leste	Yes	Lower middle-income	Southeast Asia, East Asia, and Oceania
Togo	Yes	Low-income	Sub-Saharan Africa
Tonga	No	Upper middle-income	Southeast Asia, East Asia, and Oceania
Tunisia	No	Lower middle-income	North Africa and Middle East
Turkey	No	Upper middle-income	North Africa and Middle East
Turkmenistan	No	Upper middle-income	Central Europe, Eastern Europe, and Central Asia
Uganda	Yes	Low-income	Sub-Saharan Africa
Ukraine	No	Lower middle-income	Central Europe, Eastern Europe, and Central Asia
Tanzania	Yes	Low-income	Sub-Saharan Africa
Uzbekistan	Yes	Lower middle-income	Central Europe, Eastern Europe, and Central Asia
Vanuatu	No	Lower middle-income	Southeast Asia, East Asia, and Oceania
Venezuela	No	Upper middle-income	Latin America and Caribbean
Vietnam	Yes	Lower middle-income	Southeast Asia, East Asia, and Oceania
Yemen	Yes	Low-income	North Africa and Middle East
Zambia	Yes	Lower middle-income	Sub-Saharan Africa
Zimbabwe	Yes	Lower middle-income	Sub-Saharan Africa

Table 8. List of countries included in analysis

Section 9 GATHER Compliance

This study complies with the Guidelines for Accurate and Transparent Health Estimates Reporting (GATHER) recommendations.[3] We have documented the steps involved in our analytical procedures and detailed the data sources used. See Table 3 for the GATHER checklist.

The GATHER recommendations can be found here: http://gather-statement.org/

Table 9. GATHER Compliance Checklist

#	GATHER checklist item	Description of compliance	Reference
Objectives and funding			
1	Define the indicators, populations, and time periods for which estimates were made.	Narrative provided in paper and methods appendix describing indicators, definitions, and populations	Main text (Methods—Overview, Geographical units and time periods) and methods appendix
2	List the funding sources for the work.	Funding sources listed in paper	Main text (Acknowledgments)
Data Inputs			
For all data inputs from multiple sources that are synthesized as part of the study:			
3	Describe how the data were identified and how the data were accessed.	Narrative provided in paper and methods appendix describing data-seeking methods	Main text (Methods) and methods appendix
4	Specify the inclusion and exclusion criteria. Identify all ad-hoc exclusions.	Narrative provided in paper and methods appendix describing inclusion and exclusion criteria	Main text (Methods) and methods appendix
5	Provide information on all included data sources and their main characteristics. For each data source used, report reference information or contact name/institution, population represented, data collection method, year(s) of data collection, sex and age range, diagnostic criteria or measurement method, and sample size, as relevant.	Metadata for data sources by component, activity, geography, currency, currency year, and income classification will be available through an interactive, online data record	Link to the GHDx to be provided upon publication.

6	Identify and describe any categories of input data that have potentially important biases (e.g., based on characteristics listed in item 5).	Summary of known biases included in paper narrative	Main text (Limitations)
For data inputs that contribute to the analysis but were not synthesized as part of the study:			
7	Describe and give sources for any other data inputs.	Will be included in GHDx link	Link to the GHDx to be provided upon publication.
For all data inputs:			
8	Provide all data inputs in a file format from which data can be efficiently extracted (e.g., a spreadsheet as opposed to a PDF), including all relevant meta-data listed in item 5. For any data inputs that cannot be shared due to ethical or legal reasons, such as third-party ownership, provide a contact name or the name of the institution that retains the right to the data.	Downloads of input data available through online tools such as the Global Health Data Exchange website	Online data visualization tools and the Global Health Data Exchange, http://ghdx.healthdata.org
Data analysis			
9	Provide a conceptual overview of the data analysis method. A diagram may be helpful.	Write ups of the overall methodological processes, as well as cause-specific modeling processes, have been provided	Main text (Methods) and methods appendix
10	Provide a detailed description of all steps of the analysis, including mathematical formulae. This description should cover, as relevant, data cleaning, data pre-processing, data adjustments and weighting of data sources, and mathematical or statistical model(s).	Corresponding methodological write-ups have been provided	Main text (Methods) and methods appendix
11	Describe how candidate models were evaluated and how the final model(s) were selected.	Details on evaluation of model performance have been provided	Methods appendix
12	Provide the results of an evaluation of model performance, if done, as well as the results of any relevant sensitivity analysis.	Details on evaluation of model performance have been provided	Methods appendix
13	Describe methods for calculating uncertainty of the estimates. State which sources of uncertainty were,	Details on uncertainty calculations have been provided	Methods appendix

	and were not, accounted for in the uncertainty analysis.		
14	State how analytic or statistical source code used to generate estimates can be accessed.	Access statement provided	Code is provided in an online repository, code will be provided at https://github.com/ihmeuw/Resource_Tracking_Domestic_Health_Accounts upon publication
Results and Discussion			
15	Provide published estimates in a file format from which data can be efficiently extracted.	Results are available through the Global Health Data Exchange	Link to the GHDx to be provided upon publication.
16	Report a quantitative measure of the uncertainty of the estimates (e.g. uncertainty intervals).	Uncertainty intervals are provided with all results	Main text, methods appendix, and online data tools (the Global Health Data Exchange, link to the GHDx to be provided upon publication.)
17	Interpret results in light of existing evidence. If updating a previous set of estimates, describe the reasons for changes in estimates.	Discussion of methodological differences between our estimates and other available evidence provided in the paper and methods appendix	Main text (Methods and Discussion) and methods appendix
18	Discuss limitations of the estimates. Include a discussion of any modelling assumptions or data limitations that affect interpretation of the estimates.	Discussion of limitations was provided	Main text (Limitations) and methods appendix

Section 10. References

1. Institute for Health Metrics and Evaluation (IHME). **Financing Global Health 2019: Tracking Health Spending in a Time of Crisis – Supplementary Methods Annex**. Seattle, WA: IHME, 2020. (http://www.healthdata.org/sites/default/files/files/policy_report/FGH/2020/FGH-Report-2019_Methods-Appendix.pdf)

2. Global Burden of Disease Health Financing Collaborator Network. Health spending for Sustainable Development Goal 3: total, HIV/AIDS, tuberculosis, malaria, universal health service coverage, financial risk protection, and donor spending, 2000 to 2030 and 2050. 2019; published online Dec 13.

3. Stevens GA, Alkema L, Black RE, et al. Guidelines for Accurate and Transparent Health Estimates Reporting: the GATHER statement. *Lancet*. 2016;388(10062):e19-e23. doi:10.1016/S0140-6736(16)30388-9
